# Epidemiology of Influenza A virus in Swiss pig herds: subclinical circulation and associated risk factors

**DOI:** 10.1186/s40813-026-00513-5

**Published:** 2026-04-25

**Authors:** Jonas Steiner, Bernhard Voelkl, Mike Mwanga, Larise Oberholster, Matthias Licheri, Manon Flore Licheri, Ronald Dijkman, Jenna Nicole Kelly, Heiko Nathues

**Affiliations:** 1 Clinic for Swine, Department of Clinical Veterinary Medicine, Vetsuisse Faculty, University of Bern, Bern, Switzerland; 2 Multidisciplinary Center for Infectious Diseases, University of Bern, Bern, Switzerland; 3 Graduate School for Cellular and Biomedical Sciences, University of Bern, Bern, Switzerland; 4 Veterinary Public Health Institute, University of Bern, Bern, Switzerland; 5 Institute for Infectious Diseases, University of Bern, Bern, Switzerland; 6 Institute of Virology and Immunology, Bern and Mittelhäusern, Switzerland; 7 Department of Infectious Diseases and Pathobiology, Vetsuisse Faculty, University of Bern, Bern, Switzerland

**Keywords:** Swine influenza, Cross-sectional study, Nasal swabs, Pan-IAV qPCR, Risk factors - Switzerland

## Abstract

**Background:**

Influenza A viruses (IAVs) are important respiratory pathogens of pigs, impacting both animal health and productivity. However, their epidemiology in Swiss pig herds remains poorly understood. Therefore, we conducted a cross-sectional study encompassing 100 pig herds, combining semi-structured interviews, clinical examinations, and nasal swab sampling analyzed by pan-IAV qPCR.

**Results:**

Overall, 35% of herds with mean intra-herd detection rate of 8.5% tested positive for IAV. Among herds without clinical respiratory signs, 34.1% (29/85) were IAV-positive. Exploratory analysis suggested trends toward higher IAV detection in herds with more intensive production or biosecurity shortcomings. Factors such as contact with other animal species or mixing between age groups may facilitate viral circulation.

**Conclusion:**

IAV is commonly detected in Swiss pig herds and often occurs without causing clinical signs. Epidemiological patterns are similar to those reported elsewhere in Europe, despite Switzerland’s small-scale and less intensified production sites, generally lower biosecurity standards, and nearly non-existent cross-border live pig transport. Although the clinical impact of IAV was limited, inapparent influences on production performance warrant implementation of IAV control measures. Follow-up studies are needed to better understand circulation dynamics of IAV in Swiss pig herds.

**Supplementary Information:**

The online version contains supplementary material available at 10.1186/s40813-026-00513-5.

## Background

Influenza A viruses (IAVs) are widely prevalent in pig populations worldwide [1]. Their infection dynamics are rapidly evolving due to intensification of pig farming and IAV has an undeniable but not yet fully understood impact on pig health. These developments occur against the background of continuous human-pig interactions, in which humans in close contact with pigs face a potentially increased zoonotic risk [2, 3].

The epidemiology of IAV is becoming increasingly complex. The emergence of the H1N1 2009 pandemic (H1N1pdm09) lineage has contributed to diversification of IAV clades circulating in European pig herds [4]. In parallel, IAV intra-herd detection across Europe is high, with a mean seropositivity rate of 58.7% reported in a meta-analysis including 42 studies [5]. Endemic IAV circulation within intensive pig production systems makes virus eradication challenging despite the availability of vaccination-based control measures [6, 7]. The clinical manifestations of IAV infection are highly variable [8], ranging from asymptomatic circulation to mild, easily overlooked respiratory outbreaks in endemically infected herds, up to severe respiratory disease. IAV can contribute to increased mortality, when secondary infections occur [9, 10]. These conditions are collectively known as the Porcine Respiratory Disease Complex (PRDC). Within the PRDC, Influenza A virus, Porcine Reproductive and Respiratory Syndrome Virus (PRRSV), *Mycoplasma hyopneumoniae* and *Actinobacillus pleuropneumoniae* (APP) act as primary pathogens, whereas *Streptococcus suis*, *Glaesserella parasuis* or *Pasteurella multocida* play a role as secondary or opportunistic pathogens.

Subclinical IAV infections in pigs are increasingly recognized, both within commercial pig herds and at agricultural fairs where they have contributed to zoonotic transmission to humans [6, 11–15]. In endemically infected herds, complex infection dynamics lead to inconsistent clinical expression and intermittent virus detection, with variation by age group. Weaned piglets, particularly during the early nursery period, have been identified as key contributors to herd-level IAV circulation [16, 17]. Beyond their epidemiological relevance, subclinical IAV infections have been associated with reduced growth performance and weight gain in fattening pigs [18]. Consequently, subclinical IAV infections have a substantial impact on pig health and production, and understanding their dynamics is essential for elucidating overall IAV circulation and informing effective control strategies.

As an important basis for designing control measures, several studies have previously established risk profiles for IAV infection. Many of these relied on serology to define IAV-positive cases [15–18], which must be interpreted with caution, since antibodies may be maternally derived or vaccine-induced, thereby reducing the specificity of such methods for detecting true field infections. The most prominent risk factors have been consistently identified across multiple studies [19–26]: These include herd size and pig density within and around farms, pig contact with other mammalian species or birds, insufficient separation between age groups, lack of sanitary downtime between batches, and shortcomings in hygiene practices.

Switzerland’s pig production has distinct characteristics, with a predominantly small-scale farming system compared to other central European countries [27]. Biosecurity standards in Switzerland are among the lowest in Europe [28]. However, the country has been able to eradicate several important swine pathogens due to the near absence of live pig imports (approximately 0.07% of the national pig population per year) [29]. Consequently, Switzerland is officially free from PRRSV [30], Enzootic Pneumonia as well as *Actinobacillus* caused pleuro-pneumonia. As a result, the intra-herd detection of *Mycoplasma hyopneumoniae* is extremely low, with only 0.98% of sows testing seropositive in a representative national slaughterhouse survey in 2022 [31]. This situation may reduce the occurrence of PRDC and alter the clinical impact of IAV in Swiss pig herds. At the same time, Switzerland may represent an “epidemiological island” for circulating pathogens, with potentially distinct transmission dynamics resulting from its import restrictions and unique production system characteristics.

To investigate previously underexplored IAV circulation in Switzerland, a passive syndromic surveillance program was established in 2001 by the Swiss Federal Food Safety and Veterinary Office (FSVO) and the Swiss Federal Office of Public Health (FOPH), in collaboration with one of the Swiss pig health services (SUISAG-SGD) and the University of Zurich [32]. Between 2010 and 2022, 674 respiratory outbreak events have been documented and tested for IAV. 375 examinations (55.6%) were IAV-positive. However, this surveillance is limited to detecting IAV presence and identifying strains in herds affected by respiratory diseases, as sampling relies on nasal swabs from only two coughing pigs per outbreak and metadata collection has been insufficient. Two closely related H1N1 clades of Eurasian avian like (EA) lineage, 1.C.2.1 and 1.C.2.2, were identified, appearing to form an independent Swiss cluster. IAVs belonging to the H1N1pdm09 lineage were identified in only seven outbreaks, including a single sample assigned to clade 1.A.3.3.2, which is also detected in neighbouring European countries. Still, data overall suggest largely isolated IAV circulation within Swiss pig herds [32]. Nevertheless, the absence of sampling in clinically healthy herds, a suboptimal sampling strategy, and the lack of epidemiologic characterization leave significant gaps in our understanding of IAV dynamics in the distinct Swiss pig population.

Therefore, as part of a multidisciplinary consortium within the Multidisciplinary Center of Infectious Diseases, University of Bern, the Clinic for Swine, Vetsuisse Faculty, University of Bern conducted a cross-sectional study in 100 Swiss pig herds - encompassing both clinically healthy and respiratory-diseased herds - to assess associations between IAV detection by qPCR from nasal swabs and husbandry, animal and human health-related factors. The objectives of the study were to (i) provide an overview of IAV detection rates in Swiss pig herds and (ii) determine whether risk and protective factors for IAV detection identified in neighboring European countries apply to the Swiss pig population.

## Methods

### Study design and setting

**Fig. 1:**
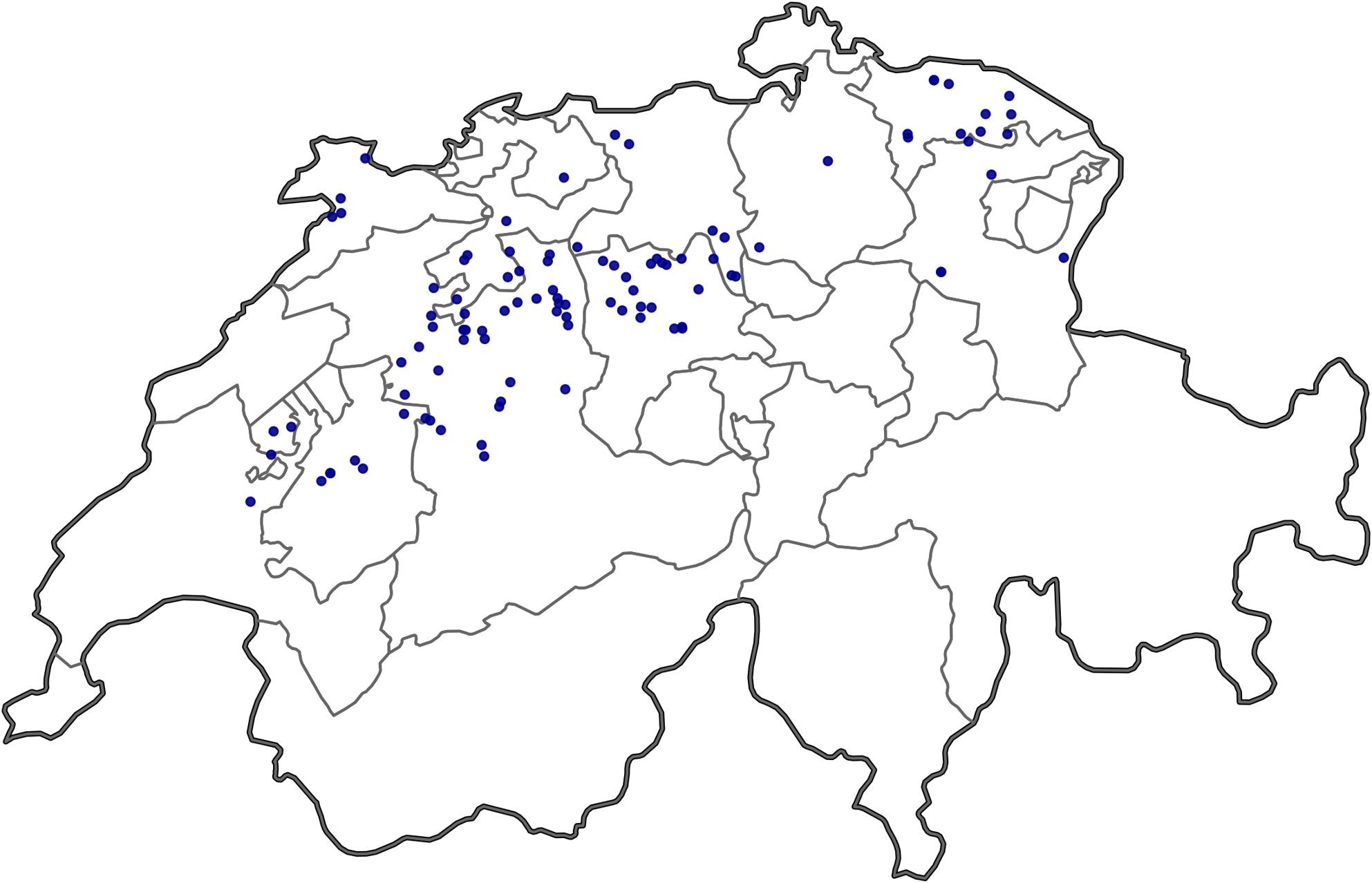
Map of Swiss pig herds included in the study

A cross-sectional study of 100 pig herds, mainly located in pre-alpine Swiss cantons with the highest pig density (Fig. 1), was conducted between May 2024 and May 2025. Herds were actively recruited as a convenience sample, with no previously reported history of respiratory disease. Herd managers provided informed consent for participation. No exclusion criteria were defined regarding herd size or production type. Data were collected at the herd level during a single on-site examination and supplemented by a follow-up call three months later. The study was approved under Swiss national animal experiment number 35,801 and is reported in accordance with the STROBE-Vet guidelines [33].

### Data collection

**Fig. 2:**
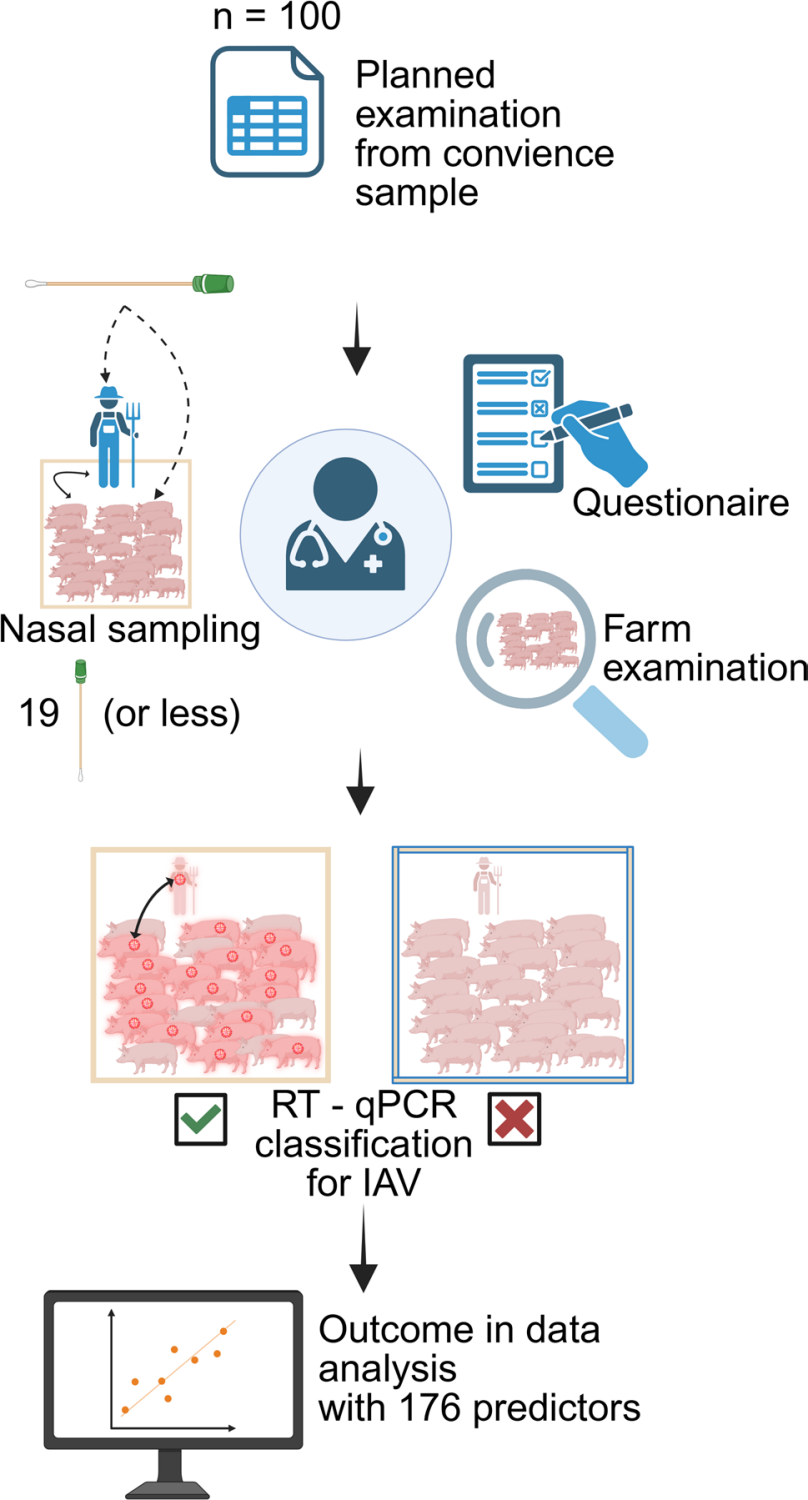
Study design of the Swiss cross-sectional study on IAV. A total of 100 herds were recruited through a convenience sample. During each on-site observation, a semi-structured questionnaire with the herd manager was conducted, clinical examination of pigs was performed, and nasal swabs from pigs were collected from 19 (or less depending on herd size) samples. Laboratory classification was based on detection of influenza A virus (IAV) RNA using a pan-IAV matrix segment–specific RT-qPCR assay, serving as the outcome variable for subsequent data analyses

During each on-site examination performed by a licensed veterinarian, a semi-structured interview with the herd manager was conducted, the pig herd was clinically examined, and nasal swabs were collected (Fig. 2).

A written questionnaire was read aloud to the herd manager. Questions were developed based on previous studies on IAV risk factors [19, 20, 22, 24, 25, 34, 35] and through informal consultation with two experts from neighboring European countries. They covered farm characteristics, biosecurity, hygiene, personnel and visitor management, and respiratory disease history in pigs and herd managers. The questionnaire is provided in Additional file 1 as a German version. The questionnaire was piloted with three herd managers, and minor adjustments were made for clarity. Herd managers’ responses were entered into an Access^®^ (Microsoft Corporation, Redmond, USA) database. After the interview, data underwent plausibility checks and assessments of missing data. At least three months after the initial on-site examination, a follow-up call was conducted to assess respiratory disease status after initial sampling, verify and correct implausible data and collect any missing information.

The clinical herd examination was performed based on a standardized checklist focusing on respiratory disease signs (Additional file 1, in German). The entire herd was screened, and a coughing and sneezing index was calculated for the sampled age group only according to established standards [36]. The cut-offs for sneezing indices were based on field experience, as no universally accepted cut-off values are established. Rectal body temperature was measured in pigs selected for sampling, and fever cut-offs were based on field experience, as measured values consistently exceeded published thresholds [37]. A herd was clinically classified as respiratory diseased if at least one of the following signs was detected: (i) acute: a maximum rectal body temperature of ≥ 40.5 °C in at least one of the sampled pigs in combination with at least one of the following: a sneezing index of ≥ 20%/min i weaners or fattening pigs, or the observation of apathy, according to subjective assessment, in ≥ 5% of weaners; (ii) subacute: a coughing index of ≥ 2.5%/min i weaners, according to [36].

The nasal sampling of pigs was conducted using a sample size calculated by WinEpi (http://www.winepi.net/uk/index.htm) to ensure detection of IAV at herd level in at least one individual with 95% confidence. The weaner age group (4–12 weeks) is assumed to have the highest likelihood of herd-level detection of IAV via nasal swabs [17] and was therefore selected for sampling in the nursery or upon entry into the fattening unit. In Swiss pig herds, weaners from different batches are typically housed in the same airspace. Therefore, preferably the batch of most recently weaned piglets (4–5 weeks) was selected. However, if these were not available, the next oldest batch was selected. Per herd, 19 weaners were randomly sampled across all pens, assuming a pig level IAV intra-herd detection of ≥ 15% the Swiss legal maximum herd size (1500 fattening pigs). The sample size was adjusted accordingly for herds with < 400 weaners. Each pig was manually restrained and a nasal swab (FLOQSwab^®^; Copan, Brescia, Italy) was taken from one nostril. After wiping the snout with a dry paper towel, the swab was inserted along the nasal septum in dorso-caudal orientation until slight resistance was felt, and then rotated three times by 120 degrees, resting three seconds at each position [38]. The swabs were then carefully inserted into individual tubes containing the NucleoProtect^®^ conservation medium (Macherey-Nagel, Düren, Germany), transported at ambient temperature, and stored at ≤ -20 °C until further processing.

Viral RNA was extracted at the Institute of Infectious Diseases, University of Bern (IFIK) using the NucleoMag VET^®^ kit on a KingFisher Apex instrument, following the manufacturer’s instructions, and subsequently screened for IAV RNA by a pan-IAV matrix segment–specific RT-qPCR with optimized reaction conditions as previously described [39, 40]. Only Samples with a crossing point (Cp) value < 38 were considered positive [17]. Each run included a negative control containing conservation medium.

### Variables

All variables were analyzed at the herd level. Measurements collected at the animal level (e.g., RT-qPCR results, rectal body temperatures) were aggregated into herd-level indicators. The outcome variable was defined as herd IAV status (IAV-positive), where herds were classified as positive if at least one sample tested positive in RT-qPCR with a Cp < 38 [17]. Seven additional meta-variables were included to further characterize the RT-qPCR results but were not used as exposure variables. A total of 202 exposure variables were initially included, derived from questionnaire responses and clinical examination (codebook in Additional file 2). To facilitate interpretation, variables were subsequently grouped into four thematic domains:

1. Husbandry: herd size, production type, pig flow, hygiene, internal and external biosecurity, personnel and visitor management.
2. Animal health: clinical symptoms reported by the herd manager and clinical signs observed by the licensed study veterinarian, rectal body temperature measurements, aggregated to maximum and mean herd-level value.
3. Environment: measurements related to stable building, housing temperature, airflow, and pen stocking density.
4. Human health: reported respiratory history of the herd manager, employees, and household members.

To assess potential confounding across all predictors from the four domains, an overall correlation matrix was screened for pairwise correlations ≥ 0.70.

### Statistical methods

The exploratory statistical analysis of the study dataset included descriptive statistics on the initial dataset, followed by univariate analyses of associations and correlations between exposure variables within each domain and herd IAV status, using a cleaned dataset. Partial Least Squares Discriminant Analysis (PLS-DA) was performed for each domain to capture more complex multivariate interactions in the data. Exposure variables with highest variable importance in projection (VIP) scores in PLS-DA and biological relevance, were subsequently included in a logistic regression model (see Additional file 8 for justifications for excluding variables). All statistical analyses were conducted in R, version 4.4.2, with ChatGPT 5.2 used to support the coding. The code of the data analysis can be found in the GitHub repository https://github.com/jonasalexandersteiner/IAV_herds_presumably_non_diseased.

Descriptive statistics were computed for all variables and summarized to guide subsequent analyses. Based on this, factor variables with < 5 observations per level were collapsed, redundant variables as well as those with no variance or only missing values were excluded. As a result, 176 exposure variables and one binary outcome variable remained. Eight meta-variables describing the outcome were retained for reference but were not included in the statistical analysis. The descriptive results guided the choice of subsequent inferential tests.

Next, univariate associations were tested between the exposure variables grouped into thematic domains and the outcome herd IAV status: 89 in husbandry, 46 in animals, 30 in environment, 16 in human. The variables farrowing on farm, insemination on farm, gestation on farm, weaners on farm, and fattening on farm were included in the first three domains, and season of sampling was considered in both the environment and human health domains to ensure coverage across all relevant settings. Six variables closely tied to the overview measure were excluded from inferential testing to avoid double-counting. Univariate associations between each exposure variable and the outcome were assessed using appropriate statistical tests based on variable data type. The p-values were adjusted for multiple comparisons using the Benjamini-Hochberg method (false discovery rate, FDR) within each domain. Results were summarized, along with the sample size and raw and FDR-corrected p-values. Subsequently, correlations among variables were similarly evaluated. Only the variables with the strongest associations (lowest raw p-values) are presented in the tables, for full results refer to Additional file 5.

To assess dependence among predictors and screen for collinearity, a correlation matrix of all predictors was computed using methods appropriate to their variable types. Correlation coefficients ≥ 0.70 were flagged and considered during interpretation only when biologically plausible, recognizing that spurious correlations can arise given the large number of predictors. The full correlation matrix is provided in Additional file 6.

Given the large number of predictors, the limited sample size (*n* = 100), and the absence of significant findings in univariate testing, Partial Least Squares Discriminant Analysis (PLS-DA) was used to further explore candidate risk and protective factors [41]. All variable data were transformed into binary or nominal types and dummy-encoded as necessary. Predictors were filtered before modeling by removing variables with constant values, low variance, duplication or high correlation (> 0.95) with other predictors to ensure stability and interpretability. All predictors were grouped into the previously defined domains and standardized. PLS-DA models were fitted for each domain. Optimal model complexity was determined via 5-fold cross-validation (random seed 123). Variable importance in projection (VIP) scores, and predictor loadings on the first two components were extracted. Model performance (receiver operating characteristic (ROC) curve and its area under the curve (AUC), sensitivity, specificity, and balanced accuracy) was estimated using cross-validation [42].

Predictors with strongest trend toward association with IAV herd status were included into multivariable logistic regression, after selection based on the PLS-DA. This analysis was restricted to the husbandry domain due to its biological relevance. Predictors were ranked in descending order of their VIP scores [43], those with biologically implausible associations were excluded, and the next plausible predictor in the ranking was selected (see Additional file 8). To maximize model stability and interpretability, four predictors were included, consistent with the recommended events-per-variable guideline (35 events in IAV-positive) [44]. No missing data were present among the selected predictors. The final logistic regression model was fitted using standard maximum likelihood estimation. Model performance was evaluated in terms of explanatory power (Pseudo-R²), calibration (Hosmer–Lemeshow goodness-of-fit test and calibration plot), and discrimination (ROC curve and its AUC) [45].

## Results

In this nationwide cross-sectional study of Swiss pig herds, predominantly located in the pre-alpine regions with high pig density, 176 exposure variables were analyzed for their association with herd IAV status (35 IAV-positive and 65 IAV-negative herds). Trends toward increased IAV detection in larger herds and in herds reporting biosecurity shortcomings were consistently observed across all analyses, although none were significant (*p* < 0.05) after multiple testing correction in the univariate analysis.

### Descriptive data on exposures

Descriptive statistical analyses revealed substantial heterogeneity across the study population, including a wide range of pig herd production types and herd sizes. Differences in age groups and management practices introduced bias and resulted in non-random missing data. Missing data were influenced by both the age groups present and by the unavailability of certain measurements. Greater detail is presented for the husbandry and animal health predictors, which included the largest number of exposure variables, whereas the other predictor domains (environmental, human health) are summarized more briefly. Full descriptive statistics are available in Additional file 3, and the codebook is available in Additional file 2.

In the husbandry domain, herd size averaged 843 +/- 587 pigs (87 +/- 77 sows or 283 +/- 302 fattening pigs). Production types included 11 fattening pig herds, 25 farrow-to-finish herds, 41 breeding herds, 14 integrated piglet producing herds, and 9 nucleus or multiplier herds. Herds had a mean of 1.7 +/- 0.7 supplier herds.

Overall, 34% lacked a concept of quarantine. 67% of herds were in proximity (less than 1000 m) to another pig herd, and poultry was present on 33% of farms, with access to the pig stable in 7% of farms.

In farrowing units, room-wise all-in-all-out (AIAO) was practiced in 73% of herds. Cross-fostering was practiced in < 10% of litters in 21% of herds, in ≤ 50% of litters in 54% of herds, and in > 50% of litters in 25% of herds. Farrowing units were washed after each batch in 96% of herds, and disinfected after each batch in 38%, ≥ 24 h of downtime between batches was reported in 87% of herds. In the nursery, AIAO was practiced in 53% of herds, cleaning between batches in 88%, disinfection in 31%, and ≥ 24-hour downtime was maintained in 90%. In contrast, for the fattening units, AIAO was less common (14% of herds); cleaning after each batch was practiced in 22%, disinfection in 24%, and ≥ 24 h of downtime was maintained by 21% of herds.

The biosecurity assessment revealed that 75% of herds had an outside area, allowing for bird contact in 69% and wild boar contact in 55%. Bird contact inside the stable was reported in 39% of herds. There were about 2.9 caretakers per herd; 65% wore dedicated stable clothing, 45% washed their hands before entering the stable, 12% wore masks when sick, and 22% performed boot disinfection. Contact with other pig herds was reported by 8% of caretakers. Herds had a mean of 7.0 +/- 4.9 cumulative visitor contact hours per year; 50% of visitors washed their hands upon entry, and 88% had contact with other pig herds on the same day.

In the animal domain, respiratory disease signs were observed in 15% of herds. Reported symptoms included primarily coughing, apathy, and reduced feed intake, mostly in weaners and fattening pigs. Clinical examination yielded coughing and sneezing indices that were significantly higher in herds with respiratory signs compared to clinically healthy ones (Table 1). Rectal body temperatures did not differ significantly. A historical seasonal pattern of respiratory symptoms was reported by caretakers in 90% of herds, predominantly in autumn and spring. Specifically, multiple outbreaks (> 3 per year) were reported on 4% of farms, 1–2 outbreaks per year in 18%, and less often or none in 78%. In the 3-month follow-up call, 5% of farms reported respiratory disease outbreaks within 3 months after the study examination.

**Table 1:** Clinical respiratory signs observed in study herds

Clinical sign	Herd respiratory status	NA %	Mean	95% CI	*p*-value (Wilcoxon)
Sneezing index (% of pigs/min)	Clinically inapparent	0	4.4	[3.3, 5.5]	**< 0.01**
	Respiratory signs observed	0	15.1	[6.8, 23.5]	
	All herds	0	6.0	[4.4, 7.7]	
Coughing index (% of pigs/min)	Clinically inapparent	0	0.3	[0.2, 0.4]	**< 0.01**
	Respiratory signs observed	0	4.5	[3.5, 5.5]	
	All herds	0	0.9	[0.6, 1.3]	
Mean herd level rectal body temperature (°C)	Clinically inapparent	1.2	39.7	[39.6, 39.7]	0.57
	Respiratory signs observed	0	39.7	[39.5, 39.9]	
	All herds	1	39.7	[39.6, 39.7]	
Maximum herd level Rectal body temperature (°C)	Clinically inapparent	1.2	40.4	[40.3, 40.5]	0.124
	Respiratory signs observed	0	40.5	[40.3, 40.8]	
	All herds	1	40.4	[40.3, 40.5]	

Sneezing and coughing indices and herd-level rectal body temperatures (maximum and mean) are reported as means with confidence intervals (95% CI), stratified by respiratory signs and overall. Confidence intervals for means use t-based estimates. The percentage of missing values (NA%) is reported. Between-group differences were assessed using the Wilcoxon Mann–Whitney test; significant two-sided p-values (*p* < 0.05) are shown in bold

In brief, in the environmental domain (full descriptive statistics in Additional file 3), temperature and airflow measurements were influenced by the sampling season. Due to variability in farm types and missing measurements in numerous herds, the explanatory power of environmental variables is limited. In the human domain (full descriptive statistics in Additional file 3), respiratory symptoms were reported in caretakers and contact persons on 12 of 100 farms, less frequently during summer, consistent with the seasonal pattern reported in pigs.

### Descriptive data on IAV detection

Overall, 35% of herds tested positive for IAV, with a mean intra-herd detection rate of 8.5% (sample-specific IAV qPCR results are provided in Additional file 4). Overall IAV detection rates and mean intra-herd detection rate were lower in clinically inapparent herds than in herds with respiratory signs (detection: 34.1% vs. 40.0%; mean intra-herd detection: 6.8% vs. 18.0%), but these differences were not statistically significant (Chi-squared test for detection: *p* = 0.66; Wilcoxon Mann–Whitney test for intra-herd detection: *p* = 0.33). Cp values were lower in herds with respiratory signs, consistent with higher viral RNA levels, although the difference did not reach statistical significance (Welch’s t-test for the mean: *p* = 0.06). Herd-level detection rates and mean intra-herd detection rates for both groups are shown in Table 2. Viral loads are presented as semi-quantitative Cp-values. The proportion of IAV-positive herds was similar across production types and across sampled age groups, with no significant differences (Fisher’s exact test: production type, *p* = 1.00; age group, *p* = 0.45); details are presented in Tables 3 and 4.

**Table 2:** Detection of influenza A virus (IAV) at the herd level, mean herd-level intra-herd detection, and viral load (mean Cp) stratified by the presence of respiratory signs

Herd-level detection (IAV-positive herds)	herds	IAV-positive herds	Estimate (%)	95% CI
All herds	100	35	35.0	[26.4, 44.7]
Herds with respiratory signs	15	6	40.0	[19.8, 64.3]
Herds without respiratory signs	85	29	34.1	[24.9, 44.7]
**Mean intra-herd detection rate**				
All herds	100		8.5	[4.9, 12.7]
Herds with respiratory signs	15		18.0	[4.9, 34.0]
Herds without respiratory signs	85		6.8	[3.6, 10.9]
**Herd-level Cp-value**			**Mean Cp-value**	**95% CI**
All herds		35	34.7	[33.8, 35.6]
Herds with respiratory signs		6	33.0	[31.0, 35.0]
Herds without respiratory signs		29	35.0	[34.0, 36.0]

Herd counts per group are indicated. Percentages are point estimates. Herd-level detection rate 95% confidence intervals (CIs) use the Wilson score method; mean intra-herd detection rates with 95% confidence intervals were estimated using percentile bootstrap resampling (B = 10,000). Mean Cp 95% CIs are based on Welch’s two-sample t-test interval. CIs are reported as [lower, upper]

**Table 3:** IAV detection by age group of sampled pigs

Age group (woa)	Herds sampled	IAV-positive herds	Estimate (%)	95% CI
4–5	55	20	36.4	[24.9, 49.6]
6–7	17	6	35.3	[17.3, 58.7]
8–9	4	1	25	[4.6, 69.9]
10–12	12	4	33.3	[13.8, 60.9]
Mixed 4–12	12	4	33.3	[13.8, 60.9]

Estimates are the percentage of herds that were IAV-positive, with 95% confidence intervals (CI) calculated using the Wilson score method. Counts per age group (number of herds and IAV-positive herds) are shown; CIs are reported as [lower, upper]. “woa” denotes weeks of age

**Table 4:** IAV detection across production types

Production Type	Number of herds	IAV-positive herds	Estimate (%)	95% CI
Fattening herd	11	4	36.4	[15.6, 64.6]
Farrow to finish herd	25	5	20	[8.9, 39.1]
Breeding herd	41	16	39	[25.7, 54.3]
Integrated piglet production herd	14	6	42.9	[21.4, 67.4]
Nucleus or multiplier herd	9	4	44.4	[18.9, 73.3]

Estimates are the percentage of herds that were IAV-positive, with 95% confidence intervals (CI) calculated using the Wilson score method. Counts per production type (number of herds and IAV-positive herds) are shown; CIs are reported as [lower, upper]

### Trends in univariate association analyses

Univariate associations and correlations between exposure variables and the outcome herd IAV status (IAV-positive) were tested separately for each thematic domain (husbandry, animal health, environment, and human). All p-values were adjusted for multiple testing using the false discovery rate (FDR) correction. Below, for conciseness, the tables show only those variables with uncorrected p-values of < 0.2. Complete results are provided in Additional file 5.

In the husbandry domain (Table 5 for predictors with *p* < 0.2, full domain in Additional file 5), predictors related to production intensity (number of suckling piglets, restocking or birth interval between batches), and factors that may increase inter-species or inter-age group contact showed the strongest trends of association with herd IAV status. Cross-fostering practices and respiratory problems in supplier herds were also among the variables showing notable trends. Notably, some predictors (e.g., caretaker only entering with dedicated clothing, bird nests present in stable, weaners stable cleaned) displayed directions of correlations inconsistent with biological expectations. None of the variables remained statistically significant after correction for multiple testing (all FDR-adjusted *p* > 0.05).

**Table 5:** Univariate associations and correlations of husbandry predictors with the outcome IAV-positive with raw p-values < 0.2

Variable	*N*	Test	*p*-value	FDR *p*-value	Correlation	Test
Contact with birds in the stable	100	Chi-squared	< 0.01	0.22	0.32	Spearman
Insemination room: airspace with other age group	57	Fisher’s exact	< 0.01	0.22	0.43	Cramér’s V
Restocking or birth interval between batches	100	Chi-squared	0.06	1	0.27	Cramér’s V
Weaners stable cleaned	90	Fisher’s exact	0.09	1	0.2	Spearman
Respiratory symptoms reported in supplier herd	100	Fisher’s exact	0.12	1	0.17	Spearman
Caretaker enters stable with dedicated clothes only	100	Chi-squared	0.13	1	0.17	Spearman
Bird nests present in stable	100	Chi-squared	0.14	1	-0.17	Spearman
Number of suckling piglets	88	t-test	0.16	1	0.19	Spearman
Intensity of cross fostering practiced	89	Chi-squared	0.17	1	0.2	Cramér’s V
Farrowing stable: airspace with other age group	85	Fisher’s exact	0.18	1	0.21	Cramér’s V

Predictors are listed with their samples size (maximum 100), the statistical test used for assessing univariate association of predictors with the binary outcome IAV-positive, its raw value and its p-value corrected for the false discovery rate (FDR) according to the Benjamini-Hochberg method. Correlations are reported with Cramér’s V for factors and Spearman’s rho for binary, continuous or ordinal predictors

In the animal health domain (Table 6 for predictors with raw *p* < 0.2, full domain in Additional file 5), recent history of respiratory symptoms in pigs reported by the herd manager showed a strong trend toward association with IAV detection (*p* < 0.1). Several other predictors, weaners as affected age group and clinical signs of respiratory disease (nasal discharge, dyspnea), were also relevant (all *p* < 0.2). Correlations of percentage of sneezing litters displayed were inconsistent with biological expectations. None of the variables remained statistically significant after correction for multiple testing (all FDR-adjusted *p* > 0.05).

**Table 6:** Univariate associations and correlations of animal health predictors with the outcome IAV-positive with raw p-values < 0.2

Variable	*N*	Test	*p*-value	FDR *p*-value	Correlation	Test
Percentage sneezing litters in farrowing	85	Wilcoxon	0.04	1	-0.22	Spearman
History of respiratory disease	79	Fisher’s exact	0.08	1	0.25	Cramér’s V
Reported disease in weaners	100	Chi-squared	0.11	1	0.18	Spearman
Dyspnea in pigs reported	100	Fisher’s exact	0.13	1	-0.16	Spearman
Nasal discharge reported in pigs	100	Fisher’s exact	0.18	1	0.17	Spearman

Predictors are listed with their samples size (maximum 100), the statistical test used for assessing univariate association of predictors with the binary outcome IAV-positive, its raw value and its p-value corrected for the false discovery rate (FDR) according to the Benjamini-Hochberg. Correlations are reported with Cramér’s V for factors and Spearman’s rho for binary, continuous or ordinal predictors

In the environment domain (Table 7 shows predictors with *p* < 0.20; full domain in Additional file 5), temperature measures related to sows and stocking density of weaners showed the strongest trends toward association with IAV detection (all *p* < 0.20), but none remained significant after Benjamini–Hochberg FDR correction. Temperature and airflow measurements across compartments were highly correlated (coefficients ≥ 0.70) and were also correlated with observed coughing in fattening pigs, which is considered during interpretation. A substantial portion of missingness reflected structurally missing data arising from the absence of specific compartments on certain farms, depending on production type.

**Table 7:** Univariate associations and correlations of environmental predictors with the outcome IAV-positive with raw p-values < 0.2

Variable	*N*	Test	*p*-value	FDR *p*-value	Correlation	Test
Insemination temperature	46	t-test	0.02	0.53	-0.3	Spearman
Gestation temperature	61	t-test	0.04	0.53	-0.25	Spearman
Weaners per feeding site	94	Fisher’s exact	0.15	1	0.26	Cramér’s V
Farrowing temperature	72	t-test	0.18	1	-0.13	Spearman

Predictors are listed with their samples size (maximum 100), the statistical test used for assessing univariate association of predictors with the binary outcome IAV-positive, its raw value and its p-value corrected for the false discovery rate (FDR) according to the Benjamini-Hochberg. Correlations are reported with Cramér’s V for factors and Spearman’s rho for binary, continuous or ordinal predictors

In the human domain (full domain in Additional file 5), the sampling season and respiratory history reported in humans and showed trends toward association with herd IAV detection (Fisher’s exact p values = 0.33 and 0.21; FDR p values = 0.84; Spearman correlations = 0.21 and 0.12). No predictor remained significant after FDR correction.

### Multivariate associations and post-hoc analysis with PLS-DA

**Fig. 3:**
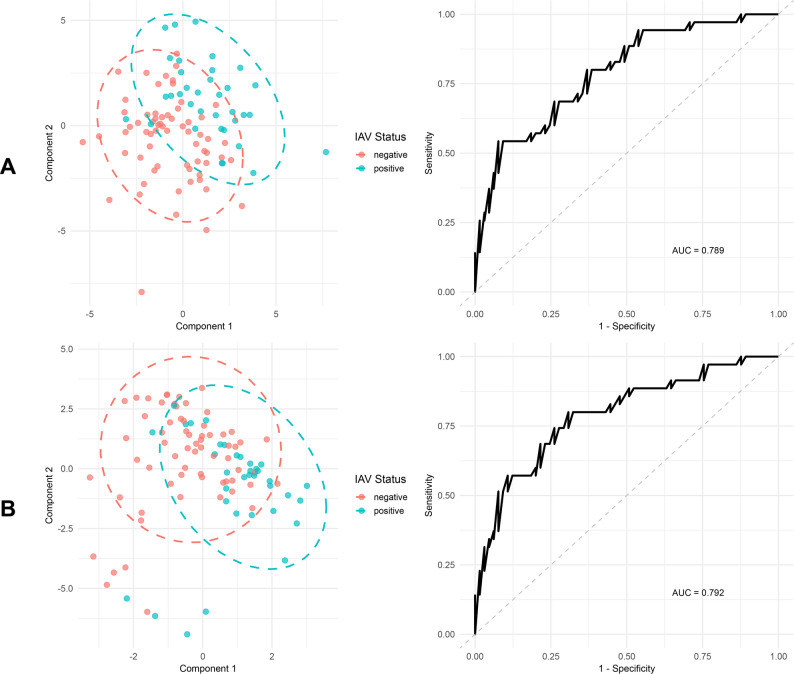
Sample score and receiver operating characteristic (ROC) plots of Partial Least Squares – Discriminant Analysis models for (**A**) husbandry and (**B**) animal health predictors. Panel **A** shows data for the husbandry domain, while panel **B** shows data for the animal health domain. Sample score plots display all herds with loadings on component 1 (x-axis) and component 2 (y-axis). Clustering according to influenza A virus (IAV) detection status is indicated by dashed lines and colors (blue for IAV-positive and red for IAV-negative herds). ROC curves display 1 – specificity (false positive rate) on the x-axis and sensitivity (true positive rate) on the y-axis. The area under the curve (AUC) is indicated numerically within each plot

Given the large number of predictors, limited sample size, and absence of significant findings in univariate analyses, Partial Least Squares Discriminant Analysis (PLS-DA) was applied to explore multivariate association patterns. The results are exploratory and do not entail statistical significance testing. Model performances are summarized in Fig. 3; balanced accuracy was 0.733 in the husbandry and 0.724 in the animal health domain. Additional detailed performance metrics, including the confusion matrix and full analysis results are provided in Additional file 7, the codebook is provided in Additional file 2.

In the husbandry domain (Table 8 shows predictors with VIP > 1.5; see Additional file 7 for the full set), predictors related to interspecies and inter–age-group contacts ranked highly for explaining IAV risk. Hygiene shortcomings (e.g., missing disinfection) and extensive cross-fostering (> 50% of litters) were also influential. Indicators of intensive production (larger herd size, weekly production intervals) were among the most relevant predictors. Notably, several predictors showed loading directions inconsistent with biological expectations; therefore, these results should be interpreted with caution. In addition, the predictors “caretaker enters stable with dedicated clothes only, ”the production-interval variables, “no boar contact possible in outside area,” and “quarantine area in separate building” showed high correlation (coefficients ≥ 0.70) with the presence of sneezing in fattening pigs, which is considered during interpretation.

**Table 8:** Predictors with highest variable importance in projection (VIP) scores in the PLS-DA model for the husbandry domain

Predictors in the husbandry domain (VIP > 1.5)	VIP	Loading	Direction
Contact with birds in the stable	3.37	0.31	Risk
*Insemination room in separate building*	3.23	0.30	Risk
Insemination room in separate other room	2.15	-0.20	Protective
Three weeks restocking or birth interval between batches	2.04	0.19	Risk
*Weaners stable not cleaned between batches*	2.04	-0.19	Protective
Number of suckling piglets	1.95	0.18	Risk
*Caretaker enters stable with dedicated clothes only*	1.86	0.17	Risk
*No bird nests in stable*	1.84	0.17	Risk
Farrowing compartment in separate room	1.79	0.17	Risk
Cross fostering in > 50% of litters	1.78	0.17	Risk
*Nursery in separate room*	1.70	0.16	Risk
*Fattening pigs in room with other age group*	1.68	-0.16	Protective
Two weeks restocking or birth interval between batches	1.59	-0.15	Protective
*No poultry herd closer than 1000 m*	1.57	0.15	Risk
*Farrowing compartment with other age group*	1.52	-0.14	Protective
*No boar contact possible in outside area*	1.46	0.14	Risk
Number weaners	1.42	0.13	Risk
*Quarantine area in separate building*	1.35	0.13	Risk
*Shipment area cleaned*	1.29	0.12	Risk
*No cattle kept on same farm*	1.28	0.12	Risk
*Poultry contact in outside area*	1.27	-0.12	Protective
Herd size	1.26	0.12	Risk
Stable clothes washed less than weekly	1.24	0.12	Risk
*All in all out in insemination room*	1.24	0.11	Risk
Other pig herd closer than 1000 m	1.21	0.11	Risk
Disinfection in weaners stable done annually	1.12	-0.10	Protective
Weekly restocking or birth interval between batches	1.10	0.10	Risk
*Gestation in separate room*	1.10	0.10	Risk
*All in all out in farrowing stable*	1.06	0.10	Risk
Separate caretaker per production unit	1.04	-0.10	Protective
Number of old sows	1.01	0.09	Risk

VIP scores, loadings, and their direction on the first component are shown. For conciseness, only predictors with VIP > 1.5 are displayed. Predictors with biologically questionable loading directions are in italics. Full results are available in Additional file 7

In the animal health domain (Table 9, for predictors with VIP > 1, for the full table refer to Additional file 7), history of respiratory disease reported in weaners remained the strongest predictor of IAV herd status; reports and examinations of acute influenza-like signs were also associated with increased risk, whereas observations of heavily coughing weaners and reports of dyspnea were associated with reduced risk. In the environmental domain, sampling during summer, higher airflows, and higher temperatures were protective. In the human health domain, summer sampling was also protective, whereas symptoms reported in caretakers were associated with increased risk of detecting IAV.

**Table 9:** Predictors with highest variable importance in projection (VIP) scores in the PLS-DA model for the animal health domain

Predictors in animal health domain (VIP > 1)	VIP	Loading	Direction
*Sneezing litters in farrowing compartment*	2.28	-0.32	Protective
*Farrowing rate ≥ 90%*	1.95	0.28	Risk
Reported disease in weaners	1.88	0.27	Risk
Nasal discharge reported in pigs	1.76	0.25	Risk
Dyspnea in pigs reported	1.67	-0.24	Protective
Missingness farrowing rate	1.49	-0.21	Protective
*Less than 28 piglets/per/year*	1.46	-0.21	Protective
Missingness history of respiratory disease	1.43	0.20	Risk
*Return to estrus rate ≤ 12%*	1.37	0.19	Risk
*Report of recent respiratory disease limited to few pigs*	1.32	0.19	Risk
Maximum rectal body temperatures 40.5–41.0 °C	1.26	0.18	Risk
Outbreak reported since study examination	1.26	0.18	Risk
Reported < 6 months since onset of last respiratory disease outbreak	1.26	0.18	Risk
Coughing index in weaners > 5%/min	1.25	-0.18	Protective
*No reported disease in fattening pigs*	1.22	0.17	Risk
*Sneezing reported in pigs*	1.10	-0.16	Protective
*Reported > 6 months since onset of last respiratory disease outbreak*	1.10	0.16	Risk
Sneezing index in weaners 3–10%/min	1.02	0.14	Risk
Missingness sneezing index in fattening pigs	1.01	0.14	Risk

VIP scores, loadings, and their direction on the first component are shown. For conciseness, only predictors with VIP > 1 are displayed. Predictors with biologically questionable loading directions are in italics. Full results are available in Additional file 7

### Key husbandry predictors assessed by logistic regression

**Fig. 4:**
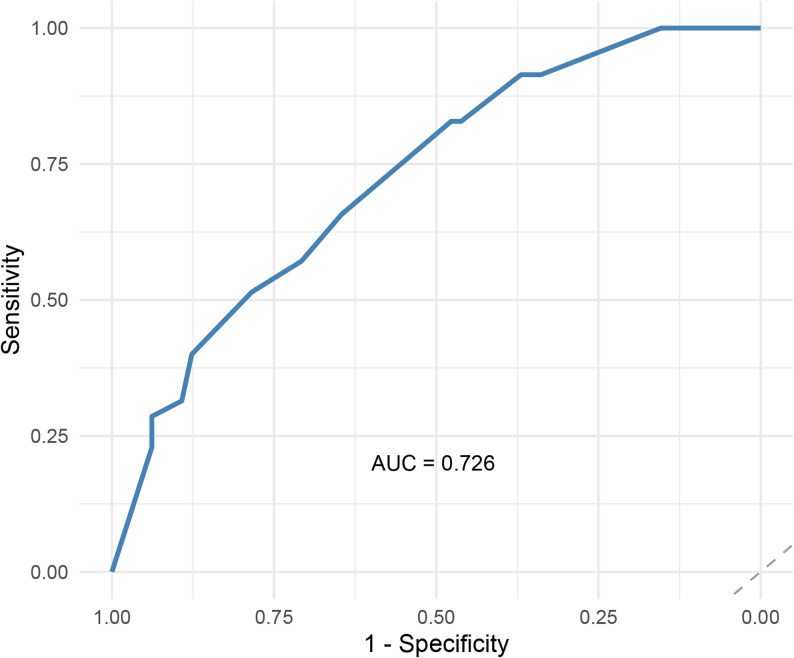
Receiver operating characteristic (ROC) plot with area under the curve (AUC) for the logistic regression model of the husbandry domain. ROC curves display 1 – specificity (false positive rate) on the x-axis and sensitivity (true positive rate) on the y-axis. The AUC is shown numerically within the plot

To illustrate the importance of the most influential predictors within the husbandry domain, the four predictors with the highest variable importance in projection (VIP) scores and biological plausibility (see Additional file 8 for details on predictor selection) - ”Contact with birds in the stable”, “Cross fostering practiced: >50% of litters”, ”Mixed age groups in nursery” and “Other pig herd closer than 1000 m” - were included in a multivariable logistic regression model. The model yielded an area under the ROC curve (AUC) of 0.73 (Fig. 4). The Hosmer-Lemeshow test had a p-value > 0.05, and McFadden’s pseudo-R² was 0.121. All predictors had variable inflation factors ranging from 1.06 to 1.12. Odds ratios and confidence intervals for each predictor are shown in Table 10; Wald z p-values are indicated for interpretability, but no claims of statistical significance are made as this was an exploratory analysis. For details, refer to Additional file 8.

**Table 10:** Logistic regression of husbandry predictors for IAV detection

Predictor	Estimate	SE	Odds ratio	95% CI	*P*>|z|	*N*
(Intercept)	-2.174	0.588	0.114	[0.032, 0.332]	< 0.001	100
Contact with birds in the stable	1.506	0.479	4.51	[1.806, 11.99]	< 0.01	100
Cross fostering practiced: >50% of litters	0.26	0.554	1.297	[0.429, 3.842]	0.64	100
Mixed age groups in nursery	0.868	0.486	2.382	[0.938, 6.4]	0.07	100
Other pig herd closer than 1000 m	0.529	0.514	1.698	[0.632, 4.83]	0.30	100

Reported are point estimates (β) with standard errors (SE), odds ratios (OR = exp(β)) with 95% Wald confidence intervals, two-sided Wald z-test p-values, and sample size (N) for each component. Given the exploratory nature of this model, p-values are provided for reference only and are not evidence of statistical significance

## Discussion

A cross-sectional study assessed pig herd-level occurrence of influenza A virus (IAV) and potential risk factors for infection in Switzerland, revealing that many IAV-positive herds did not display clinical signs. Overall, 35% (35/100) of herds tested positive for IAV, and 34.1% (29/85) of herds without observed clinical signs were IAV-positive. Trends toward increased IAV detection were observed in herds with bird contact, intensive cross-fostering practices, proximity to other pig herds, and insufficient age group separation; however, none of these associations reached statistical significance.

This exploratory cross-sectional study had several strengths that provided comprehensive insight into the largely unexplored epidemiology of IAV in Swiss pig herds. By including 100 herds, the study established an epidemiological profile of both clinically affected and inapparent cases. The questionnaire was carefully designed to capture all relevant exposures potentially associated with IAV detection, allowing a structured assessment of potential risk factors. Furthermore, the absence of restrictive inclusion and exclusion criteria enabled the study to include a wide variety of production types. In addition, the sampling strategy was designed to be sensitive to detect IAV infection at the herd level.

This study has several limitations that should be considered when interpreting the findings. The dataset was heterogeneous, combining data from different herd types and age groups, resulting in a considerable proportion of non-random missing data. The relatively small sample size limited the power to detect anything other than strong associations. Therefore, due to the exploratory nature of the analyses, potential confounders and mediators were not systematically adjusted for. To reduce the risk of spurious findings, false discovery rate (FDR)–adjusted p-values were calculated. To account for collinearity among predictors, high correlations (coefficients ≥ 0.70) were flagged and considered for interpretation. Consequently, no predictor reached the significance threshold (*p* < 0.05). Accordingly, the identified risk factors should be regarded as exploratory and require confirmation in independent studies. The results of the exploratory PLS-DA should also be interpreted with caution, as underlying confounding and bias in the dataset likely influenced the observed patterns.

Furthermore, information bias may have affected the results. The questionnaire was only marginally validated in three farms; data were not double-entered or cross-checked by independent examiners, and herd manager responses may have been influenced by recall limitations or reluctance to disclose shortcomings. Finally, the definition of IAV-positive herds was based solely on qPCR detection in nasal swabs and sampling was done at a single time point. Therefore, the analyses reflect associations with IAV detection rather than confirmed infection status and IAV detection does not allow any assessment of long-term IAV circulation.

In this study, the overall herd-level IAV detection rate was 35%, with a mean intra-herd detection rate of 8.5%. These values - particularly at the sample level - are lower than those reported in a recent European-wide study, which detected IAV in 56.6% of herds and 30.5% of samples [4]. When restricting the comparison to herds with clinical respiratory signs, 40% tested IAV-positive with a mean intra-herd detection rate of 18.0%, which remains lower than European estimates; however, the wide confidence intervals indicate statistical compatibility. Among subclinical herds, 34.1% tested positive, with a lower mean intra-herd detection rate of 6.8% (vs. 18.0% in herds with respiratory signs) and a lower viral load (Cp value = 35 vs. 33) (Table 2). The mean intra-herd IAV detection rate in subclinical herds was comparable to that reported in an active surveillance study in the United States, which identified IAV in 4.6% of samples [13]. However, these differences between herds with and without clinical signs were not statistically significant, suggesting that factors other than IAV detection contribute to the occurrence of respiratory signs. IAV detection in this study was unlikely to indicate the start of a clinical outbreak, as only one IAV-positive herd (3%) showed clinical signs within three months, and most herds (78%) had no recent respiratory history. This suggests that subclinical, largely unnoticed IAV circulation occurs at the herd level in Switzerland, in contrast to recurring IAV infections in specific age groups within endemically infected herds, which may intermittently develop clinical signs [14, 16, 17]. Pre-existing immunity and/or the absence of other major respiratory pathogens likely facilitate such subclinical circulation. As IAV vaccination is uncommon in Switzerland, additional host-, pathogen-, and environment-related factors probably modulate whether infection becomes clinically apparent. Overall, these findings underscore the need to better characterize pre-existing IAV immunity and to refine our understanding of the role of IAV within the PRDC in Swiss pig herds.

Husbandry in Swiss pig herds occurs against a background of limited exposure to several major pathogens and relative geographic isolation from other countries. However, biosecurity and hygiene measures are generally below European standards [28]. Herd sizes are smaller than those in other European countries, and pig farms are often located close to other pig farms (< 1 km) and commonly keep other livestock. Quarantine procedures for newly purchased animals are less formalized [46–48]. Notably, 75% of herds had an outdoor area, in contrast to reports from other European studies that found no farms with outdoor access. Contact with birds and wild boars was common, unlike in neighboring countries. Caretaker hygiene awareness was generally lower compared to European neighbors, where hygiene locks [49] and dedicated stable clothing [47, 48] are standard practice. Conversely, Swiss herds had fewer visitors [47]. Although visitors were usually provided with clothing, adherence to hygiene and downtime protocols after prior pig contact was below European standards. All-in-all-out (AIAO) management was generally applied room-wise at rates similar to other countries, however it was less frequently implemented in fattening units. Cleaning and stable downtime between batches were also comparable, but disinfection was rarely practiced, unlike in reference studies [49].

Despite the distinctive features of Swiss pig husbandry, statistical trends suggest that biosecurity practices and intensified production workflows influence IAV detection in herds. Larger herd sizes were associated with increased IAV detection, including subclinical infections [19, 23], likely due to intensified production practices, such as more frequent introductions of new pigs, facilitating the endemic persistence of IAV [50]. Production type had no significant effect on IAV detection, indicating that IAV circulates at a similar baseline level across Swiss pig herds, possibly because lower biosecurity standards allow relatively unrestricted viral transmission. Some predictors showed counterintuitive associations, reflecting correlations with herd size and intensified production. For example, mixed-age groups in the insemination room were linked to reduced risk, as this practice is more common in smaller herds with lower IAV detection; similar patterns were observed for several other predictors in PLS-DA.

Four key husbandry practices were analyzed together in the logistic regression model. Bird contact within stables, although unlikely to transmit IAV directly, may serve as a proxy for interactions with other animal species and potential biosecurity breaches, consistent with previous findings [19]. Intensive cross-fostering increased risk, aligning with previous reports [19]. Piglets can only receive immune cells from their biological mother if not cross-fostered after birth, whereas passive antibody transfer can occur from any sow, as described for *Mycoplasma hyopneumoniae* [51]. Early cross-fostering may therefore reduce cellular immunity. Alternatively, intensive cross-fostering could enhance contact between litters, facilitating IAV spread within the herd. The presence of mixed-age groups in the nursery appears plausible for increasing the risk of IAV detection, as inter-age-group contacts through moving pigs between compartments or through missing all-in-all-out flow have been confirmed as IAV risk factors in farrow-to-finish herds [25]. Proximity to other pig herds has also been described as a major risk factor for IAV detection [25], potentially increasing contact between pig herds and facilitating aerosol-borne transmission, particularly under low-biosecurity [25] conditions in Swiss pig herds [52]. Collectively, these four factors accounted for a substantial portion of IAV risk in the logistic regression model (AUC = 0.73), whereas the full husbandry PLS-DA model achieved an AUC of 0.79. Overall, the key husbandry factors influencing IAV risk in Swiss herds appear consistent with those reported in neighboring European countries.

Animal health indicators showed trends toward higher IAV detection in herds with acute respiratory disease, although associations were not statistically significant (Tables 6 and 9). Coughing and sneezing indices best distinguished herds with respiratory disease from those that were clinically healthy. Rectal body temperature was generally a poor indicator of respiratory disease; however, higher maximum rectal body temperatures and elevated sneezing indices were associated with an increased likelihood of IAV detection. IAV likelihood also appeared to be increased when herd managers reported affected weaners, observed nasal discharge, or reported a respiratory outbreak in the months before or after the study examination. In contrast, more chronic signs, such as dyspnea or very high coughing indices, were associated with reduced IAV detection.

These patterns are biologically plausible and were likely influenced by the sampling strategy and infection dynamics. IAV is most readily detected by nasal swabs during the acute phase of infection, which is typically characterized by fever and apathy, whereas during more chronic disease phases, when coughing or dyspnea predominate, the virus is mostly located deeper in the respiratory tract, and nasal swabs are less sensitive [1]. In contrast to findings from endemically infected herds in neighboring countries, where peaks of infection were observed at the beginning and end of the nursery period, the age of sampled weaners did not significantly affect IAV risk in this study. However, the limited sample size precludes firm conclusions regarding potential differences in detection rates between age groups. Overall, these results suggest that while acute respiratory signs can be associated with IAV detection, circulation dynamics in Swiss pig herds likely differ from those in neighboring European countries due to smaller herd sizes and the resulting lower likelihood of endemic persistence at the farm level following infection.

IAV detection in this study showed indirect indications of seasonal variation (Table 7). During summer, higher ambient temperatures (> 20 °C) were common, potentially confounding the observed associations between IAV detection and these environmental factors. While other studies highlight that in intensive pig production systems, IAV can circulate endemically throughout the year [4, 16], seasonal outbreak patterns have also been reported [1]. In Switzerland, this seasonal pattern may dominate, likely due to the predominance of small-scale production systems.

The observed seasonal pattern, with higher IAV detection and more outbreaks outside summer, may have confounded associations between IAV detection in pigs and symptom reports from herd managers. Winter climate conditions may have caused independent respiratory disease outbreaks affecting both pigs and herd managers at the same time.

## Conclusion

Influenza A virus (IAV) regularly occurs in Swiss pig herds without causing clinical signs. None of the potential IAV risk factors reached statistical significance. Nevertheless, the characteristics of study herds differed from those reported in other countries: pig production was more small-scale, less professionalized, and biosecurity and hygiene often fell below European standards. Despite this, trends in associations with risk factors were comparable to those found in other European studies. Given the zoonotic risk and the potential impact on pig health and production from the often inapparent circulation of IAV, implementing control measures on Swiss pig farms is warranted. Future studies should longitudinally monitor IAV circulation within herds and aim to confirm the risk factor candidates identified in this study.

## Supplementary Information

Below is the link to the electronic supplementary material.

Supplementary Material 1: Additional file 1. Description of data: Questionnaire and clinical examination checklist used for the epidemiological characterization of study pig herds

Supplementary Material 2: Additional file 2. Description of data: Codebook for dataset of pig herds included in the study, as well as codebook for continuous variables that were transformed to categorical for the PLS – DA

Supplementary Material 3: Additional file 3. Description of data: Descriptive data of predictors, outcome variable and meta-variables included in the current study

Supplementary Material 4: Additional file 4. Description of data: Sample ID, corresponding Farm ID, corresponding rectal body temperature of sampled pig, Cp-value of pan-IAV RT-qPCR and resulting classification as positive or negative are presented

Supplementary Material 5: Additional file 5. Description of data: Eight tables that contain associations and correlations of all predictors in the husbandry, animal health, environment and human health domain

Supplementary Material 6: Additional file 6. Description of data: Excel workbook with four sheets covering all predictors. Coefficients: Pairwise correlation coefficients; cells with correlation ≥ 0.70 are highlighted in red. P-values: Two-sided p-values for each pair; cells with *p* < 0.05 are highlighted in green. Sample sizes: Pairwise complete-case counts (N) used to compute each association. Methods: The method used for each pair. Method selection rules: Numeric vs. numeric: Pearson correlation. Any pair involving a binary variable (logical, two-level factor, or 0/1 numeric): Spearman (rank-based) by default. Categorical vs. categorical (> 2 levels): Cramér’s V (p-value from chi-squared test). Numeric vs. multi-level categorical: Correlation ratio (eta) with p-value from one-way ANOVA (F-test)

Supplementary Material 7: Additional file 7. Description of data: Domain wise results of PLS-DA analyses presented with sample scores plot, RMSEP plot, risk and protective factors table with loadings on component 1 and 2 of the model, Variable Importance in Projection (VIP) scores table and model performance summary

Supplementary Material 8: Additional file 8. Description of data: Detailed results of the logistic regression of key factors within the husbandry subset are presented along with predictor selection, model coefficients, odds ratios and confidence intervals and model diagnostics with assessment of multicollinearity, outlier assessment and calibration

## Data Availability

Supplementary material is available as Additional files. The dataset supporting the conclusions of this article and the code used for the statistical analysis can be found in the Git Hub repository https://github.com/jonasalexandersteiner/IAV_herds_presumably_non_diseased.
