# Supplementary material for: Epidemiology of Influenza A virus in Swiss pig herds: subclinical circulation and associated risk factors
Source: Porcine Health Manag. 2026 Apr 25;12:34. doi: 10.1186/s40813-026-00513-5 (PMC13261839; doi:10.1186/s40813-026-00513-5)
Supplement: Supplementary file 1 — Supplementary Material 1: Additional file 1. Description of data: Questionnaire and clinical examination checklist used for the epidemiological characterization of study pig herds [file 40813_2026_513_MOESM1_ESM.docx]

# Fragebogen Epidemiologie Influenza-A Viren in Schweinebeständen

## Allgemeine Informationen

- 1. Tierbesitzer: ID, Name, Adresse des Stalles, Telefon, E-Mail, TVD-Nummer, Seuchenfreiheit
  2. Zuordnung zur Proben ID Nummer, Entnahmedatum (dd/mmm/yyyy), symptomatisch (nach Meldung) /asymptomatisch (random)

## Herde

- 1. Haben Sie weitere betriebseigene Tierhaltungen oder Hobbytierhaltungen? Nein, Schweine, Hühner, Puten, anderes Geflügel, andere Tiere
  2. Sind betriebsfremde Tierhaltungen/ Tierkörperannahmestellen/Schlachthöfe von Schweinen in der Nähe (unter 1km Luftlinie)? (ja/nein/unbekannt)
  3. Sind betriebsfremde Tierhaltungen/ Tierkörperannahmestelle/Schlachthöfe von Geflügel in der Nähe (unter 1km Luftlinie)? (ja/nein/unbekannt)
  4. Welche(n) Produktionstyp(en) schliesst ihr Schweinebestand ein? Mast-Ferkelerzeuger, Mast-Ferkelaufzucht, Mastbestand / Zucht-Mast-Bestand, Zuchtbestand mit angeschlossener Teilmast/ AFP-Deckbestand, AFP-Deck-Wartebestand, AFP-Abferkelbestand, Kernzuchtbestand, Vermehrerbestand
  5. Hat ihr Schweinebestand einen SGD oder QGS-Status? (keinen Status/ I / A-Provisorisch/A /A-R /Nein/)
  6. Wie viele Tiere umfasste ihr Schweinbestand durchschnittlich in den letzten 6 Monaten?

Anzahl Saugferkel

Anzahl Absetzferkel (Jager) bis 10 Wochen

Anzahl Mastschweine ab 10 Woche

Anzahl Jungsauen (>180 & <250 LT)

Anzahl Altsauen

Anzahl Eber

- 1. Wie viele Tiere aus wie vielen externen Herkünften haben sie in den letzten 6 Monaten bezogen?

Anzahl Saugferkel

Anzahl Absetzferkel (Jager) bis 10 Wochen

Anzahl Mastschweine ab 10 Woche

Anzahl Jungsauen (>180 & <250 LT)

Anzahl Altsauen

Anzahl Eber

## Eingliederung einzelner Tiergruppen

3.1 Existiert ein Quarantänekonzept für zugekaufte Tiere vor Eingliederung in eine bestehende Tiergruppe? (ja/nein)

Wenn ja,

3.2 Bei welchen Tiergruppen? (ja/nein)

Saugferkel

Absetzferkel (Jager) bis 10 Wochen

Mastschweine ab 10 Woche

Jungsauen (>180 & <250 LT)

Altsauen

Eber

3.3 Wie viele Wochen dauert die Quarantäne? (bis 2, bis 3, mehr als 3)

3.4 Kommt es vor, dass andere Schweine ihres Bestandes direkten Kontakt mit den Tieren in Quarantäne haben? (ja/nein)

3.5 Ist ihnen bekannt, dass ein Bestand eines Zukaufs in den letzten 6 Monaten von respiratorischen Erkrankungen betroffen war? (0,1,2,3)

3.6 Wenn ja, wurde Influenza diagnostiziert? (ja/nein)

## Stall

4.1 Welchen Produktionsrhythmus verwenden sie in ihrem Betrieb? (1 Woche, 2 Wochen, 3 Wochen, 4 Wochen)

4.2 Wie erfolgt die Belegung in den einzelnen Abteilungen?

(Rein-Raus (100%), Rein-Raus (80%), Rein-Raus (60%), Rein-Raus (40%), Rein-Raus (20%) / kontinuierlich)

1. % Deckzentrum

2. % Wartestall

3. % Abferkelstall

4. % Aufzuchtstall

5. % Maststall

4.3 Führen Sie Wurfausgleich regelmässig durch? (niemals, selten, bis 10% der Würfe, 10-50% der Würfe, mehr als 50% der Würfe)

4.4 Muss eine Altersgruppe beim Umtrieb innerhalb des Stalles den Bereich einer anderen Altersgruppe passieren? (ja/nein)

4.5 Hat der jeweilige Produktionsbereich einen Auslauf? (ja/nein)

1. Deckzentrum

2. Wartestall

3. Abferkelstall

4. Aufzuchtstall

5. Maststall

6. Eberstall

4.6 Ist Kontakt zu Wirtschaftsgeflügel im Auslauf möglich? (Niemals, indirekt über Luftraum, direkter Kontakt an Zaun)

4.7 Ist Kontakt zu Wildvögeln im Auslauf möglich? (Niemals, indirekt über Luftraum, direkter Kontakt an Zaun)

4.8 Besteht ansonsten die Möglichkeit zu Kontakt mit Geflügel im Stall? (niemals, indirekt über gleichen Luftraum, direkter Kontakt)

4.9 Besteht ansonsten die Möglichkeit zu Kontakt mit Wildschweinen im Auslauf? (ja/nein)

4.10 Werden respiratorisch erkrankte Tiere isoliert (Krankenbucht)? (anderes Gebäude, gleiches Gebäude aber getrennter Luftraum, gleiches Gebäude mit gleichem Luftraum)

# Reinigung und Desinfektion

5.1 Wie häufig werden die einzelnen Bereiche gereinigt? (nie/seltener als jährlich/ eher jährlich/ eher monatlich/nach oder vor jeder Nutzung)

1. Deckzentrum

2. Wartestall

3. Abferkelstall

4. Aufzuchtstall

5. Maststall

6. Quarantäne

5.4 Wie häufig werden die Bereiche desinfiziert? (nie/seltener als jährlich/ eher jährlich/ eher monatlich/ nach oder vor jeder Nutzung)

1. Deckzentrum

2. Wartestall

3. Abferkelstall

4. Aufzuchtstall

5. Maststall

6. Quarantäne

5.5 Trockenen die einzelnen Bereiche vor einer Neubelegung komplett? (ja/nein)

1. Deckzentrum

2. Wartestall

3. Abferkelstall

4. Aufzuchtstall

5. Maststall

6. Quarantäne

5.6 Wird das für den Transport von Tieren zum Bestand verwendete Transportmittel vor/nach dem Transport gereinigt und desinfiziert? (nie/seltener als jährlich/ eher jährlich/ eher monatlich, nach oder vor jeder Nutzung

5.7 Wird der Verladebereich gereinigt oder gereinigt und desinfiziert? (nie/seltener als jährlich/ eher jährlich/ eher monatlich nach oder vor jeder Nutzung)

# Versorgung der Schweine und Besuche

6.1 Wer versorgt die Schweine? (Betriebsleiter, Betriebsleiter & Familienangehörige, Betriebsleiter & Angestellte)

6.2 Gibt es für Betriebsangehörige Schutzkleidung, die nur im Bestand getragen wird? (nein, Gummistiefel, Overall & Gummistiefel, Overall & Gummistiefel & Maske)

6.3 Verbleiben die Schutzkleidung ausser zur eventuellen Reinigung im Betrieb? (nein, Gummistiefel, Overall & Gummistiefel, Overall & Gummistiefel & Maske)

6.4 Gibt es Schutzkleidung speziell pro Produktionsbereich? (nein, Gummistiefel, Overall & Gummistiefel, Overall & Gummistiefel & Maske)

6.5 Gibt es eine Zuteilung der Betriebsangehörigen an Altersgruppen? (ja/nein)

6.6 Wird bei der Arbeitsplanung unterschiedliche Keimbelastung der Bereiche berücksichtigt? (ja/ /nein)

6.7 Erfolgt der Zutritt nur mit Schutzkleidung? (ja/nein)

6.8 Wie ist das Verhalten bei leichten Erkältungen/Grippeerkrankungen? (Kein Zutritt und Vertretung, Masketragen, keine Massnahme)

6.9 Waschen Sie und Betriebsangehörige die Hände vor Stallzutritt oder tragen Handschuhe? (ja/nein)

6.10 Gibt es eine Stiefeldesinfektion? (vor dem Betrieb, Benutzung zwischen Produktionsbereichen, nein)

6.11 Haben Sie oder Betriebsangehörige Kontakt zu Schweinen fremder Schweinehaltungen? (1–3-mal pro Monat aber nicht am selben Tag, 1–3-mal pro Woche aber nicht am selben Tag, am selben Tag, unbekannt)

6.12 Haben Sie Kontakt zu Geflügel? (1–3-mal pro Monat, 1–3-mal pro Woche aber nicht am selben Tag, am selben Tag, unbekannt)

6.13 Wie häufig sind betriebsfremde Besucher im Stall? (mehrmals pro Woche, mehrmals pro Monat, seltener, nie)

6.14 Wie viele Besucher hatten Sie in den letzten 6 Monaten?

6.15 Wie viele Stunden waren Besucher in den letzten 6 Monaten aufsummiert im Stall?

6.16 Werden Besucher in einer Liste erfasst? (nie, lückenhaft, immer)

6.17 Tragen Sie die selbe Schutzkleidung wie Betriebsanghörige?

6.18 Waschen Sie die Hände vor Stallzutritt? (ja/nein)

6.19 Wie ist die Vorgabe für Besucher bei leichten Erkältungen/Grippeerkrankungen? (Kein Zutritt, Masketragen, keine Massnahme)

6.20 Haben Sie Kontakt zu Schweinen fremder Schweinehaltungen? (1-3 mal pro Monat aber nicht am selben Tag, 1-3 mal pro Woche aber nicht am selben Tag, am selben Tag)

6.21 Haben Sie Kontakt zu Geflügel? (1-3 mal pro Monat, 1-3 mal pro Woche aber nicht am selben Tag, am selben Tag)

6.22 Hatten Betriebsfremde in den letzten 10 Tagen Erkältungssymptome oder Grippesymptome? (ja/nein)

## Zuchtleistung (letzte 6 Monate im Verlauf)

7.1 Umrauschrate (im Referenzbereich oder besser, bis 10% negative Abweichung vom Referenzbereich, mehr als 10% negative Abweichung vom Referenzbereich)

7.2 Abferkelrate (im Referenzbereich oder besser, bis 10% negative Abweichung vom Referenzbereich, mehr als 10% negative Abweichung vom Referenzbereich)

7.3 geborene Ferkel/Sau+Jahr (im Referenzbereich oder besser, bis 10% negative Abweichung vom Referenzbereich, mehr als 10% negative Abweichung vom Referenzbereich)

7.4 Aborte/ Sau+Jahr (im Referenzbereich oder besser, bis 10% negative Abweichung vom Referenzbereich, mehr als 10% negative Abweichung vom Referenzbereich)

7.5 Saugferkelmortalität (im Referenzbereich oder besser, bis 10% negative Abweichung vom Referenzbereich, mehr als 10% negative Abweichung vom Referenzbereich)

## Leistungsdaten (letzte 6 Monate im Verlauf)

7.6 Aufzuchtferkel: Zuwachs g/Tag (im Referenzbereich oder besser, bis 10% negative Abweichung vom Referenzbereich, 10% oder mehr negative Abweichung vom Referenzbereich)

7.7 Mastschweine: Zuwachs g/Tag (im Referenzbereich oder besser, bis 10% negative Abweichung vom Referenzbereich, 10% oder mehr negative Abweichung vom Referenzbereich)

7.6 Aufzuchtferkel: Futterverwertung (kg Futter pro 1kg Zunahme) (im Referenzbereich oder besser, bis 10% negative Abweichung vom Referenzbereich, 10% oder mehr negative Abweichung vom Referenzbereich)

7.7 Mastschweine: Futterverwertung (kg Futter pro 1kg Zunahme) (im Referenzbereich oder besser, bis 10% negative Abweichung vom Referenzbereich, 10% oder mehr negative Abweichung vom Referenzbereich)

# Spezifische Anamnese Schwein (EBJ vorher erfragen)

8.1 Gab es Respiratorische Erkrankungen in der Vergangenheit? (aktuell bis letzte 10 Tage, letzter Monat, bis letzte 6 Monate, mehr als 6 Monate, unbekannt)

Wenn ja,

8.2 Wann? (Wintersaison, Sommersaison, keine Unterscheidung, unbekannt)

8.3 Wie oft?

8.5 Seit wie vielen Tagen besteht der aktuelle Ausbruch?

8.6 Wie war der Prozentsatz erkrankter Tiere je Alterskategorie?

1. % Saugferkelwürfe

2. % Absatzferkel

3. % Mastschweine

4. % Altsauen

5. % Jungsauen

6. % Eber

8.7 Wie war der Prozentsatz der an der Erkrankung verstorbenen Tiere je Alterskategorie?

1. % Saugferkelwürfe

2. % Absatzferkel

3. % Mastschweine

4. % Altsauen

5. % Jungsauen

6. % Eber

8.8 Welche Symptome?

Niesen

Husten

Nasenausfluss

Fieber

Reduzierte Futteraufnahme

Apathie

8.9 Wurde ein Tierarzt konsultiert? (ja/nein)

8.10 Wurde Influenza diagnostiziert? (ja/nein)

8.11 Haben Sie eine Sondergenehmigung zur Impfung gegen Influenza?

# Spezifische Anamnese Landwirt

8.12 Hatten Sie in den letzten 10 Tagen Erkältungsymptome oder Grippesymptome? (ja/nein)

8.13 Hatten enge Kontaktpersonen (inklusive Betriebsangehörige und Familie) in den letzten 10 Tagen Erkältungsymptome oder Grippesymptome? (ja/nein)

8.14 Seit wie vielen Tagen sind Sie und/oder Angehörige erkrankt?

8.15 Welche Symptome?

Schnupfen

Husten

Bronchitis

Pneumonie

Fieber

Kopfschmerzen

Muskelschmerzen

8.16 Wie stark waren/sind die Symptome ausgeprägt (keine Änderung der Aktivität, Einschränkung der Aktivitäten, Arbeitsausfall)?

8.17 Wurde ein Arzt konsultiert? (ja/nein)

8.18 Wurde Influenza diagnostiziert? (ja/nein)

8.19 Sind sie aktuell gegen saisonale Grippe geimpft (weniger als 1 Jahr)? (ja/nein)

8.20 Sind enge Kontaktpersonen (inklusive Betriebsangehörige und Familie) aktuell gegen saisonale Grippe geimpft (weniger als 1 Jahr) (ja/nein)

8.21 Haben sie Vorerkrankungen? (ja/nein)

8.22 Sind sie Raucher? (ja/nein)

8.23 Wurden/Werden sie aktuell antiviral behandelt? (ja/nein)

## Influenzaprojekt: Untersuchung des Bestandes

#### Allgemein

9.0.1 Wenn mehr als ein Produktionsbereich, gibt es eine Separation zwischen den einzelnen Abteilungen?

| **Bereich** | **anderes Gebäude** | **gleiches Gebäude aber getrennter Luftraum** | **gleiches Gebäude mit gleichem Luftraum** |
| --- | --- | --- | --- |
| Deckzentrum |  |  |  |
| Wartestall |  |  |  |
| Abferkelstall |  |  |  |
| Aufzuchtstall |  |  |  |
| Maststall |  |  |  |

9.0.2 Gibt es eine Separation von Tiergruppen innerhalb des gleichen Produktionsbereichs?

| **Bereich** | **anderes Gebäude** | **gleiches Gebäude aber getrennter Luftraum** | **gleiches Gebäude mit gleichem Luftraum** |
| --- | --- | --- | --- |
| Deckzentrum |  |  |  |
| Wartestall |  |  |  |
| Abferkelstall |  |  |  |
| Aufzuchtstall |  |  |  |
| Maststall |  |  |  |

9.0.3 Ist der Quarantänestall räumlich abgetrennt?

- anderes Gebäude
- gleiches Gebäude aber getrennter Luftraum
- gleiches Gebäude mit gleichem Luftraum

9.0.4 Gibt es Vogelnester im Stall?

- keine
- bis 5
- mehr als 5

9.0.5 (Validierung 4.5) Ist Kontakt zu Wirtschaftsgeflügel im Auslauf möglich?

- niemals
- indirekt über gleichen Luftraum
- direkter Kontakt

9.0.6 (Validierung 4.6) Ist Kontakt zu Wildvögeln im Auslauf möglich?

- niemals
- indirekt über gleichen Luftraum
- direkter Kontakt durch Einfliegen
- direkter Kontakt am Zaun

9.0.7 (Validierung 4.7) Besteht ansonsten die Möglichkeit zu Kontakt mit Geflügel im Stall?

- niemals
- indirekt über gleichen Luftraum
- direkter Kontakt durch Einfliegen
- direkter Kontakt am Zaun

9.8 (Validierung 4.8) Werden sichtbar respiratorisch erkrankte Tiere isoliert (Krankenbucht)?

- keiner
- anderes Gebäude
- gleiches Gebäude aber getrennter Luftraum
- gleiches Gebäude mit gleichem Luftraum

#### Abferkelabteil

9.1.1 Gesundheitszustand Sauen und ihrer Ferkel

| **Befund (%)** | **Sauen** | | | | | **Ferkel** | | | | |
| --- | --- | --- | --- | --- | --- | --- | --- | --- | --- | --- |
| Störung des Allgemeinbefindens |  | | | | |  | | | | |
| Niesen |  | | | | |  | | | | |
| Husten |  | | | | |  | | | | |
| Nasenausfluss |  | | | | |  | | | | |
| Rektaltemperatur |  |  |  |  |  |  |  |  |  |  |

**Einrichtung**

- - 1. Wie ist Abweichung Raumtemperatur in Nähe der Sau (8-15 Grad)?

9.1.3 Wie ist die Temperatur im Ferkelnest?

- Akzeptabel
- Ferkel liegen dicht gedrängt (zu kalt)
- Ferkel liegen am Rande des Ferkelnestes (zu warm)
  - 1. Luftzug in Tiernähe (Sau/ Ferkel) über 0,2 m/s?

9.1.5 Wie ist die subjektive Luftqualität?

O Gut O Mässig O Schlecht

#### Besamungsstall

9.2.1 Gesundheitszustand Sauen

| **Befund (%)** | **Sauen** | | | | |
| --- | --- | --- | --- | --- | --- |
| Störung des Allgemeinbefindens |  | | | | |
| Niesen |  | | | | |
| Husten |  | | | | |
| Nasenausfluss |  | | | | |
| Rektaltemperatur |  |  |  |  |  |

**Einrichtung**

9.2.2 Wie ist Abweichung Raumtemperatur in Nähe der Sau (8-15 Grad)?

- - 1. Luftzug in Tiernähe (Sau) über 0,2 m/s?
    2. Wie ist die subjektive Luftqualität?

O Gut O Mässig O Schlecht

#### Wartestall

9.3.1 Gesundheitszustand Sauen

| **Befund** | **Sauen** | | | | |
| --- | --- | --- | --- | --- | --- |
| Störung des Allgemeinbefindens |  | | | | |
| Niesen |  | | | | |
| Husten |  | | | | |
| Nasenausfluss |  | | | | |
| Rektaltemperatur |  |  |  |  |  |

**Einrichtung**

9.3.2 Wie hoch ist Belegung pro Quadratmeter ca?

9.3.3 Wie viel Tiere pro Tränkeinrichtung?

9.3.4 Wie ist Abweichung Raumtemperatur in Nähe der Sau (8-15 Grad)?

9.3.5 Luftzug in Tiernähe?

9.3.6 Wie ist die subjektive Luftqualität?

O Gut O Mässig O Schlecht

#### Ferkelaufzucht

9.4.1 Gesundheitszustand Ferkel

| **Befund** | **Ferkel (%)** | | | | | |
| --- | --- | --- | --- | --- | --- | --- |
| Störung des Allgemeinbefindens |  | | | | | |
| Niesindex (pro Minute) |  | | |  | | |
| Hustenindex (pro Minute) |  | | |  | | |
| Nasenausfluss |  | | | | | |
| Rektaltemperatur |  |  |  | |  |  |

**Einrichtung**

9.4.2 Wie hoch ist Belegung pro Quadratmeter ca?

9.4.3 Wie viel Tiere pro Tränkeinrichtung?

9.4.4 Wie viele Tiere pro Fressplatz?

9.4.5 Wie ist Abweichung Raumtemperatur in Nähe der Tiere (18-24 Grad)?

9.4.6 Luftzug in Tiernähe über 0,2 m/s?

9.4.7 Wie ist die subjektive Luftqualität?

O Gut O Mässig O Schlecht

#### Mastabteil

9.5.1 Gesundheitszustand Mastschweine

| **Befund** | **Mastschweine (%)** | | | | | |
| --- | --- | --- | --- | --- | --- | --- |
| Störung des Allgemeinbefindens |  | | | | | |
| Niesindex (pro Minute) |  | | |  | | |
| Hustenindex (pro Minute) |  | | |  | | |
| Nasenausfluss |  | | | | | |
| Rektaltemperatur |  |  |  | |  |  |

**Einrichtung**

9.5.2 Wie hoch ist Belegung pro Quadratmeter ca?

9.5.3 Wie viel Tiere pro Tränkeinrichtung?

9.5.4 Wie hoch ist die Fressplatzbreite pro Tier?

9.5. Wie ist Abweichung Raumtemperatur in Nähe der Tiere (Vormast 15-22 Grad, Ausmast 9-18 Grad)?

9.5.6 Luftzug in Tiernähe über 0,2 m/s?

9.5.8 Wie ist die subjektive Luftqualität?

O Gut O Mässig O Schlecht
