# Supplementary material for: Epidemiology of Influenza A virus in Swiss pig herds: subclinical circulation and associated risk factors
Source: Porcine Health Manag. 2026 Apr 25;12:34. doi: 10.1186/s40813-026-00513-5 (PMC13261839; doi:10.1186/s40813-026-00513-5)
Supplement: Supplementary file 3 — Supplementary Material 3: Additional file 3. Description of data: Descriptive data of predictors, outcome variable and meta-variables included in the current study [file 40813_2026_513_MOESM3_ESM.html]

Descriptive statics SIV\_farms


# Descriptive statics SIV\_farms

```
## Dataset dimensions: 100 rows x 186 columns
```

# Descriptive statistics

Below you’ll find a per-variable summary and relevant plots for the
SIV\_farms (`df3`) data set. Character variables are excluded
from this analysis.

---

Outcomes

## IAV\_positive

**Summary statistics:**

```
 Variable: IAV_positive 
 Class: logical
FALSE 65
TRUE 35
Mode: FALSE
Proportion of levels: FALSE 0.65, TRUE 0.35
NA count: 0
```

## total\_samples

**Summary statistics:**

```
 Variable: total_samples 
 Class: integer
Min.  9.00
1st Qu. 19.00
Median 19.00
Mean 18.49
3rd Qu. 19.00
Max. 19.00
Variance: 2.475
Shapiro-Wilk p-value: 0.000000000000000001131 (not normal)
NA count: 0
```

## positive\_pigs

**Summary statistics:**

```
 Variable: positive_pigs 
 Class: integer
Min.  0.00
1st Qu.  0.00
Median  0.00
Mean  1.59
3rd Qu.  1.00
Max. 19.00
Variance: 14.06
Shapiro-Wilk p-value: 0.00000000000000004751 (not normal)
NA count: 0
```

## percent\_positive\_pigs

**Summary statistics:**

```
 Variable: percent_positive_pigs 
 Class: numeric
Min.   0.000
1st Qu.   0.000
Median   0.000
Mean   8.485
3rd Qu.   5.263
Max. 100.000
Variance: 406.6
Shapiro-Wilk p-value: 0.00000000000000003892 (not normal)
NA count: 0
```

## min\_ct

**Summary statistics:**

```
 Variable: min_ct 
 Class: numeric
Min. 20.61
1st Qu. 31.24
Median 34.69
Mean 33.11
3rd Qu. 36.96
Max. 37.73
NA's 65
Variance: NA
Shapiro-Wilk p-value: 0.00006768 (not normal)
NA count: 65
```

## max\_ct

**Summary statistics:**

```
 Variable: max_ct 
 Class: numeric
Min. 29.21
1st Qu. 34.68
Median 35.14
Mean 35.64
3rd Qu. 37.34
Max. 37.73
NA's 65
Variance: NA
Shapiro-Wilk p-value: 0.0003468 (not normal)
NA count: 65
```

## mean\_ct

**Summary statistics:**

```
 Variable: mean_ct 
 Class: numeric
Min. 28.18
1st Qu. 33.68
Median 34.75
Mean 34.68
3rd Qu. 36.96
Max. 37.73
NA's 65
Variance: NA
Shapiro-Wilk p-value: 0.003306 (not normal)
NA count: 65
```

## sd\_ct

**Summary statistics:**

```
 Variable: sd_ct 
 Class: numeric
Min. 0.3458
1st Qu. 0.4751
Median 1.1683
Mean 1.8308
3rd Qu. 2.8151
Max. 5.1829
NA's 82
Variance: NA
Shapiro-Wilk p-value: 0.002632 (not normal)
NA count: 82
```

## season\_sampling

**Summary statistics:**

```
 Variable: season_sampling 
 Class: factor
winter 23
spring 32
summer 25
autumn 20
Mode: spring
Proportion of levels: winter 0.23, spring 0.32, summer 0.25, autumn 0.20
Levels: winter, spring, summer, autumn
NA count: 0
```

## canton\_factor

**Summary statistics:**

```
 Variable: canton_factor 
 Class: factor
North-West 6
Berne + Solothurn 42
East & Zurich 19
Romandy 14
Lucerne 19
Mode: Berne + Solothurn
Proportion of levels: North-West 0.06, Berne + Solothurn 0.42, East & Zurich 0.19, Romandy 0.14, Lucerne 0.19
Levels: North-West, Berne + Solothurn, East & Zurich, Romandy, Lucerne
NA count: 0
```

## herdsize

**Summary statistics:**

```
 Variable: herdsize 
 Class: integer
Min.   25
1st Qu.  462
Median  720
Mean  843
3rd Qu. 1114
Max. 4183
Variance: 344813
Shapiro-Wilk p-value: 0.000000007035 (not normal)
NA count: 0
```

## production\_type\_factor

**Summary statistics:**

```
 Variable: production_type_factor 
 Class: factor
1 11
2 25
3 41
4 14
5 9
Mode: 3
Proportion of levels: 1 0.11, 2 0.25, 3 0.41, 4 0.14, 5 0.09
Levels: 1, 2, 3, 4, 5
NA count: 0
```

## Farrowing\_on\_farm

**Summary statistics:**

```
 Variable: Farrowing_on_farm 
 Class: logical
FALSE 12
TRUE 88
Mode: TRUE
Proportion of levels: FALSE 0.12, TRUE 0.88
NA count: 0
```

## Isemination\_on\_farm

**Summary statistics:**

```
 Variable: Isemination_on_farm 
 Class: logical
FALSE 30
TRUE 70
Mode: TRUE
Proportion of levels: FALSE 0.30, TRUE 0.70
NA count: 0
```

## Gestation\_on\_farm

**Summary statistics:**

```
 Variable: Gestation_on_farm 
 Class: logical
FALSE 30
TRUE 70
Mode: TRUE
Proportion of levels: FALSE 0.30, TRUE 0.70
NA count: 0
```

## Weaners\_on\_farm

**Summary statistics:**

```
 Variable: Weaners_on_farm 
 Class: logical
FALSE 11
TRUE 89
Mode: TRUE
Proportion of levels: FALSE 0.11, TRUE 0.89
NA count: 0
```

## Fattening\_on\_farm

**Summary statistics:**

```
 Variable: Fattening_on_farm 
 Class: logical
FALSE 37
TRUE 63
Mode: TRUE
Proportion of levels: FALSE 0.37, TRUE 0.63
NA count: 0
```

## respiratory\_signs

**Summary statistics:**

```
 Variable: respiratory_signs 
 Class: logical
FALSE 85
TRUE 15
Mode: FALSE
Proportion of levels: FALSE 0.85, TRUE 0.15
NA count: 0
```

## rectal\_temperature\_max

**Summary statistics:**

```
 Variable: rectal_temperature_max 
 Class: numeric
Min. 39.20
1st Qu. 40.10
Median 40.30
Mean 40.40
3rd Qu. 40.65
Max. 41.80
NA's 1
Variance: NA
Shapiro-Wilk p-value: 0.004161 (not normal)
NA count: 1
```

## rectal\_temperature\_avg

**Summary statistics:**

```
 Variable: rectal_temperature_avg 
 Class: numeric
Min. 38.80
1st Qu. 39.50
Median 39.70
Mean 39.67
3rd Qu. 39.90
Max. 40.40
NA's 1
Variance: NA
Shapiro-Wilk p-value: 0.02114 (not normal)
NA count: 1
```

## horses\_closeby

**Summary statistics:**

```
 Variable: horses_closeby 
 Class: logical
FALSE 92
TRUE 8
Mode: FALSE
Proportion of levels: FALSE 0.92, TRUE 0.08
NA count: 0
```

## dogs\_closeby

**Summary statistics:**

```
 Variable: dogs_closeby 
 Class: logical
FALSE 55
TRUE 45
Mode: FALSE
Proportion of levels: FALSE 0.55, TRUE 0.45
NA count: 0
```

## chicken\_closeby

**Summary statistics:**

```
 Variable: chicken_closeby 
 Class: logical
FALSE 67
TRUE 33
Mode: FALSE
Proportion of levels: FALSE 0.67, TRUE 0.33
NA count: 0
```

## turkey\_closeby

**Summary statistics:**

```
 Variable: turkey_closeby 
 Class: logical
FALSE 98
TRUE 2
Mode: FALSE
Proportion of levels: FALSE 0.98, TRUE 0.02
NA count: 0
```

## cattle\_closeby

**Summary statistics:**

```
 Variable: cattle_closeby 
 Class: logical
FALSE 49
TRUE 51
Mode: TRUE
Proportion of levels: FALSE 0.49, TRUE 0.51
NA count: 0
```

## cats\_closeby

**Summary statistics:**

```
 Variable: cats_closeby 
 Class: logical
FALSE 35
TRUE 65
Mode: TRUE
Proportion of levels: FALSE 0.35, TRUE 0.65
NA count: 0
```

## proximity\_to\_other\_pig\_herd

**Summary statistics:**

```
 Variable: proximity_to_other_pig_herd 
 Class: logical
FALSE 33
TRUE 67
Mode: TRUE
Proportion of levels: FALSE 0.33, TRUE 0.67
NA count: 0
```

## proximity\_to\_other\_poultry\_herd

**Summary statistics:**

```
 Variable: proximity_to_other_poultry_herd 
 Class: logical
FALSE 23
TRUE 77
Mode: TRUE
Proportion of levels: FALSE 0.23, TRUE 0.77
NA count: 0
```

## number\_suckling\_piglets

**Summary statistics:**

```
 Variable: number_suckling_piglets 
 Class: integer
Min.   20.0
1st Qu.  150.0
Median  242.5
Mean  275.6
3rd Qu.  350.0
Max. 1400.0
NA's 12
Variance: NA
Shapiro-Wilk p-value: 0.000000003806 (not normal)
NA count: 12
```

## number\_weaners

**Summary statistics:**

```
 Variable: number_weaners 
 Class: integer
Min.   16.0
1st Qu.  200.0
Median  300.0
Mean  368.5
3rd Qu.  500.0
Max. 2200.0
NA's 11
Variance: NA
Shapiro-Wilk p-value: 0.0000000001275 (not normal)
NA count: 11
```

## number\_fattening\_pigs

**Summary statistics:**

```
 Variable: number_fattening_pigs 
 Class: integer
Min.    1
1st Qu.   20
Median  184
Mean  283
3rd Qu.  470
Max. 1400
NA's 37
Variance: NA
Shapiro-Wilk p-value: 0.000001303 (not normal)
NA count: 37
```

## number\_young\_sows

**Summary statistics:**

```
 Variable: number_young_sows 
 Class: integer
Min.   2.0
1st Qu.   7.0
Median  10.0
Mean  22.4
3rd Qu.  20.0
Max. 350.0
NA's 28
Variance: NA
Shapiro-Wilk p-value: 0.0000000000000004535 (not normal)
NA count: 28
```

## number\_old\_sows

**Summary statistics:**

```
 Variable: number_old_sows 
 Class: integer
Min.   5.0
1st Qu.  40.0
Median  75.0
Mean  87.4
3rd Qu. 116.2
Max. 530.0
NA's 12
Variance: NA
Shapiro-Wilk p-value: 0.0000000003521 (not normal)
NA count: 12
```

## number\_boars

**Summary statistics:**

```
 Variable: number_boars 
 Class: integer
Min. 1.0
1st Qu. 1.0
Median 2.0
Mean 1.8
3rd Qu. 2.0
Max. 4.0
NA's 30
Variance: NA
Shapiro-Wilk p-value: 0.00000002447 (not normal)
NA count: 30
```

## number\_of\_origins

**Summary statistics:**

```
 Variable: number_of_origins 
 Class: integer
Min. 1.000
1st Qu. 1.000
Median 2.000
Mean 1.667
3rd Qu. 2.000
Max. 4.000
NA's 13
Variance: NA
Shapiro-Wilk p-value: 0.00000000009154 (not normal)
NA count: 13
```

## quarantine\_concept

**Summary statistics:**

```
 Variable: quarantine_concept 
 Class: logical
FALSE 34
TRUE 66
Mode: TRUE
Proportion of levels: FALSE 0.34, TRUE 0.66
NA count: 0
```

## quarantine\_time

**Summary statistics:**

```
 Variable: quarantine_time 
 Class: factor
16 12
4 10
3 10
6 6
5 4
20 3
2 1
other 12
NA's 42
Mode: 16
Proportion of levels: 16 0.21, 4 0.17, 3 0.17, 6 0.10, 5 0.07, 20 0.05, 2 0.02, other 0.21
Levels: 16, 4, 3, 6, 5, 20, 2, other
NA count: 42
```

## quarantine\_in\_herd\_contact

**Summary statistics:**

```
 Variable: quarantine_in_herd_contact 
 Class: logical
FALSE 62
TRUE 38
Mode: FALSE
Proportion of levels: FALSE 0.62, TRUE 0.38
NA count: 0
```

## herds\_of\_origin\_respiratory\_symptoms

**Summary statistics:**

```
 Variable: herds_of_origin_respiratory_symptoms 
 Class: logical
FALSE 96
TRUE 4
Mode: FALSE
Proportion of levels: FALSE 0.96, TRUE 0.04
NA count: 0
```

## herds\_of\_origin\_influenza\_diagnosis

**Summary statistics:**

```
 Variable: herds_of_origin_influenza_diagnosis 
 Class: logical
FALSE 99
TRUE 1
Mode: FALSE
Proportion of levels: FALSE 0.99, TRUE 0.01
NA count: 0
```

## production\_cycle

**Summary statistics:**

```
 Variable: production_cycle 
 Class: factor
1 15
2 16
3 47
other 22
Mode: 3
Proportion of levels: 1 0.15, 2 0.16, 3 0.47, other 0.22
Levels: 1, 2, 3, other
NA count: 0
```

## mode\_stable\_occupation\_ai\_centre

**Summary statistics:**

```
 Variable: mode_stable_occupation_ai_centre 
 Class: logical
FALSE 13
TRUE 55
Mode: TRUE
Proportion of levels: FALSE 0.19, TRUE 0.81
NA count: 32
```

## mode\_stable\_occupation\_gestation\_stable

**Summary statistics:**

```
 Variable: mode_stable_occupation_gestation_stable 
 Class: logical
FALSE 10
TRUE 64
Mode: TRUE
Proportion of levels: FALSE 0.14, TRUE 0.86
NA count: 26
```

## mode\_stable\_occupation\_farrowing\_stable

**Summary statistics:**

```
 Variable: mode_stable_occupation_farrowing_stable 
 Class: logical
FALSE 24
TRUE 65
Mode: TRUE
Proportion of levels: FALSE 0.27, TRUE 0.73
NA count: 11
```

## cross\_fostering\_farrowing\_stable

**Summary statistics:**

```
 Variable: cross_fostering_farrowing_stable 
 Class: factor
1 19
2 48
3 22
NA's 11
Mode: 2
Proportion of levels: 1 0.21, 2 0.54, 3 0.25
Levels: 1, 2, 3
NA count: 11
```

## mode\_stable\_occupation\_weaner\_stable

**Summary statistics:**

```
 Variable: mode_stable_occupation_weaner_stable 
 Class: logical
FALSE 43
TRUE 48
Mode: TRUE
Proportion of levels: FALSE 0.47, TRUE 0.53
NA count: 9
```

## mode\_stable\_occupation\_fattening\_stable

**Summary statistics:**

```
 Variable: mode_stable_occupation_fattening_stable 
 Class: logical
FALSE 54
TRUE 9
Mode: FALSE
Proportion of levels: FALSE 0.86, TRUE 0.14
NA count: 37
```

## passing\_through\_other\_age\_group

**Summary statistics:**

```
 Variable: passing_through_other_age_group 
 Class: logical
FALSE 60
TRUE 40
Mode: FALSE
Proportion of levels: FALSE 0.60, TRUE 0.40
NA count: 0
```

## outside\_area

**Summary statistics:**

```
 Variable: outside_area 
 Class: logical
FALSE 25
TRUE 75
Mode: TRUE
Proportion of levels: FALSE 0.25, TRUE 0.75
NA count: 0
```

## outside\_area\_ai\_centre

**Summary statistics:**

```
 Variable: outside_area_ai_centre 
 Class: logical
FALSE 71
TRUE 29
Mode: FALSE
Proportion of levels: FALSE 0.71, TRUE 0.29
NA count: 0
```

## outside\_area\_gestation\_stable

**Summary statistics:**

```
 Variable: outside_area_gestation_stable 
 Class: logical
FALSE 43
TRUE 57
Mode: TRUE
Proportion of levels: FALSE 0.43, TRUE 0.57
NA count: 0
```

## outside\_area\_farrowing\_stable

**Summary statistics:**

```
 Variable: outside_area_farrowing_stable 
 Class: logical
FALSE 94
TRUE 6
Mode: FALSE
Proportion of levels: FALSE 0.94, TRUE 0.06
NA count: 0
```

## outside\_area\_weaner\_stable

**Summary statistics:**

```
 Variable: outside_area_weaner_stable 
 Class: logical
FALSE 90
TRUE 10
Mode: FALSE
Proportion of levels: FALSE 0.90, TRUE 0.10
NA count: 0
```

## outside\_area\_fattening\_stable

**Summary statistics:**

```
 Variable: outside_area_fattening_stable 
 Class: logical
FALSE 63
TRUE 37
Mode: FALSE
Proportion of levels: FALSE 0.63, TRUE 0.37
NA count: 0
```

## outside\_area\_contact\_poultry

**Summary statistics:**

```
 Variable: outside_area_contact_poultry 
 Class: logical
FALSE 93
TRUE 7
Mode: FALSE
Proportion of levels: FALSE 0.93, TRUE 0.07
NA count: 0
```

## outside\_area\_contact\_wild\_birds

**Summary statistics:**

```
 Variable: outside_area_contact_wild_birds 
 Class: logical
FALSE 31
TRUE 69
Mode: TRUE
Proportion of levels: FALSE 0.31, TRUE 0.69
NA count: 0
```

## outside\_area\_contact\_wild\_boars

**Summary statistics:**

```
 Variable: outside_area_contact_wild_boars 
 Class: logical
FALSE 45
TRUE 55
Mode: TRUE
Proportion of levels: FALSE 0.45, TRUE 0.55
NA count: 0
```

## contact\_bird\_in\_stable

**Summary statistics:**

```
 Variable: contact_bird_in_stable 
 Class: logical
FALSE 61
TRUE 39
Mode: FALSE
Proportion of levels: FALSE 0.61, TRUE 0.39
NA count: 0
```

## cleaning\_ai\_centre

**Summary statistics:**

```
 Variable: cleaning_ai_centre 
 Class: factor
1 18
2 40
3 11
NA's 31
Mode: 2
Proportion of levels: 1 0.26, 2 0.58, 3 0.16
Levels: 1, 2, 3
NA count: 31
```

## cleaning\_gestation\_stable

**Summary statistics:**

```
 Variable: cleaning_gestation_stable 
 Class: logical
FALSE 26
TRUE 49
Mode: TRUE
Proportion of levels: FALSE 0.35, TRUE 0.65
NA count: 25
```

## cleaning\_farrowing\_stable

**Summary statistics:**

```
 Variable: cleaning_farrowing_stable 
 Class: logical
FALSE 4
TRUE 85
Mode: TRUE
Proportion of levels: FALSE 0.04, TRUE 0.96
NA count: 11
```

## cleaning\_weaner\_stable

**Summary statistics:**

```
 Variable: cleaning_weaner_stable 
 Class: logical
FALSE 11
TRUE 79
Mode: TRUE
Proportion of levels: FALSE 0.12, TRUE 0.88
NA count: 10
```

## cleaning\_fattening\_stable

**Summary statistics:**

```
 Variable: cleaning_fattening_stable 
 Class: factor
1 13
2 36
3 14
NA's 37
Mode: 2
Proportion of levels: 1 0.21, 2 0.57, 3 0.22
Levels: 1, 2, 3
NA count: 37
```

## cleaning\_quarantine

**Summary statistics:**

```
 Variable: cleaning_quarantine 
 Class: factor
1 9
2 7
3 8
NA's 76
Mode: 1
Proportion of levels: 1 0.38, 2 0.29, 3 0.33
Levels: 1, 2, 3
NA count: 76
```

## disinfection\_ai\_centre

**Summary statistics:**

```
 Variable: disinfection_ai_centre 
 Class: logical
FALSE 54
TRUE 15
Mode: FALSE
Proportion of levels: FALSE 0.78, TRUE 0.22
NA count: 31
```

## disinfection\_gestation\_stable

**Summary statistics:**

```
 Variable: disinfection_gestation_stable 
 Class: logical
FALSE 62
TRUE 13
Mode: FALSE
Proportion of levels: FALSE 0.83, TRUE 0.17
NA count: 25
```

## disinfection\_farrowing\_stable

**Summary statistics:**

```
 Variable: disinfection_farrowing_stable 
 Class: factor
1 47
2 9
3 34
NA's 10
Mode: 1
Proportion of levels: 1 0.52, 2 0.10, 3 0.38
Levels: 1, 2, 3
NA count: 10
```

## disinfection\_weaner\_stable

**Summary statistics:**

```
 Variable: disinfection_weaner_stable 
 Class: factor
1 52
2 10
3 28
NA's 10
Mode: 1
Proportion of levels: 1 0.58, 2 0.11, 3 0.31
Levels: 1, 2, 3
NA count: 10
```

## disinfection\_fattening\_stable

**Summary statistics:**

```
 Variable: disinfection_fattening_stable 
 Class: logical
FALSE 48
TRUE 15
Mode: FALSE
Proportion of levels: FALSE 0.76, TRUE 0.24
NA count: 37
```

## disinfection\_quarantine

**Summary statistics:**

```
 Variable: disinfection_quarantine 
 Class: logical
FALSE 18
TRUE 6
Mode: FALSE
Proportion of levels: FALSE 0.75, TRUE 0.25
NA count: 76
```

## drying\_ai\_centre

**Summary statistics:**

```
 Variable: drying_ai_centre 
 Class: factor
1 28
2 29
3 12
NA's 31
Mode: 2
Proportion of levels: 1 0.41, 2 0.42, 3 0.17
Levels: 1, 2, 3
NA count: 31
```

## drying\_gestation\_stable

**Summary statistics:**

```
 Variable: drying_gestation_stable 
 Class: logical
FALSE 46
TRUE 29
Mode: FALSE
Proportion of levels: FALSE 0.61, TRUE 0.39
NA count: 25
```

## drying\_farrowing\_stable

**Summary statistics:**

```
 Variable: drying_farrowing_stable 
 Class: logical
FALSE 12
TRUE 77
Mode: TRUE
Proportion of levels: FALSE 0.13, TRUE 0.87
NA count: 11
```

## drying\_weaner\_stable

**Summary statistics:**

```
 Variable: drying_weaner_stable 
 Class: logical
FALSE 9
TRUE 81
Mode: TRUE
Proportion of levels: FALSE 0.10, TRUE 0.90
NA count: 10
```

## drying\_fattening\_stable

**Summary statistics:**

```
 Variable: drying_fattening_stable 
 Class: factor
1 22
2 27
3 13
NA's 38
Mode: 2
Proportion of levels: 1 0.35, 2 0.44, 3 0.21
Levels: 1, 2, 3
NA count: 38
```

## drying\_quarantine

**Summary statistics:**

```
 Variable: drying_quarantine 
 Class: logical
FALSE 13
TRUE 11
Mode: FALSE
Proportion of levels: FALSE 0.54, TRUE 0.46
NA count: 76
```

## cleaning\_disinfection\_transport\_vehicle

**Summary statistics:**

```
 Variable: cleaning_disinfection_transport_vehicle 
 Class: logical
FALSE 4
TRUE 77
Mode: TRUE
Proportion of levels: FALSE 0.05, TRUE 0.95
NA count: 19
```

## cleaning\_shipment\_area

**Summary statistics:**

```
 Variable: cleaning_shipment_area 
 Class: logical
FALSE 61
TRUE 35
Mode: FALSE
Proportion of levels: FALSE 0.64, TRUE 0.36
NA count: 4
```

## caretaker\_number

**Summary statistics:**

```
 Variable: caretaker_number 
 Class: integer
Min.  1.00
1st Qu.  2.00
Median  3.00
Mean  2.85
3rd Qu.  3.00
Max. 16.00
Variance: 2.876
Shapiro-Wilk p-value: 0.000000000000004927 (not normal)
NA count: 0
```

## caretaker\_ppe\_stable

**Summary statistics:**

```
 Variable: caretaker_ppe_stable 
 Class: logical
FALSE 35
TRUE 65
Mode: TRUE
Proportion of levels: FALSE 0.35, TRUE 0.65
NA count: 0
```

## caretaker\_ppe\_washing\_interval

**Summary statistics:**

```
 Variable: caretaker_ppe_washing_interval 
 Class: logical
FALSE 12
TRUE 88
Mode: TRUE
Proportion of levels: FALSE 0.12, TRUE 0.88
NA count: 0
```

## caretaker\_ppe\_per\_unit

**Summary statistics:**

```
 Variable: caretaker_ppe_per_unit 
 Class: logical
FALSE 94
TRUE 6
Mode: FALSE
Proportion of levels: FALSE 0.94, TRUE 0.06
NA count: 0
```

## caretaker\_per\_unit

**Summary statistics:**

```
 Variable: caretaker_per_unit 
 Class: logical
FALSE 79
TRUE 21
Mode: FALSE
Proportion of levels: FALSE 0.79, TRUE 0.21
NA count: 0
```

## caretaker\_work\_flow\_hygiene\_between\_units

**Summary statistics:**

```
 Variable: caretaker_work_flow_hygiene_between_units 
 Class: logical
FALSE 31
TRUE 69
Mode: TRUE
Proportion of levels: FALSE 0.31, TRUE 0.69
NA count: 0
```

## caretaker\_entry\_ppe\_only

**Summary statistics:**

```
 Variable: caretaker_entry_ppe_only 
 Class: logical
FALSE 49
TRUE 51
Mode: TRUE
Proportion of levels: FALSE 0.49, TRUE 0.51
NA count: 0
```

## caretaker\_disease\_management

**Summary statistics:**

```
 Variable: caretaker_disease_management 
 Class: logical
FALSE 87
TRUE 12
Mode: FALSE
Proportion of levels: FALSE 0.88, TRUE 0.12
NA count: 1
```

## caretaker\_hands\_washed\_before\_entry

**Summary statistics:**

```
 Variable: caretaker_hands_washed_before_entry 
 Class: logical
FALSE 55
TRUE 45
Mode: FALSE
Proportion of levels: FALSE 0.55, TRUE 0.45
NA count: 0
```

## caretaker\_boot\_disinfection

**Summary statistics:**

```
 Variable: caretaker_boot_disinfection 
 Class: logical
FALSE 78
TRUE 22
Mode: FALSE
Proportion of levels: FALSE 0.78, TRUE 0.22
NA count: 0
```

## caretaker\_contact\_other\_pigs

**Summary statistics:**

```
 Variable: caretaker_contact_other_pigs 
 Class: logical
FALSE 78
TRUE 7
Mode: FALSE
Proportion of levels: FALSE 0.92, TRUE 0.08
NA count: 15
```

## caretaker\_contact\_poultry

**Summary statistics:**

```
 Variable: caretaker_contact_poultry 
 Class: logical
FALSE 71
TRUE 8
Mode: FALSE
Proportion of levels: FALSE 0.90, TRUE 0.10
NA count: 21
```

## visitors\_cumulative\_contact\_hours

**Summary statistics:**

```
 Variable: visitors_cumulative_contact_hours 
 Class: numeric
Min.  1.00
1st Qu.  3.00
Median  6.00
Mean  7.03
3rd Qu.  9.00
Max. 22.00
Variance: 24.09
Shapiro-Wilk p-value: 0.000001059 (not normal)
NA count: 0
```

## visitors\_list

**Summary statistics:**

```
 Variable: visitors_list 
 Class: logical
FALSE 3
TRUE 97
Mode: TRUE
Proportion of levels: FALSE 0.03, TRUE 0.97
NA count: 0
```

## ppe\_visitors

**Summary statistics:**

```
 Variable: ppe_visitors 
 Class: logical
FALSE 5
TRUE 95
Mode: TRUE
Proportion of levels: FALSE 0.05, TRUE 0.95
NA count: 0
```

## visitors\_hands\_washed\_before\_entry

**Summary statistics:**

```
 Variable: visitors_hands_washed_before_entry 
 Class: logical
FALSE 50
TRUE 50
Mode: FALSE
Proportion of levels: FALSE 0.50, TRUE 0.50
NA count: 0
```

## visitors\_disease\_management

**Summary statistics:**

```
 Variable: visitors_disease_management 
 Class: logical
FALSE 20
TRUE 5
Mode: FALSE
Proportion of levels: FALSE 0.80, TRUE 0.20
NA count: 75
```

## visitors\_contact\_other\_pigs

**Summary statistics:**

```
 Variable: visitors_contact_other_pigs 
 Class: logical
FALSE 9
TRUE 67
Mode: TRUE
Proportion of levels: FALSE 0.12, TRUE 0.88
NA count: 24
```

## visitors\_respiratory\_symptoms

**Summary statistics:**

```
 Variable: visitors_respiratory_symptoms 
 Class: logical
FALSE 98
TRUE 2
Mode: FALSE
Proportion of levels: FALSE 0.98, TRUE 0.02
NA count: 0
```

## return\_to\_service\_rate

**Summary statistics:**

```
 Variable: return_to_service_rate 
 Class: logical
FALSE 14
TRUE 54
Mode: TRUE
Proportion of levels: FALSE 0.21, TRUE 0.79
NA count: 32
```

## farrowing\_rate

**Summary statistics:**

```
 Variable: farrowing_rate 
 Class: logical
FALSE 19
TRUE 62
Mode: TRUE
Proportion of levels: FALSE 0.23, TRUE 0.77
NA count: 19
```

## piglets\_per\_sow\_year

**Summary statistics:**

```
 Variable: piglets_per_sow_year 
 Class: logical
FALSE 8
TRUE 74
Mode: TRUE
Proportion of levels: FALSE 0.10, TRUE 0.90
NA count: 18
```

## abortions\_per\_sow\_year

**Summary statistics:**

```
 Variable: abortions_per_sow_year 
 Class: logical
FALSE 1
TRUE 81
Mode: TRUE
Proportion of levels: FALSE 0.01, TRUE 0.99
NA count: 18
```

## piglet\_mortality

**Summary statistics:**

```
 Variable: piglet_mortality 
 Class: logical
FALSE 15
TRUE 66
Mode: TRUE
Proportion of levels: FALSE 0.19, TRUE 0.81
NA count: 19
```

## feed\_conversion\_rate\_fattening\_pigs

**Summary statistics:**

```
 Variable: feed_conversion_rate_fattening_pigs 
 Class: logical
FALSE 3
TRUE 11
Mode: TRUE
Proportion of levels: FALSE 0.21, TRUE 0.79
NA count: 86
```

## respiratory\_history\_swine

**Summary statistics:**

```
 Variable: respiratory_history_swine 
 Class: factor
1 54
2 20
3 5
NA's 21
Mode: 1
Proportion of levels: 1 0.68, 2 0.25, 3 0.06
Levels: 1, 2, 3
NA count: 21
```

## time\_respiratory\_disease

**Summary statistics:**

```
 Variable: time_respiratory_disease 
 Class: logical
FALSE 6
TRUE 52
Mode: TRUE
Proportion of levels: FALSE 0.10, TRUE 0.90
NA count: 42
```

## frequency\_respi\_outbreak

**Summary statistics:**

```
 Variable: frequency_respi_outbreak 
 Class: factor
never 36
few pigs affected no outbreak 19
once 23
1x/year 11
2x/year 7
3x or more per year 4
Mode: never
Proportion of levels: never 0.36, few pigs affected no outbreak 0.19, once 0.23, 1x/year 0.11, 2x/year 0.07, 3x or more per year 0.04
Levels: never, few pigs affected no outbreak, once, 1x/year, 2x/year, 3x or more per year
NA count: 0
```

## outbreak\_since\_examination

**Summary statistics:**

```
 Variable: outbreak_since_examination 
 Class: logical
FALSE 95
TRUE 5
Mode: FALSE
Proportion of levels: FALSE 0.95, TRUE 0.05
NA count: 0
```

## suckling\_piglets\_diseased

**Summary statistics:**

```
 Variable: suckling_piglets_diseased 
 Class: logical
FALSE 94
TRUE 6
Mode: FALSE
Proportion of levels: FALSE 0.94, TRUE 0.06
NA count: 0
```

## weaners\_diseased

**Summary statistics:**

```
 Variable: weaners_diseased 
 Class: logical
FALSE 55
TRUE 45
Mode: FALSE
Proportion of levels: FALSE 0.55, TRUE 0.45
NA count: 0
```

## fattening\_pigs\_diseased

**Summary statistics:**

```
 Variable: fattening_pigs_diseased 
 Class: logical
FALSE 73
TRUE 27
Mode: FALSE
Proportion of levels: FALSE 0.73, TRUE 0.27
NA count: 0
```

## young\_sows\_diseased

**Summary statistics:**

```
 Variable: young_sows_diseased 
 Class: logical
FALSE 97
TRUE 3
Mode: FALSE
Proportion of levels: FALSE 0.97, TRUE 0.03
NA count: 0
```

## old\_sows\_diseased

**Summary statistics:**

```
 Variable: old_sows_diseased 
 Class: logical
FALSE 97
TRUE 3
Mode: FALSE
Proportion of levels: FALSE 0.97, TRUE 0.03
NA count: 0
```

## boars\_diseased

**Summary statistics:**

```
 Variable: boars_diseased 
 Class: logical
FALSE 99
TRUE 1
Mode: FALSE
Proportion of levels: FALSE 0.99, TRUE 0.01
NA count: 0
```

## symptom\_swine\_sneezing

**Summary statistics:**

```
 Variable: symptom_swine_sneezing 
 Class: logical
FALSE 80
TRUE 20
Mode: FALSE
Proportion of levels: FALSE 0.80, TRUE 0.20
NA count: 0
```

## symptom\_swine\_coughing

**Summary statistics:**

```
 Variable: symptom_swine_coughing 
 Class: logical
FALSE 37
TRUE 63
Mode: TRUE
Proportion of levels: FALSE 0.37, TRUE 0.63
NA count: 0
```

## symptom\_swine\_nasal\_discharge

**Summary statistics:**

```
 Variable: symptom_swine_nasal_discharge 
 Class: logical
FALSE 94
TRUE 6
Mode: FALSE
Proportion of levels: FALSE 0.94, TRUE 0.06
NA count: 0
```

## symptom\_swine\_fever

**Summary statistics:**

```
 Variable: symptom_swine_fever 
 Class: logical
FALSE 85
TRUE 15
Mode: FALSE
Proportion of levels: FALSE 0.85, TRUE 0.15
NA count: 0
```

## symptom\_swine\_feed\_intake\_red

**Summary statistics:**

```
 Variable: symptom_swine_feed_intake_red 
 Class: logical
FALSE 66
TRUE 34
Mode: FALSE
Proportion of levels: FALSE 0.66, TRUE 0.34
NA count: 0
```

## symptom\_swine\_apathy

**Summary statistics:**

```
 Variable: symptom_swine_apathy 
 Class: logical
FALSE 74
TRUE 26
Mode: FALSE
Proportion of levels: FALSE 0.74, TRUE 0.26
NA count: 0
```

## symptom\_swine\_dyspnoea

**Summary statistics:**

```
 Variable: symptom_swine_dyspnoea 
 Class: logical
FALSE 87
TRUE 13
Mode: FALSE
Proportion of levels: FALSE 0.87, TRUE 0.13
NA count: 0
```

## vet\_consultation

**Summary statistics:**

```
 Variable: vet_consultation 
 Class: logical
FALSE 79
TRUE 21
Mode: FALSE
Proportion of levels: FALSE 0.79, TRUE 0.21
NA count: 0
```

## influenza\_diagnosis

**Summary statistics:**

```
 Variable: influenza_diagnosis 
 Class: logical
FALSE 96
TRUE 4
Mode: FALSE
Proportion of levels: FALSE 0.96, TRUE 0.04
NA count: 0
```

## influenza\_vaccination

**Summary statistics:**

```
 Variable: influenza_vaccination 
 Class: logical
FALSE 98
TRUE 2
Mode: FALSE
Proportion of levels: FALSE 0.98, TRUE 0.02
NA count: 0
```

## respiratory\_history\_human

**Summary statistics:**

```
 Variable: respiratory_history_human 
 Class: logical
FALSE 88
TRUE 12
Mode: FALSE
Proportion of levels: FALSE 0.88, TRUE 0.12
NA count: 0
```

## respiratory\_history\_contact\_person

**Summary statistics:**

```
 Variable: respiratory_history_contact_person 
 Class: logical
FALSE 94
TRUE 6
Mode: FALSE
Proportion of levels: FALSE 0.94, TRUE 0.06
NA count: 0
```

## symptom\_human\_sneezing

**Summary statistics:**

```
 Variable: symptom_human_sneezing 
 Class: logical
FALSE 88
TRUE 12
Mode: FALSE
Proportion of levels: FALSE 0.88, TRUE 0.12
NA count: 0
```

## symptom\_human\_coughing

**Summary statistics:**

```
 Variable: symptom_human_coughing 
 Class: logical
FALSE 91
TRUE 9
Mode: FALSE
Proportion of levels: FALSE 0.91, TRUE 0.09
NA count: 0
```

## symptom\_human\_bronchitis

**Summary statistics:**

```
 Variable: symptom_human_bronchitis 
 Class: logical
FALSE 99
TRUE 1
Mode: FALSE
Proportion of levels: FALSE 0.99, TRUE 0.01
NA count: 0
```

## symptom\_human\_pneumonia

**Summary statistics:**

```
 Variable: symptom_human_pneumonia 
 Class: logical
FALSE 99
TRUE 1
Mode: FALSE
Proportion of levels: FALSE 0.99, TRUE 0.01
NA count: 0
```

## symptom\_human\_fever

**Summary statistics:**

```
 Variable: symptom_human_fever 
 Class: logical
FALSE 96
TRUE 4
Mode: FALSE
Proportion of levels: FALSE 0.96, TRUE 0.04
NA count: 0
```

## symptom\_human\_headache

**Summary statistics:**

```
 Variable: symptom_human_headache 
 Class: logical
FALSE 96
TRUE 4
Mode: FALSE
Proportion of levels: FALSE 0.96, TRUE 0.04
NA count: 0
```

## symptom\_human\_myalgia

**Summary statistics:**

```
 Variable: symptom_human_myalgia 
 Class: logical
FALSE 97
TRUE 3
Mode: FALSE
Proportion of levels: FALSE 0.97, TRUE 0.03
NA count: 0
```

## symptom\_severity

**Summary statistics:**

```
 Variable: symptom_severity 
 Class: logical
FALSE 8
TRUE 2
Mode: FALSE
Proportion of levels: FALSE 0.80, TRUE 0.20
NA count: 90
```

## physician\_consultation

**Summary statistics:**

```
 Variable: physician_consultation 
 Class: logical
FALSE 98
TRUE 2
Mode: FALSE
Proportion of levels: FALSE 0.98, TRUE 0.02
NA count: 0
```

## flu\_vaccination

**Summary statistics:**

```
 Variable: flu_vaccination 
 Class: logical
FALSE 93
TRUE 7
Mode: FALSE
Proportion of levels: FALSE 0.93, TRUE 0.07
NA count: 0
```

## flu\_vaccination\_contacts

**Summary statistics:**

```
 Variable: flu_vaccination_contacts 
 Class: logical
FALSE 88
TRUE 12
Mode: FALSE
Proportion of levels: FALSE 0.88, TRUE 0.12
NA count: 0
```

## chronic\_disease\_condition

**Summary statistics:**

```
 Variable: chronic_disease_condition 
 Class: logical
FALSE 83
TRUE 17
Mode: FALSE
Proportion of levels: FALSE 0.83, TRUE 0.17
NA count: 0
```

## smoker

**Summary statistics:**

```
 Variable: smoker 
 Class: logical
FALSE 73
TRUE 27
Mode: FALSE
Proportion of levels: FALSE 0.73, TRUE 0.27
NA count: 0
```

## Age\_weeks\_factor

**Summary statistics:**

```
 Variable: Age_weeks_factor 
 Class: factor
weaners_4â5 55
weaners_6â7 17
weaners_8â9 4
weaners_10â12 12
weaners_mixed_ages 12
Mode: weaners_4â5
Proportion of levels: weaners_4â5 0.55, weaners_6â7 0.17, weaners_8â9 0.04, weaners_10â12 0.12, weaners_mixed_ages 0.12
Levels: weaners_4â5, weaners_6â7, weaners_8â9, weaners_10â12, weaners_mixed_ages
NA count: 0
```

## separation\_quarantine\_area

**Summary statistics:**

```
 Variable: separation_quarantine_area 
 Class: factor
1 8
2 14
3 76
NA's 2
Mode: 3
Proportion of levels: 1 0.08, 2 0.14, 3 0.78
Levels: 1, 2, 3
NA count: 2
```

## bird\_nests

**Summary statistics:**

```
 Variable: bird_nests 
 Class: logical
FALSE 79
TRUE 21
Mode: FALSE
Proportion of levels: FALSE 0.79, TRUE 0.21
NA count: 0
```

## farrowing\_piglets\_reduced\_general\_wellbeing

**Summary statistics:**

```
 Variable: farrowing_piglets_reduced_general_wellbeing 
 Class: logical
FALSE 84
TRUE 1
Mode: FALSE
Proportion of levels: FALSE 0.99, TRUE 0.01
NA count: 15
```

## farrowing\_piglets\_sneezing

**Summary statistics:**

```
 Variable: farrowing_piglets_sneezing 
 Class: numeric
Min.  0.000
1st Qu.  0.000
Median  0.000
Mean  4.647
3rd Qu.  8.000
Max. 33.000
NA's 15
Variance: NA
Shapiro-Wilk p-value: 0.0000000000008049 (not normal)
NA count: 15
```

## farrowing\_piglets\_coughing

**Summary statistics:**

```
 Variable: farrowing_piglets_coughing 
 Class: numeric
Min.  0.0000
1st Qu.  0.0000
Median  0.0000
Mean  0.8353
3rd Qu.  0.0000
Max. 17.0000
NA's 15
Variance: NA
Shapiro-Wilk p-value: 0.000000000000000003459 (not normal)
NA count: 15
```

## farrowing\_room\_temperature

**Summary statistics:**

```
 Variable: farrowing_room_temperature 
 Class: numeric
Min. 12.80
1st Qu. 17.65
Median 19.30
Mean 19.75
3rd Qu. 21.43
Max. 30.20
NA's 28
Variance: NA
Shapiro-Wilk p-value: 0.1633 (normal)
NA count: 28
```

## farrowing\_nest\_temperature\_ok

**Summary statistics:**

```
 Variable: farrowing_nest_temperature_ok 
 Class: logical
FALSE 14
TRUE 65
Mode: TRUE
Proportion of levels: FALSE 0.18, TRUE 0.82
NA count: 21
```

## farrowing\_airflow

**Summary statistics:**

```
 Variable: farrowing_airflow 
 Class: numeric
Min. 0.0000
1st Qu. 0.1000
Median 0.2000
Mean 0.1988
3rd Qu. 0.2050
Max. 1.0000
NA's 32
Variance: NA
Shapiro-Wilk p-value: 0.00000002934 (not normal)
NA count: 32
```

## farrowing\_air\_quality

**Summary statistics:**

```
 Variable: farrowing_air_quality 
 Class: factor
1 14
2 61
3 11
NA's 14
Mode: 2
Proportion of levels: 1 0.16, 2 0.71, 3 0.13
Levels: 1, 2, 3
NA count: 14
```

## farrowing\_airspace\_with\_other\_agegroup

**Summary statistics:**

```
 Variable: farrowing_airspace_with_other_agegroup 
 Class: factor
1 30
2 43
3 12
NA's 15
Mode: 2
Proportion of levels: 1 0.35, 2 0.51, 3 0.14
Levels: 1, 2, 3
NA count: 15
```

## ai\_sows\_room\_temperature

**Summary statistics:**

```
 Variable: ai_sows_room_temperature 
 Class: numeric
Min.  8.70
1st Qu. 15.05
Median 18.00
Mean 18.53
3rd Qu. 21.02
Max. 30.40
NA's 54
Variance: NA
Shapiro-Wilk p-value: 0.6843 (normal)
NA count: 54
```

## ai\_sows\_airflow

**Summary statistics:**

```
 Variable: ai_sows_airflow 
 Class: numeric
Min. 0.0200
1st Qu. 0.1175
Median 0.2000
Mean 0.2211
3rd Qu. 0.3000
Max. 0.5000
NA's 56
Variance: NA
Shapiro-Wilk p-value: 0.01042 (not normal)
NA count: 56
```

## ai\_sows\_air\_quality

**Summary statistics:**

```
 Variable: ai_sows_air_quality 
 Class: factor
1 10
2 39
3 9
NA's 42
Mode: 2
Proportion of levels: 1 0.17, 2 0.67, 3 0.16
Levels: 1, 2, 3
NA count: 42
```

## ai\_airspace\_with\_other\_agegroup

**Summary statistics:**

```
 Variable: ai_airspace_with_other_agegroup 
 Class: factor
1 7
2 25
3 25
NA's 43
Mode: 2
Proportion of levels: 1 0.12, 2 0.44, 3 0.44
Levels: 1, 2, 3
NA count: 43
```

## gestation\_sows\_qm\_per\_animal

**Summary statistics:**

```
 Variable: gestation_sows_qm_per_animal 
 Class: numeric
Min. 1.970
1st Qu. 2.500
Median 3.210
Mean 3.701
3rd Qu. 4.440
Max. 8.870
NA's 27
Variance: NA
Shapiro-Wilk p-value: 0.0000005447 (not normal)
NA count: 27
```

## gestation\_sows\_animals\_per\_water\_source

**Summary statistics:**

```
 Variable: gestation_sows_animals_per_water_source 
 Class: numeric
Min.  0.330
1st Qu.  6.150
Median  8.400
Mean  9.368
3rd Qu. 12.000
Max. 45.000
NA's 27
Variance: NA
Shapiro-Wilk p-value: 0.0000000104 (not normal)
NA count: 27
```

## gestation\_sows\_room\_temperature

**Summary statistics:**

```
 Variable: gestation_sows_room_temperature 
 Class: numeric
Min.  4.80
1st Qu. 14.10
Median 18.30
Mean 18.13
3rd Qu. 20.70
Max. 32.40
NA's 39
Variance: NA
Shapiro-Wilk p-value: 0.464 (normal)
NA count: 39
```

## gestation\_sows\_airflow

**Summary statistics:**

```
 Variable: gestation_sows_airflow 
 Class: numeric
Min. 0.0000
1st Qu. 0.1300
Median 0.2000
Mean 0.2692
3rd Qu. 0.3000
Max. 1.5000
NA's 41
Variance: NA
Shapiro-Wilk p-value: 0.0000000001037 (not normal)
NA count: 41
```

## gestation\_sows\_air\_quality

**Summary statistics:**

```
 Variable: gestation_sows_air_quality 
 Class: factor
1 24
2 43
3 5
NA's 28
Mode: 2
Proportion of levels: 1 0.33, 2 0.60, 3 0.07
Levels: 1, 2, 3
NA count: 28
```

## gestation\_sows\_airspace\_with\_other\_agegroup

**Summary statistics:**

```
 Variable: gestation_sows_airspace_with_other_agegroup 
 Class: factor
1 21
2 28
3 23
NA's 28
Mode: 2
Proportion of levels: 1 0.29, 2 0.39, 3 0.32
Levels: 1, 2, 3
NA count: 28
```

## weaners\_reduced\_general\_wellbeing

**Summary statistics:**

```
 Variable: weaners_reduced_general_wellbeing 
 Class: logical
FALSE 93
TRUE 1
Mode: FALSE
Proportion of levels: FALSE 0.99, TRUE 0.01
NA count: 6
```

## weaners\_sneezing

**Summary statistics:**

```
 Variable: weaners_sneezing 
 Class: numeric
Min.  0.000
1st Qu.  0.500
Median  3.185
Mean  5.996
3rd Qu.  7.825
Max. 53.300
NA's 6
Variance: NA
Shapiro-Wilk p-value: 0.0000000000008523 (not normal)
NA count: 6
```

## weaners\_coughing

**Summary statistics:**

```
 Variable: weaners_coughing 
 Class: numeric
Min. 0.0000
1st Qu. 0.0000
Median 0.0000
Mean 0.8978
3rd Qu. 0.8350
Max. 7.6000
NA's 6
Variance: NA
Shapiro-Wilk p-value: 0.00000000000001055 (not normal)
NA count: 6
```

## weaners\_discharge

**Summary statistics:**

```
 Variable: weaners_discharge 
 Class: logical
FALSE 91
TRUE 3
Mode: FALSE
Proportion of levels: FALSE 0.97, TRUE 0.03
NA count: 6
```

## weaners\_qm\_per\_animal

**Summary statistics:**

```
 Variable: weaners_qm_per_animal 
 Class: numeric
Min. 0.1800
1st Qu. 0.3100
Median 0.3700
Mean 0.4066
3rd Qu. 0.4400
Max. 1.2300
NA's 6
Variance: NA
Shapiro-Wilk p-value: 0.0000000008641 (not normal)
NA count: 6
```

## weaners\_animals\_per\_feeding\_site\_factor

**Summary statistics:**

```
 Variable: weaners_animals_per_feeding_site_factor 
 Class: factor
<20 27
<30 21
<40 21
>40 17
trough 8
NA's 6
Mode: <20
Proportion of levels: <20 0.29, <30 0.22, <40 0.22, >40 0.18, trough 0.09
Levels: <20, <30, <40, >40, trough
NA count: 6
```

## weaners\_animals\_per\_water\_source

**Summary statistics:**

```
 Variable: weaners_animals_per_water_source 
 Class: numeric
Min.  6.000
1st Qu.  9.762
Median 11.850
Mean 14.886
3rd Qu. 18.375
Max. 50.000
NA's 6
Variance: NA
Shapiro-Wilk p-value: 0.0000000001342 (not normal)
NA count: 6
```

## weaners\_room\_temperature

**Summary statistics:**

```
 Variable: weaners_room_temperature 
 Class: numeric
Min.  7.50
1st Qu. 16.80
Median 19.55
Mean 19.70
3rd Qu. 22.60
Max. 29.40
NA's 20
Variance: NA
Shapiro-Wilk p-value: 0.5194 (normal)
NA count: 20
```

## weaners\_airflow

**Summary statistics:**

```
 Variable: weaners_airflow 
 Class: numeric
Min. 0.0000
1st Qu. 0.1000
Median 0.1700
Mean 0.2074
3rd Qu. 0.2500
Max. 0.7000
NA's 23
Variance: NA
Shapiro-Wilk p-value: 0.0000003241 (not normal)
NA count: 23
```

## weaners\_air\_quality

**Summary statistics:**

```
 Variable: weaners_air_quality 
 Class: factor
1 6
2 79
3 9
NA's 6
Mode: 2
Proportion of levels: 1 0.06, 2 0.84, 3 0.10
Levels: 1, 2, 3
NA count: 6
```

## weaners\_airspace\_with\_other\_agegroup

**Summary statistics:**

```
 Variable: weaners_airspace_with_other_agegroup 
 Class: factor
2 23
3 52
4 19
NA's 6
Mode: 3
Proportion of levels: 2 0.24, 3 0.55, 4 0.20
Levels: 2, 3, 4
NA count: 6
```

## fattening\_pigs\_reduced\_general\_wellbeing

**Summary statistics:**

```
 Variable: fattening_pigs_reduced_general_wellbeing 
 Class: logical
FALSE 46
TRUE 1
Mode: FALSE
Proportion of levels: FALSE 0.98, TRUE 0.02
NA count: 53
```

## fattening\_pigs\_sneezing

**Summary statistics:**

```
 Variable: fattening_pigs_sneezing 
 Class: numeric
Min.  0.000
1st Qu.  0.000
Median  0.000
Mean  2.885
3rd Qu.  6.700
Max. 15.000
NA's 87
Variance: NA
Shapiro-Wilk p-value: 0.0002225 (not normal)
NA count: 87
```

## fattening\_pigs\_coughing

**Summary statistics:**

```
 Variable: fattening_pigs_coughing 
 Class: numeric
Min. 0.0000
1st Qu. 0.0000
Median 0.0000
Mean 0.5769
3rd Qu. 0.0000
Max. 4.8000
NA's 87
Variance: NA
Shapiro-Wilk p-value: 0.000005377 (not normal)
NA count: 87
```

## fattening\_pigs\_qm\_per\_animal

**Summary statistics:**

```
 Variable: fattening_pigs_qm_per_animal 
 Class: numeric
Min. 0.3000
1st Qu. 0.6725
Median 0.9450
Mean 1.0985
3rd Qu. 1.3150
Max. 4.7000
NA's 54
Variance: NA
Shapiro-Wilk p-value: 0.00000005605 (not normal)
NA count: 54
```

## fattening\_pigs\_feeding\_site\_per\_animal\_factor

**Summary statistics:**

```
 Variable: fattening_pigs_feeding_site_per_animal_factor 
 Class: factor
under 25 Animals/feeder 8
over 25 Animals/feeder 5
under 25cm trough/animal 10
over 25cm trough/animal 21
Other 3
NA 53
Mode: NA
Proportion of levels: under 25 Animals/feeder 0.08, over 25 Animals/feeder 0.05, under 25cm trough/animal 0.10, over 25cm trough/animal 0.21, Other 0.03, NA 0.53
Levels: under 25 Animals/feeder, over 25 Animals/feeder, under 25cm trough/animal, over 25cm trough/animal, Other, NA
NA count: 0
```

## fattening\_pigs\_animals\_per\_water\_source

**Summary statistics:**

```
 Variable: fattening_pigs_animals_per_water_source 
 Class: numeric
Min.  3.00
1st Qu.  8.65
Median 12.00
Mean 13.47
3rd Qu. 17.10
Max. 33.50
NA's 53
Variance: NA
Shapiro-Wilk p-value: 0.01312 (not normal)
NA count: 53
```

## fattening\_pigs\_room\_temperature

**Summary statistics:**

```
 Variable: fattening_pigs_room_temperature 
 Class: numeric
Min.  2.90
1st Qu. 16.57
Median 19.85
Mean 19.48
3rd Qu. 22.23
Max. 31.50
NA's 60
Variance: NA
Shapiro-Wilk p-value: 0.1944 (normal)
NA count: 60
```

## fattening\_pigs\_airflow

**Summary statistics:**

```
 Variable: fattening_pigs_airflow 
 Class: numeric
Min. 0.0400
1st Qu. 0.1500
Median 0.2000
Mean 0.2372
3rd Qu. 0.3000
Max. 0.6000
NA's 61
Variance: NA
Shapiro-Wilk p-value: 0.001534 (not normal)
NA count: 61
```

## fattening\_pigs\_air\_quality

**Summary statistics:**

```
 Variable: fattening_pigs_air_quality 
 Class: factor
1 4
2 32
3 11
NA's 53
Mode: 2
Proportion of levels: 1 0.09, 2 0.68, 3 0.23
Levels: 1, 2, 3
NA count: 53
```

## fattening\_pigs\_airspace\_with\_other\_agegroup

**Summary statistics:**

```
 Variable: fattening_pigs_airspace_with_other_agegroup 
 Class: factor
1 9
2 16
3 13
4 9
NA's 53
Mode: 2
Proportion of levels: 1 0.19, 2 0.34, 3 0.28, 4 0.19
Levels: 1, 2, 3, 4
NA count: 53
```

## report\_killed\_weaners

**Summary statistics:**

```
 Variable: report_killed_weaners 
 Class: logical
FALSE 97
TRUE 3
Mode: FALSE
Proportion of levels: FALSE 0.97, TRUE 0.03
NA count: 0
```

## report\_killed\_fattening\_pigs

**Summary statistics:**

```
 Variable: report_killed_fattening_pigs 
 Class: logical
FALSE 98
TRUE 2
Mode: FALSE
Proportion of levels: FALSE 0.98, TRUE 0.02
NA count: 0
```

## report\_killed\_young\_sows

**Summary statistics:**

```
 Variable: report_killed_young_sows 
 Class: logical
FALSE 99
TRUE 1
Mode: FALSE
Proportion of levels: FALSE 0.99, TRUE 0.01
NA count: 0
```

## report\_killed\_old\_sows

**Summary statistics:**

```
 Variable: report_killed_old_sows 
 Class: logical
FALSE 99
TRUE 1
Mode: FALSE
Proportion of levels: FALSE 0.99, TRUE 0.01
NA count: 0
```

---

Exposures

## IAV\_positive

**Summary statistics:**

```
 Variable: IAV_positive 
 Class: logical
FALSE 65
TRUE 35
Mode: FALSE
Proportion of levels: FALSE 0.65, TRUE 0.35
NA count: 0
```

## total\_samples

**Summary statistics:**

```
 Variable: total_samples 
 Class: integer
Min.  9.00
1st Qu. 19.00
Median 19.00
Mean 18.49
3rd Qu. 19.00
Max. 19.00
Variance: 2.475
Shapiro-Wilk p-value: 0.000000000000000001131 (not normal)
NA count: 0
```

## positive\_pigs

**Summary statistics:**

```
 Variable: positive_pigs 
 Class: integer
Min.  0.00
1st Qu.  0.00
Median  0.00
Mean  1.59
3rd Qu.  1.00
Max. 19.00
Variance: 14.06
Shapiro-Wilk p-value: 0.00000000000000004751 (not normal)
NA count: 0
```

## percent\_positive\_pigs

**Summary statistics:**

```
 Variable: percent_positive_pigs 
 Class: numeric
Min.   0.000
1st Qu.   0.000
Median   0.000
Mean   8.485
3rd Qu.   5.263
Max. 100.000
Variance: 406.6
Shapiro-Wilk p-value: 0.00000000000000003892 (not normal)
NA count: 0
```

## min\_ct

**Summary statistics:**

```
 Variable: min_ct 
 Class: numeric
Min. 20.61
1st Qu. 31.24
Median 34.69
Mean 33.11
3rd Qu. 36.96
Max. 37.73
NA's 65
Variance: NA
Shapiro-Wilk p-value: 0.00006768 (not normal)
NA count: 65
```

## max\_ct

**Summary statistics:**

```
 Variable: max_ct 
 Class: numeric
Min. 29.21
1st Qu. 34.68
Median 35.14
Mean 35.64
3rd Qu. 37.34
Max. 37.73
NA's 65
Variance: NA
Shapiro-Wilk p-value: 0.0003468 (not normal)
NA count: 65
```

## mean\_ct

**Summary statistics:**

```
 Variable: mean_ct 
 Class: numeric
Min. 28.18
1st Qu. 33.68
Median 34.75
Mean 34.68
3rd Qu. 36.96
Max. 37.73
NA's 65
Variance: NA
Shapiro-Wilk p-value: 0.003306 (not normal)
NA count: 65
```

## sd\_ct

**Summary statistics:**

```
 Variable: sd_ct 
 Class: numeric
Min. 0.3458
1st Qu. 0.4751
Median 1.1683
Mean 1.8308
3rd Qu. 2.8151
Max. 5.1829
NA's 82
Variance: NA
Shapiro-Wilk p-value: 0.002632 (not normal)
NA count: 82
```

## season\_sampling

**Summary statistics:**

```
 Variable: season_sampling 
 Class: factor
winter 23
spring 32
summer 25
autumn 20
Mode: spring
Proportion of levels: winter 0.23, spring 0.32, summer 0.25, autumn 0.20
Levels: winter, spring, summer, autumn
NA count: 0
```

## canton\_factor

**Summary statistics:**

```
 Variable: canton_factor 
 Class: factor
North-West 6
Berne + Solothurn 42
East & Zurich 19
Romandy 14
Lucerne 19
Mode: Berne + Solothurn
Proportion of levels: North-West 0.06, Berne + Solothurn 0.42, East & Zurich 0.19, Romandy 0.14, Lucerne 0.19
Levels: North-West, Berne + Solothurn, East & Zurich, Romandy, Lucerne
NA count: 0
```

## herdsize

**Summary statistics:**

```
 Variable: herdsize 
 Class: integer
Min.   25
1st Qu.  462
Median  720
Mean  843
3rd Qu. 1114
Max. 4183
Variance: 344813
Shapiro-Wilk p-value: 0.000000007035 (not normal)
NA count: 0
```

## production\_type\_factor

**Summary statistics:**

```
 Variable: production_type_factor 
 Class: factor
1 11
2 25
3 41
4 14
5 9
Mode: 3
Proportion of levels: 1 0.11, 2 0.25, 3 0.41, 4 0.14, 5 0.09
Levels: 1, 2, 3, 4, 5
NA count: 0
```

## Farrowing\_on\_farm

**Summary statistics:**

```
 Variable: Farrowing_on_farm 
 Class: logical
FALSE 12
TRUE 88
Mode: TRUE
Proportion of levels: FALSE 0.12, TRUE 0.88
NA count: 0
```

## Isemination\_on\_farm

**Summary statistics:**

```
 Variable: Isemination_on_farm 
 Class: logical
FALSE 30
TRUE 70
Mode: TRUE
Proportion of levels: FALSE 0.30, TRUE 0.70
NA count: 0
```

## Gestation\_on\_farm

**Summary statistics:**

```
 Variable: Gestation_on_farm 
 Class: logical
FALSE 30
TRUE 70
Mode: TRUE
Proportion of levels: FALSE 0.30, TRUE 0.70
NA count: 0
```

## Weaners\_on\_farm

**Summary statistics:**

```
 Variable: Weaners_on_farm 
 Class: logical
FALSE 11
TRUE 89
Mode: TRUE
Proportion of levels: FALSE 0.11, TRUE 0.89
NA count: 0
```

## Fattening\_on\_farm

**Summary statistics:**

```
 Variable: Fattening_on_farm 
 Class: logical
FALSE 37
TRUE 63
Mode: TRUE
Proportion of levels: FALSE 0.37, TRUE 0.63
NA count: 0
```

## respiratory\_signs

**Summary statistics:**

```
 Variable: respiratory_signs 
 Class: logical
FALSE 85
TRUE 15
Mode: FALSE
Proportion of levels: FALSE 0.85, TRUE 0.15
NA count: 0
```

## rectal\_temperature\_max

**Summary statistics:**

```
 Variable: rectal_temperature_max 
 Class: numeric
Min. 39.20
1st Qu. 40.10
Median 40.30
Mean 40.40
3rd Qu. 40.65
Max. 41.80
NA's 1
Variance: NA
Shapiro-Wilk p-value: 0.004161 (not normal)
NA count: 1
```

## rectal\_temperature\_avg

**Summary statistics:**

```
 Variable: rectal_temperature_avg 
 Class: numeric
Min. 38.80
1st Qu. 39.50
Median 39.70
Mean 39.67
3rd Qu. 39.90
Max. 40.40
NA's 1
Variance: NA
Shapiro-Wilk p-value: 0.02114 (not normal)
NA count: 1
```

## horses\_closeby

**Summary statistics:**

```
 Variable: horses_closeby 
 Class: logical
FALSE 92
TRUE 8
Mode: FALSE
Proportion of levels: FALSE 0.92, TRUE 0.08
NA count: 0
```

## dogs\_closeby

**Summary statistics:**

```
 Variable: dogs_closeby 
 Class: logical
FALSE 55
TRUE 45
Mode: FALSE
Proportion of levels: FALSE 0.55, TRUE 0.45
NA count: 0
```

## chicken\_closeby

**Summary statistics:**

```
 Variable: chicken_closeby 
 Class: logical
FALSE 67
TRUE 33
Mode: FALSE
Proportion of levels: FALSE 0.67, TRUE 0.33
NA count: 0
```

## turkey\_closeby

**Summary statistics:**

```
 Variable: turkey_closeby 
 Class: logical
FALSE 98
TRUE 2
Mode: FALSE
Proportion of levels: FALSE 0.98, TRUE 0.02
NA count: 0
```

## cattle\_closeby

**Summary statistics:**

```
 Variable: cattle_closeby 
 Class: logical
FALSE 49
TRUE 51
Mode: TRUE
Proportion of levels: FALSE 0.49, TRUE 0.51
NA count: 0
```

## cats\_closeby

**Summary statistics:**

```
 Variable: cats_closeby 
 Class: logical
FALSE 35
TRUE 65
Mode: TRUE
Proportion of levels: FALSE 0.35, TRUE 0.65
NA count: 0
```

## proximity\_to\_other\_pig\_herd

**Summary statistics:**

```
 Variable: proximity_to_other_pig_herd 
 Class: logical
FALSE 33
TRUE 67
Mode: TRUE
Proportion of levels: FALSE 0.33, TRUE 0.67
NA count: 0
```

## proximity\_to\_other\_poultry\_herd

**Summary statistics:**

```
 Variable: proximity_to_other_poultry_herd 
 Class: logical
FALSE 23
TRUE 77
Mode: TRUE
Proportion of levels: FALSE 0.23, TRUE 0.77
NA count: 0
```

## number\_suckling\_piglets

**Summary statistics:**

```
 Variable: number_suckling_piglets 
 Class: integer
Min.   20.0
1st Qu.  150.0
Median  242.5
Mean  275.6
3rd Qu.  350.0
Max. 1400.0
NA's 12
Variance: NA
Shapiro-Wilk p-value: 0.000000003806 (not normal)
NA count: 12
```

## number\_weaners

**Summary statistics:**

```
 Variable: number_weaners 
 Class: integer
Min.   16.0
1st Qu.  200.0
Median  300.0
Mean  368.5
3rd Qu.  500.0
Max. 2200.0
NA's 11
Variance: NA
Shapiro-Wilk p-value: 0.0000000001275 (not normal)
NA count: 11
```

## number\_fattening\_pigs

**Summary statistics:**

```
 Variable: number_fattening_pigs 
 Class: integer
Min.    1
1st Qu.   20
Median  184
Mean  283
3rd Qu.  470
Max. 1400
NA's 37
Variance: NA
Shapiro-Wilk p-value: 0.000001303 (not normal)
NA count: 37
```

## number\_young\_sows

**Summary statistics:**

```
 Variable: number_young_sows 
 Class: integer
Min.   2.0
1st Qu.   7.0
Median  10.0
Mean  22.4
3rd Qu.  20.0
Max. 350.0
NA's 28
Variance: NA
Shapiro-Wilk p-value: 0.0000000000000004535 (not normal)
NA count: 28
```

## number\_old\_sows

**Summary statistics:**

```
 Variable: number_old_sows 
 Class: integer
Min.   5.0
1st Qu.  40.0
Median  75.0
Mean  87.4
3rd Qu. 116.2
Max. 530.0
NA's 12
Variance: NA
Shapiro-Wilk p-value: 0.0000000003521 (not normal)
NA count: 12
```

## number\_boars

**Summary statistics:**

```
 Variable: number_boars 
 Class: integer
Min. 1.0
1st Qu. 1.0
Median 2.0
Mean 1.8
3rd Qu. 2.0
Max. 4.0
NA's 30
Variance: NA
Shapiro-Wilk p-value: 0.00000002447 (not normal)
NA count: 30
```

## number\_of\_origins

**Summary statistics:**

```
 Variable: number_of_origins 
 Class: integer
Min. 1.000
1st Qu. 1.000
Median 2.000
Mean 1.667
3rd Qu. 2.000
Max. 4.000
NA's 13
Variance: NA
Shapiro-Wilk p-value: 0.00000000009154 (not normal)
NA count: 13
```

## quarantine\_concept

**Summary statistics:**

```
 Variable: quarantine_concept 
 Class: logical
FALSE 34
TRUE 66
Mode: TRUE
Proportion of levels: FALSE 0.34, TRUE 0.66
NA count: 0
```

## quarantine\_time

**Summary statistics:**

```
 Variable: quarantine_time 
 Class: factor
16 12
4 10
3 10
6 6
5 4
20 3
2 1
other 12
NA's 42
Mode: 16
Proportion of levels: 16 0.21, 4 0.17, 3 0.17, 6 0.10, 5 0.07, 20 0.05, 2 0.02, other 0.21
Levels: 16, 4, 3, 6, 5, 20, 2, other
NA count: 42
```

## quarantine\_in\_herd\_contact

**Summary statistics:**

```
 Variable: quarantine_in_herd_contact 
 Class: logical
FALSE 62
TRUE 38
Mode: FALSE
Proportion of levels: FALSE 0.62, TRUE 0.38
NA count: 0
```

## herds\_of\_origin\_respiratory\_symptoms

**Summary statistics:**

```
 Variable: herds_of_origin_respiratory_symptoms 
 Class: logical
FALSE 96
TRUE 4
Mode: FALSE
Proportion of levels: FALSE 0.96, TRUE 0.04
NA count: 0
```

## herds\_of\_origin\_influenza\_diagnosis

**Summary statistics:**

```
 Variable: herds_of_origin_influenza_diagnosis 
 Class: logical
FALSE 99
TRUE 1
Mode: FALSE
Proportion of levels: FALSE 0.99, TRUE 0.01
NA count: 0
```

## production\_cycle

**Summary statistics:**

```
 Variable: production_cycle 
 Class: factor
1 15
2 16
3 47
other 22
Mode: 3
Proportion of levels: 1 0.15, 2 0.16, 3 0.47, other 0.22
Levels: 1, 2, 3, other
NA count: 0
```

## mode\_stable\_occupation\_ai\_centre

**Summary statistics:**

```
 Variable: mode_stable_occupation_ai_centre 
 Class: logical
FALSE 13
TRUE 55
Mode: TRUE
Proportion of levels: FALSE 0.19, TRUE 0.81
NA count: 32
```

## mode\_stable\_occupation\_gestation\_stable

**Summary statistics:**

```
 Variable: mode_stable_occupation_gestation_stable 
 Class: logical
FALSE 10
TRUE 64
Mode: TRUE
Proportion of levels: FALSE 0.14, TRUE 0.86
NA count: 26
```

## mode\_stable\_occupation\_farrowing\_stable

**Summary statistics:**

```
 Variable: mode_stable_occupation_farrowing_stable 
 Class: logical
FALSE 24
TRUE 65
Mode: TRUE
Proportion of levels: FALSE 0.27, TRUE 0.73
NA count: 11
```

## cross\_fostering\_farrowing\_stable

**Summary statistics:**

```
 Variable: cross_fostering_farrowing_stable 
 Class: factor
1 19
2 48
3 22
NA's 11
Mode: 2
Proportion of levels: 1 0.21, 2 0.54, 3 0.25
Levels: 1, 2, 3
NA count: 11
```

## mode\_stable\_occupation\_weaner\_stable

**Summary statistics:**

```
 Variable: mode_stable_occupation_weaner_stable 
 Class: logical
FALSE 43
TRUE 48
Mode: TRUE
Proportion of levels: FALSE 0.47, TRUE 0.53
NA count: 9
```

## mode\_stable\_occupation\_fattening\_stable

**Summary statistics:**

```
 Variable: mode_stable_occupation_fattening_stable 
 Class: logical
FALSE 54
TRUE 9
Mode: FALSE
Proportion of levels: FALSE 0.86, TRUE 0.14
NA count: 37
```

## passing\_through\_other\_age\_group

**Summary statistics:**

```
 Variable: passing_through_other_age_group 
 Class: logical
FALSE 60
TRUE 40
Mode: FALSE
Proportion of levels: FALSE 0.60, TRUE 0.40
NA count: 0
```

## outside\_area

**Summary statistics:**

```
 Variable: outside_area 
 Class: logical
FALSE 25
TRUE 75
Mode: TRUE
Proportion of levels: FALSE 0.25, TRUE 0.75
NA count: 0
```

## outside\_area\_ai\_centre

**Summary statistics:**

```
 Variable: outside_area_ai_centre 
 Class: logical
FALSE 71
TRUE 29
Mode: FALSE
Proportion of levels: FALSE 0.71, TRUE 0.29
NA count: 0
```

## outside\_area\_gestation\_stable

**Summary statistics:**

```
 Variable: outside_area_gestation_stable 
 Class: logical
FALSE 43
TRUE 57
Mode: TRUE
Proportion of levels: FALSE 0.43, TRUE 0.57
NA count: 0
```

## outside\_area\_farrowing\_stable

**Summary statistics:**

```
 Variable: outside_area_farrowing_stable 
 Class: logical
FALSE 94
TRUE 6
Mode: FALSE
Proportion of levels: FALSE 0.94, TRUE 0.06
NA count: 0
```

## outside\_area\_weaner\_stable

**Summary statistics:**

```
 Variable: outside_area_weaner_stable 
 Class: logical
FALSE 90
TRUE 10
Mode: FALSE
Proportion of levels: FALSE 0.90, TRUE 0.10
NA count: 0
```

## outside\_area\_fattening\_stable

**Summary statistics:**

```
 Variable: outside_area_fattening_stable 
 Class: logical
FALSE 63
TRUE 37
Mode: FALSE
Proportion of levels: FALSE 0.63, TRUE 0.37
NA count: 0
```

## outside\_area\_contact\_poultry

**Summary statistics:**

```
 Variable: outside_area_contact_poultry 
 Class: logical
FALSE 93
TRUE 7
Mode: FALSE
Proportion of levels: FALSE 0.93, TRUE 0.07
NA count: 0
```

## outside\_area\_contact\_wild\_birds

**Summary statistics:**

```
 Variable: outside_area_contact_wild_birds 
 Class: logical
FALSE 31
TRUE 69
Mode: TRUE
Proportion of levels: FALSE 0.31, TRUE 0.69
NA count: 0
```

## outside\_area\_contact\_wild\_boars

**Summary statistics:**

```
 Variable: outside_area_contact_wild_boars 
 Class: logical
FALSE 45
TRUE 55
Mode: TRUE
Proportion of levels: FALSE 0.45, TRUE 0.55
NA count: 0
```

## contact\_bird\_in\_stable

**Summary statistics:**

```
 Variable: contact_bird_in_stable 
 Class: logical
FALSE 61
TRUE 39
Mode: FALSE
Proportion of levels: FALSE 0.61, TRUE 0.39
NA count: 0
```

## cleaning\_ai\_centre

**Summary statistics:**

```
 Variable: cleaning_ai_centre 
 Class: factor
1 18
2 40
3 11
NA's 31
Mode: 2
Proportion of levels: 1 0.26, 2 0.58, 3 0.16
Levels: 1, 2, 3
NA count: 31
```

## cleaning\_gestation\_stable

**Summary statistics:**

```
 Variable: cleaning_gestation_stable 
 Class: logical
FALSE 26
TRUE 49
Mode: TRUE
Proportion of levels: FALSE 0.35, TRUE 0.65
NA count: 25
```

## cleaning\_farrowing\_stable

**Summary statistics:**

```
 Variable: cleaning_farrowing_stable 
 Class: logical
FALSE 4
TRUE 85
Mode: TRUE
Proportion of levels: FALSE 0.04, TRUE 0.96
NA count: 11
```

## cleaning\_weaner\_stable

**Summary statistics:**

```
 Variable: cleaning_weaner_stable 
 Class: logical
FALSE 11
TRUE 79
Mode: TRUE
Proportion of levels: FALSE 0.12, TRUE 0.88
NA count: 10
```

## cleaning\_fattening\_stable

**Summary statistics:**

```
 Variable: cleaning_fattening_stable 
 Class: factor
1 13
2 36
3 14
NA's 37
Mode: 2
Proportion of levels: 1 0.21, 2 0.57, 3 0.22
Levels: 1, 2, 3
NA count: 37
```

## cleaning\_quarantine

**Summary statistics:**

```
 Variable: cleaning_quarantine 
 Class: factor
1 9
2 7
3 8
NA's 76
Mode: 1
Proportion of levels: 1 0.38, 2 0.29, 3 0.33
Levels: 1, 2, 3
NA count: 76
```

## disinfection\_ai\_centre

**Summary statistics:**

```
 Variable: disinfection_ai_centre 
 Class: logical
FALSE 54
TRUE 15
Mode: FALSE
Proportion of levels: FALSE 0.78, TRUE 0.22
NA count: 31
```

## disinfection\_gestation\_stable

**Summary statistics:**

```
 Variable: disinfection_gestation_stable 
 Class: logical
FALSE 62
TRUE 13
Mode: FALSE
Proportion of levels: FALSE 0.83, TRUE 0.17
NA count: 25
```

## disinfection\_farrowing\_stable

**Summary statistics:**

```
 Variable: disinfection_farrowing_stable 
 Class: factor
1 47
2 9
3 34
NA's 10
Mode: 1
Proportion of levels: 1 0.52, 2 0.10, 3 0.38
Levels: 1, 2, 3
NA count: 10
```

## disinfection\_weaner\_stable

**Summary statistics:**

```
 Variable: disinfection_weaner_stable 
 Class: factor
1 52
2 10
3 28
NA's 10
Mode: 1
Proportion of levels: 1 0.58, 2 0.11, 3 0.31
Levels: 1, 2, 3
NA count: 10
```

## disinfection\_fattening\_stable

**Summary statistics:**

```
 Variable: disinfection_fattening_stable 
 Class: logical
FALSE 48
TRUE 15
Mode: FALSE
Proportion of levels: FALSE 0.76, TRUE 0.24
NA count: 37
```

## disinfection\_quarantine

**Summary statistics:**

```
 Variable: disinfection_quarantine 
 Class: logical
FALSE 18
TRUE 6
Mode: FALSE
Proportion of levels: FALSE 0.75, TRUE 0.25
NA count: 76
```

## drying\_ai\_centre

**Summary statistics:**

```
 Variable: drying_ai_centre 
 Class: factor
1 28
2 29
3 12
NA's 31
Mode: 2
Proportion of levels: 1 0.41, 2 0.42, 3 0.17
Levels: 1, 2, 3
NA count: 31
```

## drying\_gestation\_stable

**Summary statistics:**

```
 Variable: drying_gestation_stable 
 Class: logical
FALSE 46
TRUE 29
Mode: FALSE
Proportion of levels: FALSE 0.61, TRUE 0.39
NA count: 25
```

## drying\_farrowing\_stable

**Summary statistics:**

```
 Variable: drying_farrowing_stable 
 Class: logical
FALSE 12
TRUE 77
Mode: TRUE
Proportion of levels: FALSE 0.13, TRUE 0.87
NA count: 11
```

## drying\_weaner\_stable

**Summary statistics:**

```
 Variable: drying_weaner_stable 
 Class: logical
FALSE 9
TRUE 81
Mode: TRUE
Proportion of levels: FALSE 0.10, TRUE 0.90
NA count: 10
```

## drying\_fattening\_stable

**Summary statistics:**

```
 Variable: drying_fattening_stable 
 Class: factor
1 22
2 27
3 13
NA's 38
Mode: 2
Proportion of levels: 1 0.35, 2 0.44, 3 0.21
Levels: 1, 2, 3
NA count: 38
```

## drying\_quarantine

**Summary statistics:**

```
 Variable: drying_quarantine 
 Class: logical
FALSE 13
TRUE 11
Mode: FALSE
Proportion of levels: FALSE 0.54, TRUE 0.46
NA count: 76
```

## cleaning\_disinfection\_transport\_vehicle

**Summary statistics:**

```
 Variable: cleaning_disinfection_transport_vehicle 
 Class: logical
FALSE 4
TRUE 77
Mode: TRUE
Proportion of levels: FALSE 0.05, TRUE 0.95
NA count: 19
```

## cleaning\_shipment\_area

**Summary statistics:**

```
 Variable: cleaning_shipment_area 
 Class: logical
FALSE 61
TRUE 35
Mode: FALSE
Proportion of levels: FALSE 0.64, TRUE 0.36
NA count: 4
```

## caretaker\_number

**Summary statistics:**

```
 Variable: caretaker_number 
 Class: integer
Min.  1.00
1st Qu.  2.00
Median  3.00
Mean  2.85
3rd Qu.  3.00
Max. 16.00
Variance: 2.876
Shapiro-Wilk p-value: 0.000000000000004927 (not normal)
NA count: 0
```

## caretaker\_ppe\_stable

**Summary statistics:**

```
 Variable: caretaker_ppe_stable 
 Class: logical
FALSE 35
TRUE 65
Mode: TRUE
Proportion of levels: FALSE 0.35, TRUE 0.65
NA count: 0
```

## caretaker\_ppe\_washing\_interval

**Summary statistics:**

```
 Variable: caretaker_ppe_washing_interval 
 Class: logical
FALSE 12
TRUE 88
Mode: TRUE
Proportion of levels: FALSE 0.12, TRUE 0.88
NA count: 0
```

## caretaker\_ppe\_per\_unit

**Summary statistics:**

```
 Variable: caretaker_ppe_per_unit 
 Class: logical
FALSE 94
TRUE 6
Mode: FALSE
Proportion of levels: FALSE 0.94, TRUE 0.06
NA count: 0
```

## caretaker\_per\_unit

**Summary statistics:**

```
 Variable: caretaker_per_unit 
 Class: logical
FALSE 79
TRUE 21
Mode: FALSE
Proportion of levels: FALSE 0.79, TRUE 0.21
NA count: 0
```

## caretaker\_work\_flow\_hygiene\_between\_units

**Summary statistics:**

```
 Variable: caretaker_work_flow_hygiene_between_units 
 Class: logical
FALSE 31
TRUE 69
Mode: TRUE
Proportion of levels: FALSE 0.31, TRUE 0.69
NA count: 0
```

## caretaker\_entry\_ppe\_only

**Summary statistics:**

```
 Variable: caretaker_entry_ppe_only 
 Class: logical
FALSE 49
TRUE 51
Mode: TRUE
Proportion of levels: FALSE 0.49, TRUE 0.51
NA count: 0
```

## caretaker\_disease\_management

**Summary statistics:**

```
 Variable: caretaker_disease_management 
 Class: logical
FALSE 87
TRUE 12
Mode: FALSE
Proportion of levels: FALSE 0.88, TRUE 0.12
NA count: 1
```

## caretaker\_hands\_washed\_before\_entry

**Summary statistics:**

```
 Variable: caretaker_hands_washed_before_entry 
 Class: logical
FALSE 55
TRUE 45
Mode: FALSE
Proportion of levels: FALSE 0.55, TRUE 0.45
NA count: 0
```

## caretaker\_boot\_disinfection

**Summary statistics:**

```
 Variable: caretaker_boot_disinfection 
 Class: logical
FALSE 78
TRUE 22
Mode: FALSE
Proportion of levels: FALSE 0.78, TRUE 0.22
NA count: 0
```

## caretaker\_contact\_other\_pigs

**Summary statistics:**

```
 Variable: caretaker_contact_other_pigs 
 Class: logical
FALSE 78
TRUE 7
Mode: FALSE
Proportion of levels: FALSE 0.92, TRUE 0.08
NA count: 15
```

## caretaker\_contact\_poultry

**Summary statistics:**

```
 Variable: caretaker_contact_poultry 
 Class: logical
FALSE 71
TRUE 8
Mode: FALSE
Proportion of levels: FALSE 0.90, TRUE 0.10
NA count: 21
```

## visitors\_cumulative\_contact\_hours

**Summary statistics:**

```
 Variable: visitors_cumulative_contact_hours 
 Class: numeric
Min.  1.00
1st Qu.  3.00
Median  6.00
Mean  7.03
3rd Qu.  9.00
Max. 22.00
Variance: 24.09
Shapiro-Wilk p-value: 0.000001059 (not normal)
NA count: 0
```

## visitors\_list

**Summary statistics:**

```
 Variable: visitors_list 
 Class: logical
FALSE 3
TRUE 97
Mode: TRUE
Proportion of levels: FALSE 0.03, TRUE 0.97
NA count: 0
```

## ppe\_visitors

**Summary statistics:**

```
 Variable: ppe_visitors 
 Class: logical
FALSE 5
TRUE 95
Mode: TRUE
Proportion of levels: FALSE 0.05, TRUE 0.95
NA count: 0
```

## visitors\_hands\_washed\_before\_entry

**Summary statistics:**

```
 Variable: visitors_hands_washed_before_entry 
 Class: logical
FALSE 50
TRUE 50
Mode: FALSE
Proportion of levels: FALSE 0.50, TRUE 0.50
NA count: 0
```

## visitors\_disease\_management

**Summary statistics:**

```
 Variable: visitors_disease_management 
 Class: logical
FALSE 20
TRUE 5
Mode: FALSE
Proportion of levels: FALSE 0.80, TRUE 0.20
NA count: 75
```

## visitors\_contact\_other\_pigs

**Summary statistics:**

```
 Variable: visitors_contact_other_pigs 
 Class: logical
FALSE 9
TRUE 67
Mode: TRUE
Proportion of levels: FALSE 0.12, TRUE 0.88
NA count: 24
```

## visitors\_respiratory\_symptoms

**Summary statistics:**

```
 Variable: visitors_respiratory_symptoms 
 Class: logical
FALSE 98
TRUE 2
Mode: FALSE
Proportion of levels: FALSE 0.98, TRUE 0.02
NA count: 0
```

## return\_to\_service\_rate

**Summary statistics:**

```
 Variable: return_to_service_rate 
 Class: logical
FALSE 14
TRUE 54
Mode: TRUE
Proportion of levels: FALSE 0.21, TRUE 0.79
NA count: 32
```

## farrowing\_rate

**Summary statistics:**

```
 Variable: farrowing_rate 
 Class: logical
FALSE 19
TRUE 62
Mode: TRUE
Proportion of levels: FALSE 0.23, TRUE 0.77
NA count: 19
```

## piglets\_per\_sow\_year

**Summary statistics:**

```
 Variable: piglets_per_sow_year 
 Class: logical
FALSE 8
TRUE 74
Mode: TRUE
Proportion of levels: FALSE 0.10, TRUE 0.90
NA count: 18
```

## abortions\_per\_sow\_year

**Summary statistics:**

```
 Variable: abortions_per_sow_year 
 Class: logical
FALSE 1
TRUE 81
Mode: TRUE
Proportion of levels: FALSE 0.01, TRUE 0.99
NA count: 18
```

## piglet\_mortality

**Summary statistics:**

```
 Variable: piglet_mortality 
 Class: logical
FALSE 15
TRUE 66
Mode: TRUE
Proportion of levels: FALSE 0.19, TRUE 0.81
NA count: 19
```

## feed\_conversion\_rate\_fattening\_pigs

**Summary statistics:**

```
 Variable: feed_conversion_rate_fattening_pigs 
 Class: logical
FALSE 3
TRUE 11
Mode: TRUE
Proportion of levels: FALSE 0.21, TRUE 0.79
NA count: 86
```

## respiratory\_history\_swine

**Summary statistics:**

```
 Variable: respiratory_history_swine 
 Class: factor
1 54
2 20
3 5
NA's 21
Mode: 1
Proportion of levels: 1 0.68, 2 0.25, 3 0.06
Levels: 1, 2, 3
NA count: 21
```

## time\_respiratory\_disease

**Summary statistics:**

```
 Variable: time_respiratory_disease 
 Class: logical
FALSE 6
TRUE 52
Mode: TRUE
Proportion of levels: FALSE 0.10, TRUE 0.90
NA count: 42
```

## frequency\_respi\_outbreak

**Summary statistics:**

```
 Variable: frequency_respi_outbreak 
 Class: factor
never 36
few pigs affected no outbreak 19
once 23
1x/year 11
2x/year 7
3x or more per year 4
Mode: never
Proportion of levels: never 0.36, few pigs affected no outbreak 0.19, once 0.23, 1x/year 0.11, 2x/year 0.07, 3x or more per year 0.04
Levels: never, few pigs affected no outbreak, once, 1x/year, 2x/year, 3x or more per year
NA count: 0
```

## outbreak\_since\_examination

**Summary statistics:**

```
 Variable: outbreak_since_examination 
 Class: logical
FALSE 95
TRUE 5
Mode: FALSE
Proportion of levels: FALSE 0.95, TRUE 0.05
NA count: 0
```

## suckling\_piglets\_diseased

**Summary statistics:**

```
 Variable: suckling_piglets_diseased 
 Class: logical
FALSE 94
TRUE 6
Mode: FALSE
Proportion of levels: FALSE 0.94, TRUE 0.06
NA count: 0
```

## weaners\_diseased

**Summary statistics:**

```
 Variable: weaners_diseased 
 Class: logical
FALSE 55
TRUE 45
Mode: FALSE
Proportion of levels: FALSE 0.55, TRUE 0.45
NA count: 0
```

## fattening\_pigs\_diseased

**Summary statistics:**

```
 Variable: fattening_pigs_diseased 
 Class: logical
FALSE 73
TRUE 27
Mode: FALSE
Proportion of levels: FALSE 0.73, TRUE 0.27
NA count: 0
```

## young\_sows\_diseased

**Summary statistics:**

```
 Variable: young_sows_diseased 
 Class: logical
FALSE 97
TRUE 3
Mode: FALSE
Proportion of levels: FALSE 0.97, TRUE 0.03
NA count: 0
```

## old\_sows\_diseased

**Summary statistics:**

```
 Variable: old_sows_diseased 
 Class: logical
FALSE 97
TRUE 3
Mode: FALSE
Proportion of levels: FALSE 0.97, TRUE 0.03
NA count: 0
```

## boars\_diseased

**Summary statistics:**

```
 Variable: boars_diseased 
 Class: logical
FALSE 99
TRUE 1
Mode: FALSE
Proportion of levels: FALSE 0.99, TRUE 0.01
NA count: 0
```

## symptom\_swine\_sneezing

**Summary statistics:**

```
 Variable: symptom_swine_sneezing 
 Class: logical
FALSE 80
TRUE 20
Mode: FALSE
Proportion of levels: FALSE 0.80, TRUE 0.20
NA count: 0
```

## symptom\_swine\_coughing

**Summary statistics:**

```
 Variable: symptom_swine_coughing 
 Class: logical
FALSE 37
TRUE 63
Mode: TRUE
Proportion of levels: FALSE 0.37, TRUE 0.63
NA count: 0
```

## symptom\_swine\_nasal\_discharge

**Summary statistics:**

```
 Variable: symptom_swine_nasal_discharge 
 Class: logical
FALSE 94
TRUE 6
Mode: FALSE
Proportion of levels: FALSE 0.94, TRUE 0.06
NA count: 0
```

## symptom\_swine\_fever

**Summary statistics:**

```
 Variable: symptom_swine_fever 
 Class: logical
FALSE 85
TRUE 15
Mode: FALSE
Proportion of levels: FALSE 0.85, TRUE 0.15
NA count: 0
```

## symptom\_swine\_feed\_intake\_red

**Summary statistics:**

```
 Variable: symptom_swine_feed_intake_red 
 Class: logical
FALSE 66
TRUE 34
Mode: FALSE
Proportion of levels: FALSE 0.66, TRUE 0.34
NA count: 0
```

## symptom\_swine\_apathy

**Summary statistics:**

```
 Variable: symptom_swine_apathy 
 Class: logical
FALSE 74
TRUE 26
Mode: FALSE
Proportion of levels: FALSE 0.74, TRUE 0.26
NA count: 0
```

## symptom\_swine\_dyspnoea

**Summary statistics:**

```
 Variable: symptom_swine_dyspnoea 
 Class: logical
FALSE 87
TRUE 13
Mode: FALSE
Proportion of levels: FALSE 0.87, TRUE 0.13
NA count: 0
```

## vet\_consultation

**Summary statistics:**

```
 Variable: vet_consultation 
 Class: logical
FALSE 79
TRUE 21
Mode: FALSE
Proportion of levels: FALSE 0.79, TRUE 0.21
NA count: 0
```

## influenza\_diagnosis

**Summary statistics:**

```
 Variable: influenza_diagnosis 
 Class: logical
FALSE 96
TRUE 4
Mode: FALSE
Proportion of levels: FALSE 0.96, TRUE 0.04
NA count: 0
```

## influenza\_vaccination

**Summary statistics:**

```
 Variable: influenza_vaccination 
 Class: logical
FALSE 98
TRUE 2
Mode: FALSE
Proportion of levels: FALSE 0.98, TRUE 0.02
NA count: 0
```

## respiratory\_history\_human

**Summary statistics:**

```
 Variable: respiratory_history_human 
 Class: logical
FALSE 88
TRUE 12
Mode: FALSE
Proportion of levels: FALSE 0.88, TRUE 0.12
NA count: 0
```

## respiratory\_history\_contact\_person

**Summary statistics:**

```
 Variable: respiratory_history_contact_person 
 Class: logical
FALSE 94
TRUE 6
Mode: FALSE
Proportion of levels: FALSE 0.94, TRUE 0.06
NA count: 0
```

## symptom\_human\_sneezing

**Summary statistics:**

```
 Variable: symptom_human_sneezing 
 Class: logical
FALSE 88
TRUE 12
Mode: FALSE
Proportion of levels: FALSE 0.88, TRUE 0.12
NA count: 0
```

## symptom\_human\_coughing

**Summary statistics:**

```
 Variable: symptom_human_coughing 
 Class: logical
FALSE 91
TRUE 9
Mode: FALSE
Proportion of levels: FALSE 0.91, TRUE 0.09
NA count: 0
```

## symptom\_human\_bronchitis

**Summary statistics:**

```
 Variable: symptom_human_bronchitis 
 Class: logical
FALSE 99
TRUE 1
Mode: FALSE
Proportion of levels: FALSE 0.99, TRUE 0.01
NA count: 0
```

## symptom\_human\_pneumonia

**Summary statistics:**

```
 Variable: symptom_human_pneumonia 
 Class: logical
FALSE 99
TRUE 1
Mode: FALSE
Proportion of levels: FALSE 0.99, TRUE 0.01
NA count: 0
```

## symptom\_human\_fever

**Summary statistics:**

```
 Variable: symptom_human_fever 
 Class: logical
FALSE 96
TRUE 4
Mode: FALSE
Proportion of levels: FALSE 0.96, TRUE 0.04
NA count: 0
```

## symptom\_human\_headache

**Summary statistics:**

```
 Variable: symptom_human_headache 
 Class: logical
FALSE 96
TRUE 4
Mode: FALSE
Proportion of levels: FALSE 0.96, TRUE 0.04
NA count: 0
```

## symptom\_human\_myalgia

**Summary statistics:**

```
 Variable: symptom_human_myalgia 
 Class: logical
FALSE 97
TRUE 3
Mode: FALSE
Proportion of levels: FALSE 0.97, TRUE 0.03
NA count: 0
```

## symptom\_severity

**Summary statistics:**

```
 Variable: symptom_severity 
 Class: logical
FALSE 8
TRUE 2
Mode: FALSE
Proportion of levels: FALSE 0.80, TRUE 0.20
NA count: 90
```

## physician\_consultation

**Summary statistics:**

```
 Variable: physician_consultation 
 Class: logical
FALSE 98
TRUE 2
Mode: FALSE
Proportion of levels: FALSE 0.98, TRUE 0.02
NA count: 0
```

## flu\_vaccination

**Summary statistics:**

```
 Variable: flu_vaccination 
 Class: logical
FALSE 93
TRUE 7
Mode: FALSE
Proportion of levels: FALSE 0.93, TRUE 0.07
NA count: 0
```

## flu\_vaccination\_contacts

**Summary statistics:**

```
 Variable: flu_vaccination_contacts 
 Class: logical
FALSE 88
TRUE 12
Mode: FALSE
Proportion of levels: FALSE 0.88, TRUE 0.12
NA count: 0
```

## chronic\_disease\_condition

**Summary statistics:**

```
 Variable: chronic_disease_condition 
 Class: logical
FALSE 83
TRUE 17
Mode: FALSE
Proportion of levels: FALSE 0.83, TRUE 0.17
NA count: 0
```

## smoker

**Summary statistics:**

```
 Variable: smoker 
 Class: logical
FALSE 73
TRUE 27
Mode: FALSE
Proportion of levels: FALSE 0.73, TRUE 0.27
NA count: 0
```

## Age\_weeks\_factor

**Summary statistics:**

```
 Variable: Age_weeks_factor 
 Class: factor
weaners_4â5 55
weaners_6â7 17
weaners_8â9 4
weaners_10â12 12
weaners_mixed_ages 12
Mode: weaners_4â5
Proportion of levels: weaners_4â5 0.55, weaners_6â7 0.17, weaners_8â9 0.04, weaners_10â12 0.12, weaners_mixed_ages 0.12
Levels: weaners_4â5, weaners_6â7, weaners_8â9, weaners_10â12, weaners_mixed_ages
NA count: 0
```

## separation\_quarantine\_area

**Summary statistics:**

```
 Variable: separation_quarantine_area 
 Class: factor
1 8
2 14
3 76
NA's 2
Mode: 3
Proportion of levels: 1 0.08, 2 0.14, 3 0.78
Levels: 1, 2, 3
NA count: 2
```

## bird\_nests

**Summary statistics:**

```
 Variable: bird_nests 
 Class: logical
FALSE 79
TRUE 21
Mode: FALSE
Proportion of levels: FALSE 0.79, TRUE 0.21
NA count: 0
```

## farrowing\_piglets\_reduced\_general\_wellbeing

**Summary statistics:**

```
 Variable: farrowing_piglets_reduced_general_wellbeing 
 Class: logical
FALSE 84
TRUE 1
Mode: FALSE
Proportion of levels: FALSE 0.99, TRUE 0.01
NA count: 15
```

## farrowing\_piglets\_sneezing

**Summary statistics:**

```
 Variable: farrowing_piglets_sneezing 
 Class: numeric
Min.  0.000
1st Qu.  0.000
Median  0.000
Mean  4.647
3rd Qu.  8.000
Max. 33.000
NA's 15
Variance: NA
Shapiro-Wilk p-value: 0.0000000000008049 (not normal)
NA count: 15
```

## farrowing\_piglets\_coughing

**Summary statistics:**

```
 Variable: farrowing_piglets_coughing 
 Class: numeric
Min.  0.0000
1st Qu.  0.0000
Median  0.0000
Mean  0.8353
3rd Qu.  0.0000
Max. 17.0000
NA's 15
Variance: NA
Shapiro-Wilk p-value: 0.000000000000000003459 (not normal)
NA count: 15
```

## farrowing\_room\_temperature

**Summary statistics:**

```
 Variable: farrowing_room_temperature 
 Class: numeric
Min. 12.80
1st Qu. 17.65
Median 19.30
Mean 19.75
3rd Qu. 21.43
Max. 30.20
NA's 28
Variance: NA
Shapiro-Wilk p-value: 0.1633 (normal)
NA count: 28
```

## farrowing\_nest\_temperature\_ok

**Summary statistics:**

```
 Variable: farrowing_nest_temperature_ok 
 Class: logical
FALSE 14
TRUE 65
Mode: TRUE
Proportion of levels: FALSE 0.18, TRUE 0.82
NA count: 21
```

## farrowing\_airflow

**Summary statistics:**

```
 Variable: farrowing_airflow 
 Class: numeric
Min. 0.0000
1st Qu. 0.1000
Median 0.2000
Mean 0.1988
3rd Qu. 0.2050
Max. 1.0000
NA's 32
Variance: NA
Shapiro-Wilk p-value: 0.00000002934 (not normal)
NA count: 32
```

## farrowing\_air\_quality

**Summary statistics:**

```
 Variable: farrowing_air_quality 
 Class: factor
1 14
2 61
3 11
NA's 14
Mode: 2
Proportion of levels: 1 0.16, 2 0.71, 3 0.13
Levels: 1, 2, 3
NA count: 14
```

## farrowing\_airspace\_with\_other\_agegroup

**Summary statistics:**

```
 Variable: farrowing_airspace_with_other_agegroup 
 Class: factor
1 30
2 43
3 12
NA's 15
Mode: 2
Proportion of levels: 1 0.35, 2 0.51, 3 0.14
Levels: 1, 2, 3
NA count: 15
```

## ai\_sows\_room\_temperature

**Summary statistics:**

```
 Variable: ai_sows_room_temperature 
 Class: numeric
Min.  8.70
1st Qu. 15.05
Median 18.00
Mean 18.53
3rd Qu. 21.02
Max. 30.40
NA's 54
Variance: NA
Shapiro-Wilk p-value: 0.6843 (normal)
NA count: 54
```

## ai\_sows\_airflow

**Summary statistics:**

```
 Variable: ai_sows_airflow 
 Class: numeric
Min. 0.0200
1st Qu. 0.1175
Median 0.2000
Mean 0.2211
3rd Qu. 0.3000
Max. 0.5000
NA's 56
Variance: NA
Shapiro-Wilk p-value: 0.01042 (not normal)
NA count: 56
```

## ai\_sows\_air\_quality

**Summary statistics:**

```
 Variable: ai_sows_air_quality 
 Class: factor
1 10
2 39
3 9
NA's 42
Mode: 2
Proportion of levels: 1 0.17, 2 0.67, 3 0.16
Levels: 1, 2, 3
NA count: 42
```

## ai\_airspace\_with\_other\_agegroup

**Summary statistics:**

```
 Variable: ai_airspace_with_other_agegroup 
 Class: factor
1 7
2 25
3 25
NA's 43
Mode: 2
Proportion of levels: 1 0.12, 2 0.44, 3 0.44
Levels: 1, 2, 3
NA count: 43
```

## gestation\_sows\_qm\_per\_animal

**Summary statistics:**

```
 Variable: gestation_sows_qm_per_animal 
 Class: numeric
Min. 1.970
1st Qu. 2.500
Median 3.210
Mean 3.701
3rd Qu. 4.440
Max. 8.870
NA's 27
Variance: NA
Shapiro-Wilk p-value: 0.0000005447 (not normal)
NA count: 27
```

## gestation\_sows\_animals\_per\_water\_source

**Summary statistics:**

```
 Variable: gestation_sows_animals_per_water_source 
 Class: numeric
Min.  0.330
1st Qu.  6.150
Median  8.400
Mean  9.368
3rd Qu. 12.000
Max. 45.000
NA's 27
Variance: NA
Shapiro-Wilk p-value: 0.0000000104 (not normal)
NA count: 27
```

## gestation\_sows\_room\_temperature

**Summary statistics:**

```
 Variable: gestation_sows_room_temperature 
 Class: numeric
Min.  4.80
1st Qu. 14.10
Median 18.30
Mean 18.13
3rd Qu. 20.70
Max. 32.40
NA's 39
Variance: NA
Shapiro-Wilk p-value: 0.464 (normal)
NA count: 39
```

## gestation\_sows\_airflow

**Summary statistics:**

```
 Variable: gestation_sows_airflow 
 Class: numeric
Min. 0.0000
1st Qu. 0.1300
Median 0.2000
Mean 0.2692
3rd Qu. 0.3000
Max. 1.5000
NA's 41
Variance: NA
Shapiro-Wilk p-value: 0.0000000001037 (not normal)
NA count: 41
```

## gestation\_sows\_air\_quality

**Summary statistics:**

```
 Variable: gestation_sows_air_quality 
 Class: factor
1 24
2 43
3 5
NA's 28
Mode: 2
Proportion of levels: 1 0.33, 2 0.60, 3 0.07
Levels: 1, 2, 3
NA count: 28
```

## gestation\_sows\_airspace\_with\_other\_agegroup

**Summary statistics:**

```
 Variable: gestation_sows_airspace_with_other_agegroup 
 Class: factor
1 21
2 28
3 23
NA's 28
Mode: 2
Proportion of levels: 1 0.29, 2 0.39, 3 0.32
Levels: 1, 2, 3
NA count: 28
```

## weaners\_reduced\_general\_wellbeing

**Summary statistics:**

```
 Variable: weaners_reduced_general_wellbeing 
 Class: logical
FALSE 93
TRUE 1
Mode: FALSE
Proportion of levels: FALSE 0.99, TRUE 0.01
NA count: 6
```

## weaners\_sneezing

**Summary statistics:**

```
 Variable: weaners_sneezing 
 Class: numeric
Min.  0.000
1st Qu.  0.500
Median  3.185
Mean  5.996
3rd Qu.  7.825
Max. 53.300
NA's 6
Variance: NA
Shapiro-Wilk p-value: 0.0000000000008523 (not normal)
NA count: 6
```

## weaners\_coughing

**Summary statistics:**

```
 Variable: weaners_coughing 
 Class: numeric
Min. 0.0000
1st Qu. 0.0000
Median 0.0000
Mean 0.8978
3rd Qu. 0.8350
Max. 7.6000
NA's 6
Variance: NA
Shapiro-Wilk p-value: 0.00000000000001055 (not normal)
NA count: 6
```

## weaners\_discharge

**Summary statistics:**

```
 Variable: weaners_discharge 
 Class: logical
FALSE 91
TRUE 3
Mode: FALSE
Proportion of levels: FALSE 0.97, TRUE 0.03
NA count: 6
```

## weaners\_qm\_per\_animal

**Summary statistics:**

```
 Variable: weaners_qm_per_animal 
 Class: numeric
Min. 0.1800
1st Qu. 0.3100
Median 0.3700
Mean 0.4066
3rd Qu. 0.4400
Max. 1.2300
NA's 6
Variance: NA
Shapiro-Wilk p-value: 0.0000000008641 (not normal)
NA count: 6
```

## weaners\_animals\_per\_feeding\_site\_factor

**Summary statistics:**

```
 Variable: weaners_animals_per_feeding_site_factor 
 Class: factor
<20 27
<30 21
<40 21
>40 17
trough 8
NA's 6
Mode: <20
Proportion of levels: <20 0.29, <30 0.22, <40 0.22, >40 0.18, trough 0.09
Levels: <20, <30, <40, >40, trough
NA count: 6
```

## weaners\_animals\_per\_water\_source

**Summary statistics:**

```
 Variable: weaners_animals_per_water_source 
 Class: numeric
Min.  6.000
1st Qu.  9.762
Median 11.850
Mean 14.886
3rd Qu. 18.375
Max. 50.000
NA's 6
Variance: NA
Shapiro-Wilk p-value: 0.0000000001342 (not normal)
NA count: 6
```

## weaners\_room\_temperature

**Summary statistics:**

```
 Variable: weaners_room_temperature 
 Class: numeric
Min.  7.50
1st Qu. 16.80
Median 19.55
Mean 19.70
3rd Qu. 22.60
Max. 29.40
NA's 20
Variance: NA
Shapiro-Wilk p-value: 0.5194 (normal)
NA count: 20
```

## weaners\_airflow

**Summary statistics:**

```
 Variable: weaners_airflow 
 Class: numeric
Min. 0.0000
1st Qu. 0.1000
Median 0.1700
Mean 0.2074
3rd Qu. 0.2500
Max. 0.7000
NA's 23
Variance: NA
Shapiro-Wilk p-value: 0.0000003241 (not normal)
NA count: 23
```

## weaners\_air\_quality

**Summary statistics:**

```
 Variable: weaners_air_quality 
 Class: factor
1 6
2 79
3 9
NA's 6
Mode: 2
Proportion of levels: 1 0.06, 2 0.84, 3 0.10
Levels: 1, 2, 3
NA count: 6
```

## weaners\_airspace\_with\_other\_agegroup

**Summary statistics:**

```
 Variable: weaners_airspace_with_other_agegroup 
 Class: factor
2 23
3 52
4 19
NA's 6
Mode: 3
Proportion of levels: 2 0.24, 3 0.55, 4 0.20
Levels: 2, 3, 4
NA count: 6
```

## fattening\_pigs\_reduced\_general\_wellbeing

**Summary statistics:**

```
 Variable: fattening_pigs_reduced_general_wellbeing 
 Class: logical
FALSE 46
TRUE 1
Mode: FALSE
Proportion of levels: FALSE 0.98, TRUE 0.02
NA count: 53
```

## fattening\_pigs\_sneezing

**Summary statistics:**

```
 Variable: fattening_pigs_sneezing 
 Class: numeric
Min.  0.000
1st Qu.  0.000
Median  0.000
Mean  2.885
3rd Qu.  6.700
Max. 15.000
NA's 87
Variance: NA
Shapiro-Wilk p-value: 0.0002225 (not normal)
NA count: 87
```

## fattening\_pigs\_coughing

**Summary statistics:**

```
 Variable: fattening_pigs_coughing 
 Class: numeric
Min. 0.0000
1st Qu. 0.0000
Median 0.0000
Mean 0.5769
3rd Qu. 0.0000
Max. 4.8000
NA's 87
Variance: NA
Shapiro-Wilk p-value: 0.000005377 (not normal)
NA count: 87
```

## fattening\_pigs\_qm\_per\_animal

**Summary statistics:**

```
 Variable: fattening_pigs_qm_per_animal 
 Class: numeric
Min. 0.3000
1st Qu. 0.6725
Median 0.9450
Mean 1.0985
3rd Qu. 1.3150
Max. 4.7000
NA's 54
Variance: NA
Shapiro-Wilk p-value: 0.00000005605 (not normal)
NA count: 54
```

## fattening\_pigs\_feeding\_site\_per\_animal\_factor

**Summary statistics:**

```
 Variable: fattening_pigs_feeding_site_per_animal_factor 
 Class: factor
under 25 Animals/feeder 8
over 25 Animals/feeder 5
under 25cm trough/animal 10
over 25cm trough/animal 21
Other 3
NA 53
Mode: NA
Proportion of levels: under 25 Animals/feeder 0.08, over 25 Animals/feeder 0.05, under 25cm trough/animal 0.10, over 25cm trough/animal 0.21, Other 0.03, NA 0.53
Levels: under 25 Animals/feeder, over 25 Animals/feeder, under 25cm trough/animal, over 25cm trough/animal, Other, NA
NA count: 0
```

## fattening\_pigs\_animals\_per\_water\_source

**Summary statistics:**

```
 Variable: fattening_pigs_animals_per_water_source 
 Class: numeric
Min.  3.00
1st Qu.  8.65
Median 12.00
Mean 13.47
3rd Qu. 17.10
Max. 33.50
NA's 53
Variance: NA
Shapiro-Wilk p-value: 0.01312 (not normal)
NA count: 53
```

## fattening\_pigs\_room\_temperature

**Summary statistics:**

```
 Variable: fattening_pigs_room_temperature 
 Class: numeric
Min.  2.90
1st Qu. 16.57
Median 19.85
Mean 19.48
3rd Qu. 22.23
Max. 31.50
NA's 60
Variance: NA
Shapiro-Wilk p-value: 0.1944 (normal)
NA count: 60
```

## fattening\_pigs\_airflow

**Summary statistics:**

```
 Variable: fattening_pigs_airflow 
 Class: numeric
Min. 0.0400
1st Qu. 0.1500
Median 0.2000
Mean 0.2372
3rd Qu. 0.3000
Max. 0.6000
NA's 61
Variance: NA
Shapiro-Wilk p-value: 0.001534 (not normal)
NA count: 61
```

## fattening\_pigs\_air\_quality

**Summary statistics:**

```
 Variable: fattening_pigs_air_quality 
 Class: factor
1 4
2 32
3 11
NA's 53
Mode: 2
Proportion of levels: 1 0.09, 2 0.68, 3 0.23
Levels: 1, 2, 3
NA count: 53
```

## fattening\_pigs\_airspace\_with\_other\_agegroup

**Summary statistics:**

```
 Variable: fattening_pigs_airspace_with_other_agegroup 
 Class: factor
1 9
2 16
3 13
4 9
NA's 53
Mode: 2
Proportion of levels: 1 0.19, 2 0.34, 3 0.28, 4 0.19
Levels: 1, 2, 3, 4
NA count: 53
```

## report\_killed\_weaners

**Summary statistics:**

```
 Variable: report_killed_weaners 
 Class: logical
FALSE 97
TRUE 3
Mode: FALSE
Proportion of levels: FALSE 0.97, TRUE 0.03
NA count: 0
```

## report\_killed\_fattening\_pigs

**Summary statistics:**

```
 Variable: report_killed_fattening_pigs 
 Class: logical
FALSE 98
TRUE 2
Mode: FALSE
Proportion of levels: FALSE 0.98, TRUE 0.02
NA count: 0
```

## report\_killed\_young\_sows

**Summary statistics:**

```
 Variable: report_killed_young_sows 
 Class: logical
FALSE 99
TRUE 1
Mode: FALSE
Proportion of levels: FALSE 0.99, TRUE 0.01
NA count: 0
```

## report\_killed\_old\_sows

**Summary statistics:**

```
 Variable: report_killed_old_sows 
 Class: logical
FALSE 99
TRUE 1
Mode: FALSE
Proportion of levels: FALSE 0.99, TRUE 0.01
NA count: 0
```

# Session Information

```
## Generated by: jonasalexandersteiner
```

```
## Generated on: 2026-01-29 10:28:36
```

```
## Dataset dimensions: 100 rows x 186 columns
```

```
##  [1] "gridExtra"  "GGally"     "skimr"      "openxlsx"   "deeplr"     "kableExtra" "knitr"      "broom"      "DT"         "janitor"    "writexl"    "readxl"     "haven"      "lubridate" 
## [15] "forcats"    "stringr"    "purrr"      "readr"      "tidyr"      "tibble"     "tidyverse"  "scales"     "ggplot2"    "dplyr"      "pacman"
```
