# Supplementary material for: Epidemiology of Influenza A virus in Swiss pig herds: subclinical circulation and associated risk factors
Source: Porcine Health Manag. 2026 Apr 25;12:34. doi: 10.1186/s40813-026-00513-5 (PMC13261839; doi:10.1186/s40813-026-00513-5)
Supplement: Supplementary file 7 — Supplementary Material 7: Additional file 7. Description of data: Domain wise results of PLS-DA analyses presented with sample scores plot, RMSEP plot, risk and protective factors table with loadings on component 1 and 2 of the model, Variable Importance in Projection (VIP) scores table and model performance summary [file 40813_2026_513_MOESM7_ESM.html]

IAV Risk Factor Analysis: BinaryPLSR (PLS-DA)


# IAV Risk Factor Analysis: BinaryPLSR (PLS-DA)

#### jonasalexandersteiner

#### 2025-08-13

# Research Question & Hypothesis

This report investigates predictors (risk factors) for IAV positivity
in swine farms using BinaryPLSR (PLS-DA). The hypothesis is that
farm-level management, animal contact, and biosecurity variables are
associated with IAV status.

---

## Data Source & Preprocessing

- **Raw data file:** Data was loaded from
  `df3` (see analysis script for details).
- **Analysis script version, git commit, and random
  seed** used for model fitting and cross-validation are printed
  below for reproducibility.
- **Removed predictors log:** See
  `removed_predictors_log.csv` for details of all predictors
  filtered out at each stage.
- **Removed reference dummies log:** See
  `removed_reference_dummies_log.csv` for details of reference
  dummy columns dropped during encoding.
- **Preprocessing steps summary:**
  - Filtering and imputation (see analysis script and log files for
    details)
  - Dummy coding of categorical variables
  - Standardization of predictors
  - Any rows with missing outcome (`IAV_positive`) were
    removed

## Reproducibility: Script Version, Git Commit, and Random Seed

---

# PLS-DA Subset Analysis Overview

This report provides a separate PLS-DA analysis summary for each
predictor subset:

- **Husbandry:** Farm management, biosecurity, and
  husbandry practices.
- **Animal Health:** Animal health status and
  performance.
- **Environment:** Environmental and housing
  conditions.
- **Human/Contact:** Human illness history and
  direct/indirect contacts.

---


---

## Husbandry Predictors

### Analysis Summary

- **Subset:** Husbandry
- **Sample Size:** 100
- **Predictors after filtering:** 116
- **Optimal number of components:** 1
- **Variance explained by each component:** 6.84, 6.55,
  6.7 %
- **IAV Status Class Balance:**
  - Negative: 65 (65.0%)
  - Positive: 35 (35.0%)

### 1. PLS-DA Sample Scores Plot

### 2. RMSEP Plot (Model Validation)

### 3. Risk and Protective Factors

#### Component 1 Risk/Protective Factors

#### Component 2 Risk/Protective Factors

#### Variable Importance in Projection (VIP) Scores

### 4. Model Performance Summary

#### Confusion Matrix

Confusion Matrix

|  | FALSE | TRUE |
| --- | --- | --- |
| FALSE | 60 | 5 |
| TRUE | 16 | 19 |

#### Performance Metrics

Performance Metrics

| Metric | Value |
| --- | --- |
| Sensitivity | 0.543 |
| Specificity | 0.923 |
| Balanced Accuracy | 0.733 |
| AUC | 0.789 |

#### ROC Curve

---

## Animal Health Predictors

### Analysis Summary

- **Subset:** Animal Health
- **Sample Size:** 100
- **Predictors after filtering:** 50
- **Optimal number of components:** 1
- **Variance explained by each component:** 7.47, 11.87,
  4.33 %
- **IAV Status Class Balance:**
  - Negative: 65 (65.0%)
  - Positive: 35 (35.0%)

### 1. PLS-DA Sample Scores Plot

### 2. RMSEP Plot (Model Validation)

### 3. Risk and Protective Factors

#### Component 1 Risk/Protective Factors

#### Component 2 Risk/Protective Factors

#### Variable Importance in Projection (VIP) Scores

### 4. Model Performance Summary

#### Confusion Matrix

Confusion Matrix

|  | FALSE | TRUE |
| --- | --- | --- |
| FALSE | 57 | 8 |
| TRUE | 15 | 20 |

#### Performance Metrics

Performance Metrics

| Metric | Value |
| --- | --- |
| Sensitivity | 0.571 |
| Specificity | 0.877 |
| Balanced Accuracy | 0.724 |
| AUC | 0.792 |

#### ROC Curve

---

## Environment Predictors

### Analysis Summary

- **Subset:** Environment
- **Sample Size:** 100
- **Predictors after filtering:** 85
- **Optimal number of components:** 1
- **Variance explained by each component:** 5.46, 4.18,
  7.42 %
- **IAV Status Class Balance:**
  - Negative: 65 (65.0%)
  - Positive: 35 (35.0%)

### 1. PLS-DA Sample Scores Plot

### 2. RMSEP Plot (Model Validation)

### 3. Risk and Protective Factors

#### Component 1 Risk/Protective Factors

#### Component 2 Risk/Protective Factors

#### Variable Importance in Projection (VIP) Scores

### 4. Model Performance Summary

#### Confusion Matrix

Confusion Matrix

|  | FALSE | TRUE |
| --- | --- | --- |
| FALSE | 58 | 7 |
| TRUE | 14 | 21 |

#### Performance Metrics

Performance Metrics

| Metric | Value |
| --- | --- |
| Sensitivity | 0.600 |
| Specificity | 0.892 |
| Balanced Accuracy | 0.746 |
| AUC | 0.836 |

#### ROC Curve

---

## Human/Contact Predictors

### Analysis Summary

- **Subset:** Human/Contact
- **Sample Size:** 100
- **Predictors after filtering:** 12
- **Optimal number of components:** 1
- **Variance explained by each component:** 15.92, 23.24,
  8.62 %
- **IAV Status Class Balance:**
  - Negative: 65 (65.0%)
  - Positive: 35 (35.0%)

### 1. PLS-DA Sample Scores Plot

### 2. RMSEP Plot (Model Validation)

### 3. Risk and Protective Factors

#### Component 1 Risk/Protective Factors

#### Component 2 Risk/Protective Factors

#### Variable Importance in Projection (VIP) Scores

### 4. Model Performance Summary

#### Confusion Matrix

Confusion Matrix

|  | FALSE | TRUE |
| --- | --- | --- |
| FALSE | 61 | 4 |
| TRUE | 27 | 8 |

#### Performance Metrics

Performance Metrics

| Metric | Value |
| --- | --- |
| Sensitivity | 0.229 |
| Specificity | 0.938 |
| Balanced Accuracy | 0.584 |
| AUC | 0.631 |

#### ROC Curve

---

# Limitations

- Results depend on data preprocessing, filtering, and outcome coding
  (see logs for details).
- PLS-DA maximizes class separation rather than explained
  variance.
- Sample size and class imbalance may affect model stability.
- Component 2 may capture secondary patterns requiring further
  investigation.
- For very large datasets, further optimization (vectorized
  operations, data.table package) may be warranted.

---

# Reproducibility: R Session Info

```
## R version 4.5.1 (2025-06-13 ucrt)
## Platform: x86_64-w64-mingw32/x64
## Running under: Windows 11 x64 (build 22631)
## 
## Matrix products: default
##   LAPACK version 3.12.1
## 
## locale:
## [1] LC_COLLATE=German_Switzerland.utf8  LC_CTYPE=en_US.UTF-8                LC_MONETARY=German_Switzerland.utf8 LC_NUMERIC=C                        LC_TIME=C                          
## 
## time zone: Europe/Zurich
## tzcode source: internal
## 
## attached base packages:
## [1] grid      stats     graphics  grDevices utils     datasets  methods   base     
## 
## other attached packages:
##  [1] rmarkdown_2.29          rms_8.0-0               Hmisc_5.2-3             ResourceSelection_0.3-6 car_3.1-3               carData_3.0-5           pROC_1.18.5             viridis_0.6.5          
##  [9] viridisLite_0.4.2       mixOmics_6.32.0         MASS_7.3-65             caret_7.0-1             lattice_0.22-7          htmltools_0.5.8.1       ggbeeswarm_0.7.2        gridExtra_2.3          
## [17] GGally_2.3.0            skimr_2.2.1             openxlsx_4.2.8          deeplr_2.1.0            kableExtra_1.4.0        knitr_1.50              broom_1.0.8             DT_0.33                
## [25] janitor_2.2.1           writexl_1.5.4           readxl_1.4.5            haven_2.5.5             lubridate_1.9.4         forcats_1.0.0           stringr_1.5.1           purrr_1.1.0            
## [33] readr_2.1.5             tidyr_1.3.1             tibble_3.3.0            tidyverse_2.0.0         scales_1.4.0            ggplot2_3.5.2           dplyr_1.1.4             pacman_0.5.1           
## 
## loaded via a namespace (and not attached):
##   [1] splines_4.5.1        polspline_1.1.25     cellranger_1.1.0     hardhat_1.4.1        rpart_4.1.24         lifecycle_1.0.4      globals_0.18.0       vroom_1.6.5          crosstalk_1.2.1     
##  [10] backports_1.5.0      SnowballC_0.7.1      magrittr_2.0.3       sass_0.4.10          jquerylib_0.1.4      yaml_2.3.10          zip_2.3.3            RColorBrewer_1.1-3   multcomp_1.4-28     
##  [19] abind_1.4-8          TH.data_1.1-4        nnet_7.3-20          sandwich_3.1-1       ipred_0.9-15         lava_1.8.1           ggrepel_0.9.6        tokenizers_0.3.0     listenv_0.9.1       
##  [28] ellipse_0.5.0        MatrixModels_0.5-4   RSpectra_0.16-2      parallelly_1.45.0    svglite_2.2.1        codetools_0.2-20     xml2_1.3.8           tidyselect_1.2.1     farver_2.1.2        
##  [37] matrixStats_1.5.0    stats4_4.5.1         base64enc_0.1-3      jsonlite_2.0.0       Formula_1.2-5        survival_3.8-3       iterators_1.0.14     systemfonts_1.2.3    foreach_1.5.2       
##  [46] tools_4.5.1          ragg_1.4.0           Rcpp_1.1.0           glue_1.8.0           rARPACK_0.11-0       prodlim_2025.04.28   xfun_0.52            withr_3.0.2          fastmap_1.2.0       
##  [55] SparseM_1.84-2       digest_0.6.37        timechange_0.3.0     R6_2.6.1             textshaping_1.0.1    colorspace_2.1-1     utf8_1.2.6           generics_0.1.4       data.table_1.17.8   
##  [64] recipes_1.3.1        corpcor_1.6.10       class_7.3-23         httr_1.4.7           htmlwidgets_1.6.4    ggstats_0.10.0       ModelMetrics_1.2.2.2 pkgconfig_2.0.3      gtable_0.3.6        
##  [73] timeDate_4041.110    S7_0.2.0             gower_1.0.2          snakecase_0.11.1     rstudioapi_0.17.1    tzdb_0.5.0           reshape2_1.4.4       checkmate_2.3.2      nlme_3.1-168        
##  [82] repr_1.1.7           cachem_1.1.0         zoo_1.8-14           parallel_4.5.1       vipor_0.4.7          foreign_0.8-90       pillar_1.11.0        vctrs_0.6.5          cluster_2.1.8.1     
##  [91] beeswarm_0.4.0       htmlTable_2.4.3      evaluate_1.0.4       mvtnorm_1.3-3        cli_3.6.5            compiler_4.5.1       rlang_1.1.6          crayon_1.5.3         future.apply_1.20.0 
## [100] labeling_0.4.3       plyr_1.8.9           stringi_1.8.7        BiocParallel_1.42.1  quantreg_6.1         Matrix_1.7-3         hms_1.1.3            bit64_4.6.0-1        future_1.58.0       
## [109] igraph_2.1.4         bslib_0.9.0          bit_4.6.0
```

---

# End of Report

For complete transparency, please refer to the following files:

- **Analysis script:** See code version or git commit
  above.
- **Filtered predictors log:**
  `removed_predictors_log.csv`
- **Reference dummies log:**
  `removed_reference_dummies_log.csv`
- **Standardized predictor matrix:**
  `model_X_<subset>_used.csv`
- **Response variable:**
  `model_Y_<subset>_used.csv`

If you have questions or wish to audit intermediate steps, please
consult the logs and analysis script.
