# Supplementary material for: Epidemiology of Influenza A virus in Swiss pig herds: subclinical circulation and associated risk factors
Source: Porcine Health Manag. 2026 Apr 25;12:34. doi: 10.1186/s40813-026-00513-5 (PMC13261839; doi:10.1186/s40813-026-00513-5)
Supplement: Supplementary file 8 — Supplementary Material 8: Additional file 8. Description of data: Detailed results of the logistic regression of key factors within the husbandry subset are presented along with predictor selection, model coefficients, odds ratios and confidence intervals and model diagnostics with assessment of multicollinearity, outlier assessment and calibration [file 40813_2026_513_MOESM8_ESM.html]

IAV Key Exposures


Code 

- Show All Code
- Hide All Code

# IAV Key Exposures

#### jonasalexandersteiner

#### 2026-01-29

# Logistic regression of key factors in binary PLSR

This report presents summary results and visualizations for a
logistic regression model for IAV status in pig herds:

- Top 25 predictors by PLS-DA VIP were identified (husbandry
  subset).
- Univariate logistic regressions were computed for these predictors
  to assess their invidual influence on IAV detection.
- The final 4 predictors were chosen based on greatest VIP scores,
  then manually screened for biological plausibility. Variables reflecting
  intensive farming, missingness, or non-independence/confounding were
  excluded (see column ‘exclusion\_due\_to\_biological\_plausibility’ in
  tables).
- Multivariable logistic regression was performed with diagnostic
  checks and transparent reporting.
- Calibration of the model was assessed using the Hosmer-Lemeshow test
  and a calibration plot.

---

## Husbandry Subset Model

### Model Sample Size

N (Husbandry model used): 100

### Top 25 Predictors: Univariate OR Table

**Top 25 predictors by PLS-DA VIP, with univariate odds ratios,
95% confidence intervals, VIP scores, and biological exclusion
rationale. Effect size is VIP (distance from null effect). Variables
excluded for biological reasons are annotated.**

Top 25 by VIP with exclusion rationale

| variable | VIP | exclusion\_due\_to\_biological\_plausibility |
| --- | --- | --- |
| contact\_bird\_in\_stable\_TRUE | 3.37 |  |
| ai\_airspace\_with\_other\_agegroup\_2 | 3.23 | Confounding: proxy for intensive farming |
| ai\_airspace\_with\_other\_agegroup\_1 | 2.15 | Confounding: proxy for intensive farming |
| production\_cycle\_3 | 2.04 | Class imbalance: likely spurious finding |
| cleaning\_weaner\_stable\_FALSE | 2.04 | Confounding: proxy for intensive farming |
| number\_suckling\_piglets | 1.95 | No effect in logistic regression (exclude) |
| caretaker\_entry\_ppe\_only\_TRUE | 1.86 | Confounding: proxy for intensive farming |
| bird\_nests\_FALSE | 1.84 | Confounding: proxy for intensive farming |
| farrowing\_airspace\_with\_other\_agegroup\_2 | 1.79 | Confounding: proxy for intensive farming |
| cross\_fostering\_farrowing\_stable\_3 | 1.78 |  |
| weaners\_airspace\_with\_other\_agegroup\_3 | 1.70 |  |
| fattening\_pigs\_airspace\_with\_other\_agegroup\_4 | 1.68 | Confounding: proxy for intensive farming |
| production\_cycle\_2 | 1.59 | Class imbalance: likely spurious finding |
| proximity\_to\_other\_poultry\_herd\_FALSE | 1.57 | Confounding: proxy for intensive farming |
| farrowing\_airspace\_with\_other\_agegroup\_3 | 1.52 | Confounding: proxy for intensive farming |
| outside\_area\_contact\_wild\_boars\_FALSE | 1.46 | Confounding: proxy for intensive farming |
| number\_weaners | 1.42 | No effect in logistic regression (exclude) |
| separation\_quarantine\_area\_2 | 1.35 | Confounding: proxy for intensive farming |
| cleaning\_shipment\_area\_TRUE | 1.29 | Confounding: proxy for intensive farming |
| cattle\_closeby\_FALSE | 1.28 | Confounding: proxy for intensive farming |
| outside\_area\_contact\_poultry\_TRUE | 1.27 | Confounding: proxy for intensive farming |
| herdsize | 1.26 | No effect in logistic regression (exclude) |
| caretaker\_ppe\_washing\_interval\_FALSE | 1.24 | Confounding: proxy for intensive farming |
| mode\_stable\_occupation\_ai\_centre\_TRUE | 1.24 | Confounding: proxy for intensive farming |
| proximity\_to\_other\_pig\_herd\_TRUE | 1.21 |  |

### Model Coefficients

**Logistic regression coefficients, standard errors, and
z-values. Model includes final 4 predictors.**

Coefficients (Husbandry Model)

| Predictor | Estimate | Std. Error | z value |
| --- | --- | --- | --- |
| (Intercept) | -2.174 | 0.588 | -3.696 |
| contact\_bird\_in\_stable\_TRUE | 1.506 | 0.479 | 3.142 |
| cross\_fostering\_farrowing\_stable\_3 | 0.260 | 0.554 | 0.470 |
| weaners\_airspace\_with\_other\_agegroup\_3 | 0.868 | 0.486 | 1.786 |
| proximity\_to\_other\_pig\_herd\_TRUE | 0.529 | 0.514 | 1.029 |

### Odds Ratios and Confidence Intervals

Odds Ratios & 95% CI (Husbandry Model)

| Predictor | Odds Ratio | 2.5 % | 97.5 % |
| --- | --- | --- | --- |
| (Intercept) | 0.114 | 0.032 | 0.332 |
| contact\_bird\_in\_stable\_TRUE | 4.510 | 1.806 | 11.990 |
| cross\_fostering\_farrowing\_stable\_3 | 1.297 | 0.429 | 3.842 |
| weaners\_airspace\_with\_other\_agegroup\_3 | 2.382 | 0.938 | 6.400 |
| proximity\_to\_other\_pig\_herd\_TRUE | 1.698 | 0.632 | 4.830 |

### Diagnostics Overview

- **Pseudo-R² (McFadden):** 0.121 — > 0.2 =
  reasonable; lower = weak fit.  
    Model fit is weak.
- **AUC (ROC Curve):** 0.726 — > 0.7 =
  acceptable, > 0.8 = good.  
    Discrimination is acceptable.

### Multicollinearity

**Variance Inflation Factor (VIF) for each predictor. VIF >
5 signals multicollinearity.**

Variance Inflation Factor (Husbandry Model)

| Predictor | VIF |
| --- | --- |
| contact\_bird\_in\_stable\_TRUE | 1.10 |
| cross\_fostering\_farrowing\_stable\_3 | 1.11 |
| weaners\_airspace\_with\_other\_agegroup\_3 | 1.12 |
| proximity\_to\_other\_pig\_herd\_TRUE | 1.06 |

### Top 5 Influential Observations

**Cook’s Distance for the top 5 most influential points; high
values may indicate outliers.**

Top 5 Cook’s Distance Values (Husbandry Model)

| Observation | Cook\_Distance |
| --- | --- |
| 11 | 0.036 |
| 15 | 0.036 |
| 53 | 0.036 |
| 76 | 0.036 |
| 86 | 0.036 |

### Visualizations

### Calibration

**Calibration of model predictions was assessed by the
Hosmer-Lemeshow test and a calibration plot.**

**Hosmer-Lemeshow Test:**   
Chi-squared = 3.2 , df = 7 , p-value
= 0.866   
A p-value > 0.05 suggests good
calibration.  
  
n=100 Mean absolute error=0.042 Mean squared error=0.00257 0.9 Quantile
of absolute error=0.087

---

## Interpretation Notes

- **Exploratory Analysis:** No p-values or statistical
  significance are interpreted for selection, but all effect sizes and CIs
  are transparently reported.
- **Biological plausibility exclusion:** See the
  ‘exclusion\_due\_to\_biological\_plausibility’ column in tables for
  rationale for exclusion.
- **Model Fit:** Consider pseudo-R² and AUC for overall
  model quality.
- **Calibration:** Hosmer-Lemeshow and calibration curve
  should be checked for model reliability.
- **Multicollinearity:** High VIFs may require model
  simplification.
- **Influential Points:** Consider sensitivity analysis
  excluding high Cook’s D observations.

---

## Session Info

```
## R version 4.5.1 (2025-06-13 ucrt)
## Platform: x86_64-w64-mingw32/x64
## Running under: Windows 11 x64 (build 22631)
## 
## Matrix products: default
##   LAPACK version 3.12.1
## 
## locale:
## [1] LC_COLLATE=German_Switzerland.utf8  LC_CTYPE=en_US.UTF-8                LC_MONETARY=German_Switzerland.utf8 LC_NUMERIC=C                        LC_TIME=C                          
## 
## time zone: Europe/Zurich
## tzcode source: internal
## 
## attached base packages:
## [1] grid      stats     graphics  grDevices utils     datasets  methods   base     
## 
## other attached packages:
##  [1] rmarkdown_2.29          rms_8.0-0               Hmisc_5.2-3             ResourceSelection_0.3-6 car_3.1-3               carData_3.0-5           pROC_1.18.5             viridis_0.6.5          
##  [9] viridisLite_0.4.2       mixOmics_6.32.0         MASS_7.3-65             caret_7.0-1             lattice_0.22-7          htmltools_0.5.8.1       ggbeeswarm_0.7.2        gridExtra_2.3          
## [17] GGally_2.3.0            skimr_2.2.1             openxlsx_4.2.8          deeplr_2.1.0            kableExtra_1.4.0        knitr_1.50              broom_1.0.8             DT_0.33                
## [25] janitor_2.2.1           writexl_1.5.4           readxl_1.4.5            haven_2.5.5             lubridate_1.9.4         forcats_1.0.0           stringr_1.5.1           purrr_1.1.0            
## [33] readr_2.1.5             tidyr_1.3.1             tibble_3.3.0            tidyverse_2.0.0         scales_1.4.0            ggplot2_3.5.2           dplyr_1.1.4             pacman_0.5.1           
## 
## loaded via a namespace (and not attached):
##   [1] splines_4.5.1        polspline_1.1.25     cellranger_1.1.0     hardhat_1.4.1        rpart_4.1.24         lifecycle_1.0.4      globals_0.18.0       vroom_1.6.5          crosstalk_1.2.1     
##  [10] backports_1.5.0      SnowballC_0.7.1      magrittr_2.0.3       sass_0.4.10          jquerylib_0.1.4      yaml_2.3.10          zip_2.3.3            RColorBrewer_1.1-3   multcomp_1.4-28     
##  [19] abind_1.4-8          TH.data_1.1-4        nnet_7.3-20          sandwich_3.1-1       ipred_0.9-15         lava_1.8.1           ggrepel_0.9.6        tokenizers_0.3.0     listenv_0.9.1       
##  [28] ellipse_0.5.0        MatrixModels_0.5-4   RSpectra_0.16-2      parallelly_1.45.0    svglite_2.2.1        codetools_0.2-20     xml2_1.3.8           tidyselect_1.2.1     farver_2.1.2        
##  [37] matrixStats_1.5.0    stats4_4.5.1         base64enc_0.1-3      jsonlite_2.0.0       Formula_1.2-5        survival_3.8-3       iterators_1.0.14     systemfonts_1.2.3    foreach_1.5.2       
##  [46] tools_4.5.1          ragg_1.4.0           Rcpp_1.1.0           glue_1.8.0           rARPACK_0.11-0       prodlim_2025.04.28   xfun_0.52            withr_3.0.2          fastmap_1.2.0       
##  [55] SparseM_1.84-2       digest_0.6.37        timechange_0.3.0     R6_2.6.1             textshaping_1.0.1    colorspace_2.1-1     utf8_1.2.6           generics_0.1.4       data.table_1.17.8   
##  [64] recipes_1.3.1        corpcor_1.6.10       class_7.3-23         httr_1.4.7           htmlwidgets_1.6.4    ggstats_0.10.0       ModelMetrics_1.2.2.2 pkgconfig_2.0.3      gtable_0.3.6        
##  [73] timeDate_4041.110    S7_0.2.0             gower_1.0.2          snakecase_0.11.1     rstudioapi_0.17.1    tzdb_0.5.0           reshape2_1.4.4       checkmate_2.3.2      nlme_3.1-168        
##  [82] repr_1.1.7           cachem_1.1.0         zoo_1.8-14           parallel_4.5.1       vipor_0.4.7          foreign_0.8-90       pillar_1.11.0        vctrs_0.6.5          cluster_2.1.8.1     
##  [91] beeswarm_0.4.0       htmlTable_2.4.3      evaluate_1.0.4       mvtnorm_1.3-3        cli_3.6.5            compiler_4.5.1       rlang_1.1.6          crayon_1.5.3         future.apply_1.20.0 
## [100] labeling_0.4.3       plyr_1.8.9           stringi_1.8.7        BiocParallel_1.42.1  quantreg_6.1         Matrix_1.7-3         hms_1.1.3            bit64_4.6.0-1        future_1.58.0       
## [109] igraph_2.1.4         bslib_0.9.0          bit_4.6.0
```

---

Prepared by
**jonasalexandersteiner** on **2026-01-29**. Fl
